# Mapping global zoonotic niche and interregional transmission risk of monkeypox: a retrospective observational study

**DOI:** 10.1186/s12992-023-00959-0

**Published:** 2023-08-17

**Authors:** Yan-Qun Sun, Jin-Jin Chen, Mei-Chen Liu, Yuan-Yuan Zhang, Tao Wang, Tian-Le Che, Ting-Ting Li, Yan-Ning Liu, Ai-Ying Teng, Bing-Zheng Wu, Xue-Geng Hong, Qiang Xu, Chen-Long Lv, Bao-Gui Jiang, Wei Liu, Li-Qun Fang

**Affiliations:** 1 State Key Laboratory of Pathogen and Biosecurity, Beijing Institute of Microbiology and Epidemiology, 20 Dong-Da Street, Fengtai District, Beijing, 100071 China; 2 Nanjing Municipal Center for Disease Control and Prevention, Affiliated Nanjing Center for Disease Control and Prevention of Nanjing Medical University, Nanjing, China; 3 School of Public Health, Anhui Medical University, Hefei, 230032 China; 4 School of Public Health, Guizhou Medical University, Guiyang, 550025 China

**Keywords:** Monkeypox, Zoonotic niche, Machine learning, Transmission risk

## Abstract

**Background:**

Outbreaks of monkeypox have been ongoing in non-endemic countries since May 2022. A thorough assessment of its global zoonotic niche and potential transmission risk is lacking.

**Methods:**

We established an integrated database on global monkeypox virus (MPXV) occurrence during 1958 − 2022. Phylogenetic analysis was performed to examine the evolution of MPXV and effective reproductive number (R_t_) was estimated over time to examine the dynamic of MPXV transmissibility. The potential ecological drivers of zoonotic transmission and inter-regional transmission risks of MPXV were examined.

**Results:**

As of 24 July 2022, a total of 49 432 human patients with MPXV infections have been reported in 78 countries. Based on 525 whole genome sequences, two main clades of MPXV were formed, of which Congo Basin clade has a higher transmissibility than West African clade before the 2022-monkeypox, estimated by the overall R_t_ (0.81 vs. 0.56), and the latter significantly increased in the recent decade. R_t_ of 2022-monkeypox varied from 1.14 to 4.24 among the 15 continuously epidemic countries outside Africa, with the top three as Peru (4.24, 95% CI: 2.89–6.71), Brazil (3.45, 95% CI: 1.62–7.00) and the United States (2.44, 95% CI: 1.62–3.60). The zoonotic niche of MPXV was associated with the distributions of *Graphiurus lorraineus* and *Graphiurus crassicaudatus*, the richness of Rodentia, and four ecoclimatic indicators. Besides endemic areas in Africa, more areas of South America, the Caribbean States, and Southeast and South Asia are ecologically suitable for the occurrence of MPXV once the virus has invaded. Most of Western Europe has a high-imported risk of monkeypox from Western Africa, whereas France and the United Kingdom have a potential imported risk of Congo Basin clade MPXV from Central Africa. Eleven of the top 15 countries with a high risk of MPXV importation from the main countries of 2022-monkeypox outbreaks are located at Europe with the highest risk in Italy, Ireland and Poland.

**Conclusions:**

The suitable ecological niche for MPXV is not limited to Africa, and the transmissibility of MPXV was significantly increased during the 2022-monkeypox outbreaks. The imported risk is higher in Europe, both from endemic areas and currently epidemic countries. Future surveillance and targeted intervention programs are needed in its high-risk areas informed by updated prediction.

**Supplementary Information:**

The online version contains supplementary material available at 10.1186/s12992-023-00959-0.

## Introduction

Monkeypox is a zoonotic disease caused by infection with monkeypox virus (MPXV), which is endemic in Western and Central African countries. The current re-emergency since 7 May 2022 has been reported to influence at least 104 historical non-endemic countries in Europe (45 countries), America (31 countries), Asia (18 countries), Africa (13 countries) plus Oceania (4 countries), even causing 127 human deaths in 17 countries outside its previously reported endemic areas [1–3].

Novel mode of transmission, sexual transmission for the most part of infections (97.52% of the reported cases come from population of men who have sex with men), was distinct from previous interregional transmission such as travel-related importation from Africa or exposure to infected exotic pets [3, 4]. Considering its spread around the world rapidly through new modes of transmission and limited knowledge about it, the World Health Organization (WHO) has declared monkeypox a Public Health Emergency of International Concern (PHEIC) on July 23, 2022, which is the highest level of alert issued by the global health body [1, 5, 6].

Against this background, several queries remained to be addressed before national and global responses can be developed. Whether or not the spread of monkeypox is driven by a rise in the number of cases at the source in Western and Central Africa, how is the increased frequency of travel to and from endemic areas in Africa or epidemic areas in Europe was linked to the exported infections? And where might future outbreaks strike? Recent studies indicate the ecological niche of MPXV in Western Africa has spread across known ecological areas e.g., from the freshwater swamps and mangrove areas to savannah vegetation areas in the Nigeria 2017–18 outbreak, which call for further study by using updated data to might alter or refine niche modeling results [7]. In addition, the changing pattern of case predominance in urban areas indicated by the recent ongoing outbreaks may suggest the possibility of MPXV transmission from humans to local animal reservoirs, with in long-term consequences that should be further explored. This is a crucial concern for estimating the population at risk and for future public health measures.

Here by combining data from literature review and officially released reports, we assembled a dataset that contained all MPXV infections reports of both human and non-human sources since 1958 when the virus was identified [8]. A variety of environmental, socioeconomic, and biological factors were collected and applied in the model construction to predict areas at risk of monkeypox outbreaks. The inter-regional transmission risk of MPXV has also been examined by using the data from global annual air travel flows of origin–destination.

## Material and methods

### Database on MPXV

**Fig. 1:**
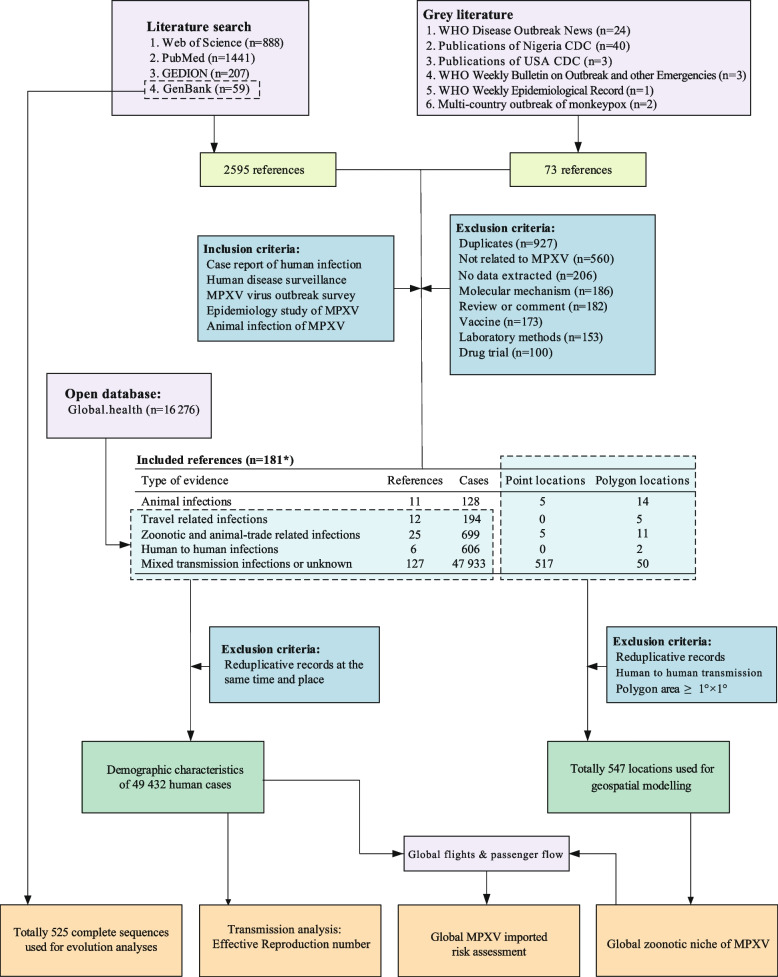
Flow chart of literature review. The data sources, inclusion and exclusion criteria, and analysis steps were marked by background of the purple, blue and yellow color, respectively

All publications that reported MPXV infections between 1958 and 24 July 2022 were collected via the following: (1) English databases, i.e., ISI Web of Science (WOS), PubMed database, GenBank database, and Global Infectious Diseases and Epidemiology Network (GIDEON); (2) grey literature which contained information from government, academics, or professional organizations i.e., WHO, USA Center for disease control and prevention (CDC), Nigeria Centre for Disease Control and Prevention (NCDC); and (3) public database: global.health. Throughout the searching process, we used a combination of controlled keywords: (“monkeypox virus”, OR “monkeypox”, OR “monkey pox”, OR “orthopoxvirus”) (Table S1 and S2). Titles and abstracts of retrieved studies were screened independently by two reviewers using pre-defined criteria to identify studies potentially eligible for inclusion (detailed in Table S3). Animal experiments, review articles, clinical trials and laboratory studies that did not provide raw data were excluded. The selected studies had the full texts retrieved and read through to assess for eligibility by two reviewers independently (Fig. 1). Articles suitable for extraction from the literature searches were case reports, outbreak investigations, epidemiological studies, and surveillance studies. The following information was extracted: article title, publish year, geographic information, age, gender of subjects, type of exposure, diagnostic laboratory procedure, and smallpox vaccination by using a standardized form (Table S4). For articles reporting results from the same dataset or the same outbreak event, we only kept the most recent one. The criteria for human infections were based on WHO, CDC and NCDC guidelines, and animal infections were based on World Organization for Animal Health guidelines (detailed in Table S5). For human infections, transmission routes were classified as zoonotic transmission or human-to-human transmission according to CDC criteria (detailed in Table S5) [9].

### Phylodynamic and transmissibility analyses of MPXV

A total of 525 full-length genomes of the MPXV reported between 1958 and 22 July 2022, together with their temporal and geographic information were obtained from the GenBank. Sequences were assembled and aligned using MAFFT v7.407 web server, and the trimmed sequences were mapped to a reference genome (Accession: NC_003310 and NC_063383) [10]. For the nucleotide phylogenetic analysis, chronological and geographical data regarding the genome records were used. The phylogenetic trees were generated using the maximum-likelihood method with 1000 bootstrap replicates on RaxML web server [11]. The phylogenetic tree was edited and visualized in the Figtree software (http://tree.bio.ed.ac.uk/software/figtree). Details of the phylodynamic analyses are given in Text S1.

The effective reproduction number (R_t_) for monkeypox was projected from 1990 to 2020 using a method for subcritical outbreaks of infectious diseases comprising both zoonotic and human-to-human transmission, for which the R_t_ was often less than 1. As indicated before, the upper bound of R_t_ was estimated as one minus the fraction of cases infected by the reservoir out of the total number of identified cases [12, 13]. The counts of all reported cases and patients infected via the reservoirs were used to calculate the R_t_ for monkeypox outbreaks from 1990 to 2020 and are presented in Table S7. Based on a Bayesian approach that depends on the time series and the distribution of serial interval, the R_t_ of the current monkeypox outbreaks in 2022 was predicted using the number of confirmed cases between 6 May and 22 July in the global.health database [14]. In the modeling analysis, we assumed a mean and standard deviation of the serial interval of 9.7 and 2.7 days, respectively [15]. The R_t_ of the current outbreaks in 2022 was estimated by using the R package “EpiEstim” [16]. Details of the procedure for the estimation of R_t_ are given in Text S2.

### Geo-reference and modeling of MPXV

We obtained the exact locations and coordinates of MPXV occurrences in both humans and animals using Google Map (http://maps.google.com), composed into the geo-tagged database of MPXV occurrences from 1972–2019. A total of 39 variables were hypothesized to be potentially related to MPXV occurrence, including 31 environmental-related, 5 human activity-related, and 3 animal-related variables, which were collected (detailed in Table S9 and S10). The inclusion of environmental variables was based on their relevance to the ecology and transmission dynamics of MPXV (detailed in Table S11). We considered factors such as temperature, precipitation, land cover, elevation, and latitude, as these variables may have been shown to affect the distribution and abundance of rodent hosts, as well as the survival and transmission of the virus [17]. By including these environmental variables, we aimed to explore their potential association with the occurrence of MPXV and assess their significance in shaping the zoonotic niche of the virus.

Based on previous knowledge on the animal reservoirs of MPXV [18–21], and the currently collected data on the animal species with positive MPXV detection, we determined four animal species, i.e., *Funisciurus* spp., *Graphiurus crassicaudatus*, *Graphiurus lorraineus*, and *Cricetomys gambianus*, as competent animals that can impact on MPXV incidence (details of the definition of MPXV infection in potential reservoir hosts are given in Table S8, and previous studies including these variables are given in Table S11). To incorporate this potential driver of the zoonotic transmission of MPXV, we first predicted the distributions of potential animal reservoirs associated with MPXV infections. The occurrence data of these animal reservoirs (classified as animal-related variables) were collected from Global Biodiversity Information Facility (GBIF) (https://www.gbif.org) (detailed in Table S12), and were adjusted using the data from International Union for Conservation of Nature (IUCN) (https://www.iucn.org).

The analyzed variables were computed for their multicollinearity by pairwise Pearson correlations. For each pair of variables with a Pearson correlation coefficient above 0.70, the variables with a highest average correlation with all other variables were omitted from analysis. Utilizing a case–control research approach, a boosted regression tree (BRT) model was used to examine the zoonotic niche of MPXV in Africa and determine its ecological adaptability in the worldwide range. The primary analysis utilized a tree complexity of five, a learning rate of 0.005 and a bagging fraction of 75% based on their previously validated good performance [22, 23]. Using a two-stage bootstrapping approach, the model parameters were estimated in a more precise and economical manner. BRT modeling was undertaken using the R packages “dismo” and “gbm”, and predictive performance of the model was evaluated using R packages “ROCR” and “pROC” (Detailed in the Text S3) [24, 25].

To predict the high-risk regions for MPXV zoonotic transmission, we determined the cut-off value with optimal sensitivity + specificity along the receiver operating characteristic curve (ROC) for the final BRT model. For grids where the estimated probability is larger than the cut-off of the model, a significant risk of zoonotic MPXV infections was established. The total population size of the regions with the predicted zoonotic niche of MPXV was considered as vulnerable to MPXV. The native presence/absence niche model was established by using the occurrence records of MPXV as the outcome variable, covariates derived from MPXV endemic locations (Central Africa and Western Africa) used as explanatory variables. Global range prediction was performed by applying the global explanatory covariates to determine the ecologically suitable regions. In addition, we calculated the risks of worldwide dissemination for MPXV based on the fraction of population at risk in endemic and major epidemic countries (> 60 confirmed cases) and origin–destination (OD) yearly air travel flows (detailed in Text S4).

## Results

### Database assembly

A total of 2668 literature (including 73 grey literature) available from 1 January 1968 to 24 July 2022 were reviewed. Based on our inclusion/exclusion criteria, 181 research articles were retained for data retrieval and analysis. Data on additional 16 276 monkeypox cases reported from the global.health MPXV database as of 24 July 2022, were collected (Fig. 1). Taken together, a total of 49 432 human cases from 78 countries in six regions were included in the final database (Table 1 and Table S16). Laboratory confirmed diagnosis was made in 37.29% of the cases, and 86.55% of the patients with available baseline information were males. The majority of confirmed cases occurred in children (64.82%), followed by young adults (19.76%) and adults (15.26%). Zoonotic transmission was observed in 40.56% of cases, followed by community transmission (21.35%), travel-related infection (12.94%), probable men who have sex with men (MSM)-related infection (10.87%), household transmission (7.54%), animal trade (6.07%), and nosocomial infection (0.67%). Cases in the Central Africa were mostly of zoonotic transmission (70.30%), while suspected MSM-related infection was most frequently seen in European cases (39.78%), and American cases were dominated by animal trade infection (44.85%). In addition, 75.64% cases had no history of smallpox vaccination, and 45.10%, 26.55%, and 25.52% of cases were reported to be infected by exposure to rodents, monkey, and chimpanzee, respectively, while 2.84% cases were infected by exposure to gazelles, porcupines, unspecified domestic or wild animals (Table 1). The demographic characteristics and distributional dynamics of MPXV infections were detailed in Text S5, Figure S1-S3.

**Table 1:** Epidemiological features of global human monkeypox patients recorded during 1970 − 24 July 2022

Features	All	Central Africa	Western Africa	Other Regions in Africa^a^	Europe	America	Other areas
Total	49,432	31,815	1246	34	11,992	4162	183
Type							
Confirmed	18,433 (37.29%)	1823 (5.73%)	363 (29.13%)	7 (20.59%)	11,989 (99.97%)	4072 (97.84%)	179 (97.81%)
Suspect or probable	30,999 (62.71%)	29,992 (94.27%)	883 (70.87%)	27 (79.41%)	3 (0.03%)	90 (2.16%)	4 (2.19%)
Gender^b^							
Male	2838 (86.55%)	998 (80.29%)	254 (68.83%)	12 (50.00%)	1038 (99.05%)	505 (89.7%)	31 (96.88%)
Female	441 (13.45%)	245 (19.71%)	115 (31.17%)	12 (50.00%)	10 (0.95%)	58 (10.3%)	1 (3.13%)
Age^b^							
0–14	3127 (64.82%)	3068 (76.32%)	34 (9.97%)	15 (60.00%)	2 (1.10%)	7 (3.02%)	1 (4.00%)
15–29	953 (19.76%)	671 (16.69%)	252 (73.90%)	7 (28.00%)	7 (3.87%)	15 (6.47%)	1 (4.00%)
30–59	736 (15.26%)	276 (6.87%)	55 (16.13%)	3 (12.00%)	169 (93.37%)	210 (90.52%)	23 (92.00%)
≥ 60	8 (0.17%)	5 (0.12%)	0 (0.00%)	0 (0.00%)	3 (1.66%)	0 (0.00%)	0 (0.00%)
Testing Method^bc^							
PCR	348 (92.31%)	218 (91.6%)	5 (50.00%)	3 (100%)	92 (95.83%)	26 (100.00%)	4 (100.00%)
Next generation sequencing	20 (5.31%)	18 (7.56%)	0 (0.00%)	0 (0.00%)	2 (2.08%)	0 (0.00%)	0 (0.00%)
Virus isolation	7 (1.86%)	2 (0.84%)	5 (50.00%)	0 (0.00%)	0 (0.00%)	0 (0.00%)	0 (0.00%)
Electron microscope	2 (0.53%)	0 (0.00%)	0 (0.00%)	0 (0.00%)	2 (2.08%)	0 (0.00%)	0 (0.00%)
Inpatient^b^							
Yes	225 (54.88%)	57 (95.00%)	41 (100%)	8 (42.11%)	70 (45.16%)	34 (29.06%)	15 (83.33%)
No	185 (45.12%)	3 (5.00%)	0 (0.00%)	11 (57.89%)	85 (54.84%)	83 (70.94%)	3 (16.67%)
Transmission model^b^							
Zoonotic infection	608 (40.56%)	594 (70.30%)	14 (31.11%)	0 (0.00%)	0 (0.00%)	0 (0.00%)	0 (0.00%)
Community transmission	320 (21.35%)	153 (18.11%)	19 (42.22%)	6 (27.27%)	135 (36.29%)	7 (3.61%)	0 (0.00%)
Travel-related infection	194 (12.94%)	0 (0.00%)	3 (6.67%)	4 (18.18%)	81 (21.77%)	85 (43.81%)	21 (100.00%)
Probable MSM-related infection	163 (10.87%)	0 (0.00%)	0 (0.00%)	0 (0.00%)	148 (39.78%)	15 (7.73%)	0 (0.00%)
Household transmission	113 (7.54%)	93 (11.01%)	8 (17.78%)	8 (36.36%)	4 (1.08%)	0 (0.00%)	0 (0.00%)
Infections via animal trade	91 (6.07%)	0 (0.00%)	0 (0.00%)	3 (13.64%)	1 (0.27%)	87 (44.85%)	0 (0.00%)
Nosocomial infection	10 (0.67%)	5 (0.59%)	1 (2.22%)	1 (4.55%)	3 (0.81%)	0 (0.00%)	0 (0.00%)
Smallpox vaccination^b^							
No	444 (75.64%)	424 (75.44%)	7 (87.50%)	4 (100%)	6 (85.71%)	3 (50.00%)	0 (0.00%)
Yes	143 (24.36%)	138 (24.56%)	1 (12.50%)	0 (0%)	1 (14.29%)	3 (50.00%)	0 (0.00%)
Animal contact history^b^							
Rodent	175 (45.10%)	84 (29.47%)	3 (25.00%)	0 (0.00%)	0 (0.00%)	87 (100.00%)	1 (100.00%)
Monkey	103 (26.55%)	101 (35.44%)	2 (16.67%)	0 (0.00%)	0 (0.00%)	0 (0.00%)	0 (0.00%)
Chimpanzee	99 (25.52%)	99 (34.74%)	0 (0.00%)	0 (0.00%)	0 (0.00%)	0 (0.00%)	0 (0.00%)
Other animals	11 (2.84%)	1 (0.35%)	7 (58.33%)	3 (100.00%)	0 (0.00%)	0 (0.00%)	0 (0.00%)
Outcome^b^							
Recover	10,183 (97.23%)	8894 (96.96%)	1215 (99.10%)	19 (100.00%)	13 (100%)	39 (100.00%)	3 (100.00%)
Death	288 (2.77%)	279 (3.04%)	11 (0.90%)	0 (0.00%)	0 (0.00%)	0 (0.00%)	0 (0.00%)
Number of cases							
1970–1979	53 (0.11%)	44 (0.14%)	9 (0.72%)	0 (0.00%)	0 (0.00%)	0 (0.00%)	0 (0.00%)
1980–1989	116 (0.23%)	115 (0.36%)	1 (0.08%)	0 (0.00%)	0 (0.00%)	0 (0.00%)	0 (0.00%)
1990–1999	549 (1.11%)	549 (1.73%)	0 (0.00%)	0 (0.00%)	0 (0.00%)	0 (0.00%)	0 (0.00%)
2000–2009	10,150 (20.53%)	10,044 (31.57%)	0 (0.00%)	19 (55.88%)	0 (0.00%)	87 (2.09%)	0 (0.00%)
2010–2019	17,063 (34.52%)	16,445 (51.69%)	612 (49.12%)	0 (0.00%)	4 (0.03%)	0 (0.00%)	2 (1.09%)
2020- July 2022	21,501 (43.50%)	4618 (14.52%)	624 (50.08%)	15 (44.12%)	11,988 (99.97%)	4075 (97.91%)	181 (98.91%)

^a^Other Regions in Africa: Eastern Africa, Southern Africa and Northern Africa

^b^Data are included with specific information reported

^c^Only confirmed cases are included

### Evolution and transmissibility of MPXV

**Fig. 2:**
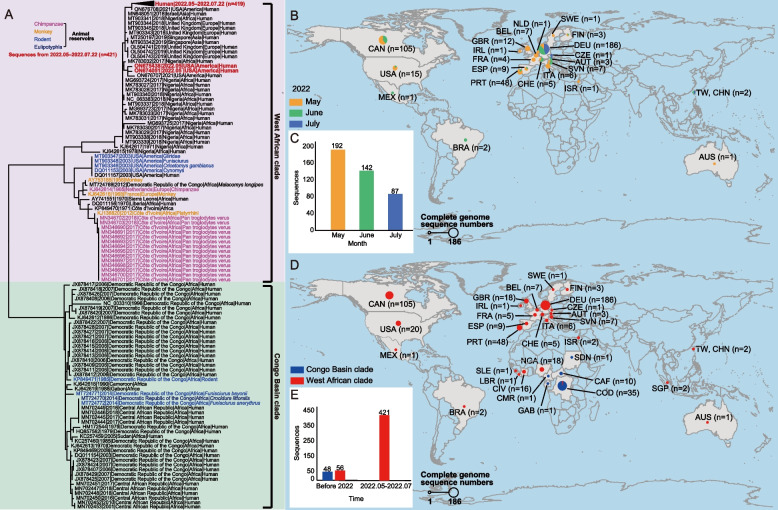
The nucleotide phylogenetic trees and geographical distribution of MPXV strains reported. **A** Maximum likelihood tree of MPXV strains based on the 525 full-genome sequence, 419 of which from 2022 were folded; **B** Locations and numbers of 421 reported full-complete genome sequences for 2022, as well as the reported month of full-complete sequences, were shown with orange, green and blue, respectively; **C** Histogram of sequence discovery month in 2022 (the total number of sequences in 2022 is 421); **D** Locations and numbers of full-complete genome sequences for West African clade and Congo Basin clade reported. The clades of full-complete sequence were shown by the colors indigo and violet, respectively; **E** Histogram of sequence discovery interval and clade (the total number of sequences before 2022 is 104). The country or region abbreviations were listed with full name in Table S26

Between 1 January1958 and 22 July 2022, a total of 525 full-genome sequences of MPXV were uploaded to GenBank (104 before 2022 and 421 during 2022). Among them 495 were derived from human cases, 15 from chimpanzees, seven from rodents, three from monkeys, one from Eulipotyphla, and four with unknown source. The phylogenetic tree indicated the grouping of two major MPXV clades related with human infection: Congo Basin clade and West African clade. The phylogenetic analysis revealed that the MPXV obtained in 2022 belonged to West African clade and was most closely linked to the MPXV identified in 2021 in USA (Fig. 2A and Table S6). The sequences obtained prior to 2022 were evenly grouped in Congo Basin clade and West Africa clade (48 and 56, respectively) (Fig. 2A, C, E). Full-genome sequences of MPVX were reported from six continents (32 countries or regions), with the highest proportion derived from Germany (35.43%), Canada (20.00%), Portugal (9.14%), and Democratic Republic of the Congo (DRC, at the time known as Zaire, 5.90%) (Fig. 2A, D). A strong geographic dependent grouping of the clades with observed, with MPXV of Congo Basin clade most commonly reported from DRC, Cameroon, Gabon, Sudan, and Central African Republic (Fig. 2D).

In order to assess the transmissibility of MPXV, the upper limits on R_t_ for MPXV were calculated by epidemic periods and viral clades. Compared with West African clade, the R_t_ of Congo Basin clade was significantly higher in overall study period (0.81 vs. 0.56, *p* < 0.001) and in 2000–2009 (0.83 vs. 0.48, *p* = 0.001), while R_t_ of West African clade increased by years from 0.48 in 2000–2009 to 0.75 in 2010–2017, becoming almost comparable with that of Congo Basin clade during 2010–2017 (0.75 vs. 0.85, *p* = 0.308). Based on the confirmed cases in the main affected countries, R_t_ of 2022-monkeypox was estimated to range from 1.14 to 4.24, and the five countries with top ranking R_t_ were: Peru (R_t_ = 4.24, 95% CI: 2.89–6.71), Brazil (R_t_ = 3.45, 95% CI: 1.62–7.00), United States (R_t_ = 2.44, 95% CI: 1.62–3.60), Germany (R_t_ = 2.12, 95% CI: 1.13–5.20), and France (R_t_ = 1.98, 95% CI: 1.07–5.69) (Figure S4).

### Predicted environmental suitability for zoonotic transmission of MPXV

**Fig. 3:**
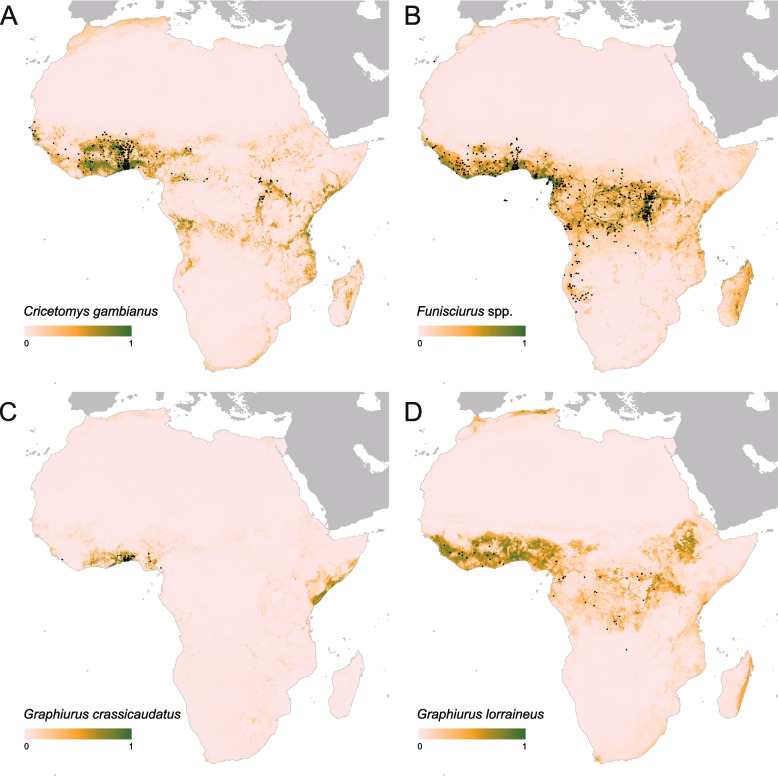
Predicted geographical distribution of the four species of Rodent suspected to reservoir of MPXV: (**A**) *Cricetomys gambianus*, (**B**) *Funisciurus* spp., (**C**) *Graphiurus crassicaudatus*, and (**D**) *Graphiurus lorraineus*. On each panel, the locations of reported observations of each species were extracted and curated from the Global Biodiversity Information Facility (GBIF, 2022) as black points. The color legend represented a scale of the relative probability that the species occurs in that location from 0 (white, low) to 1 (green, high)

The potential ranges of four major animal reservoirs were initially projected using occurrence data of 375 occurrences for *Cricetomys gambianus*, 715 occurrences for *Funisciurus* spp., 47 occurrences for *Graphiurus crassicaudatus*, and 88 occurrences for *Graphiurus lorraineu* in Africa. The average area under curve (AUC) for testing dataset for all generated models ranged from 0.88 for *Graphiurus lorraineu* to 0.96 for *Graphiurus crassicaudatus*. These three animals, *Cricetomys gambianus*, *Graphiurus lorraineus*, and *Graphiurus crassicaudatus*, were included in the model construction, but *Funisciurus* spp. were excluded when the multicollinearity was considered (Fig. 3, details in Figure S5-S13).

After removing human-to-human transmission and polygons of zoonotic transmission occurrence with an area larger than 1° × 1° (about 110 km × 110 km at the equator), a total of 547 occurrences were retained for geospatial modelling to mimic zoonotic transmission of MPXV (detailed in Table S19). Seven variables, including three animal-related variables (predicted distributions of *Graphiurus lorraineus* and *Graphiurus crassicaudatus*, and richness of Rodentia) and four ecoclimatic indicators (annual precipitation, min temperature of coldest month, isothermality, and annual mean temperature) after the bootstrapping procedure. The model yielded an average AUC of 0.95 with 95% CI ranging from 0.94 to 0.97 (Table S20) for the 100 sub-models, demonstrating a good capacity in predicting regions with environmental suitability for MPXV occurrence. Based on the model, annual precipitation and minimal temperature of the coldest month per year were determined as the significant predictors, with a relative contribution (RC) ≥ 20.00% in the ensembled models, followed by isothermality with an RC of 9.27% (95% CI: 7.88–10.67). Other four variables (*Graphiurus lorraineus*, richness of Rodentia, annual mean temperature, *Graphiurus crassicaudatus*) had an RC ranging from 6.51% to 8.02% (details in Table S20). The partial dependence plots illustrated the associations between these explanatory variables and MPXV zoonotic niche (Figure S14).

**Fig. 4:**
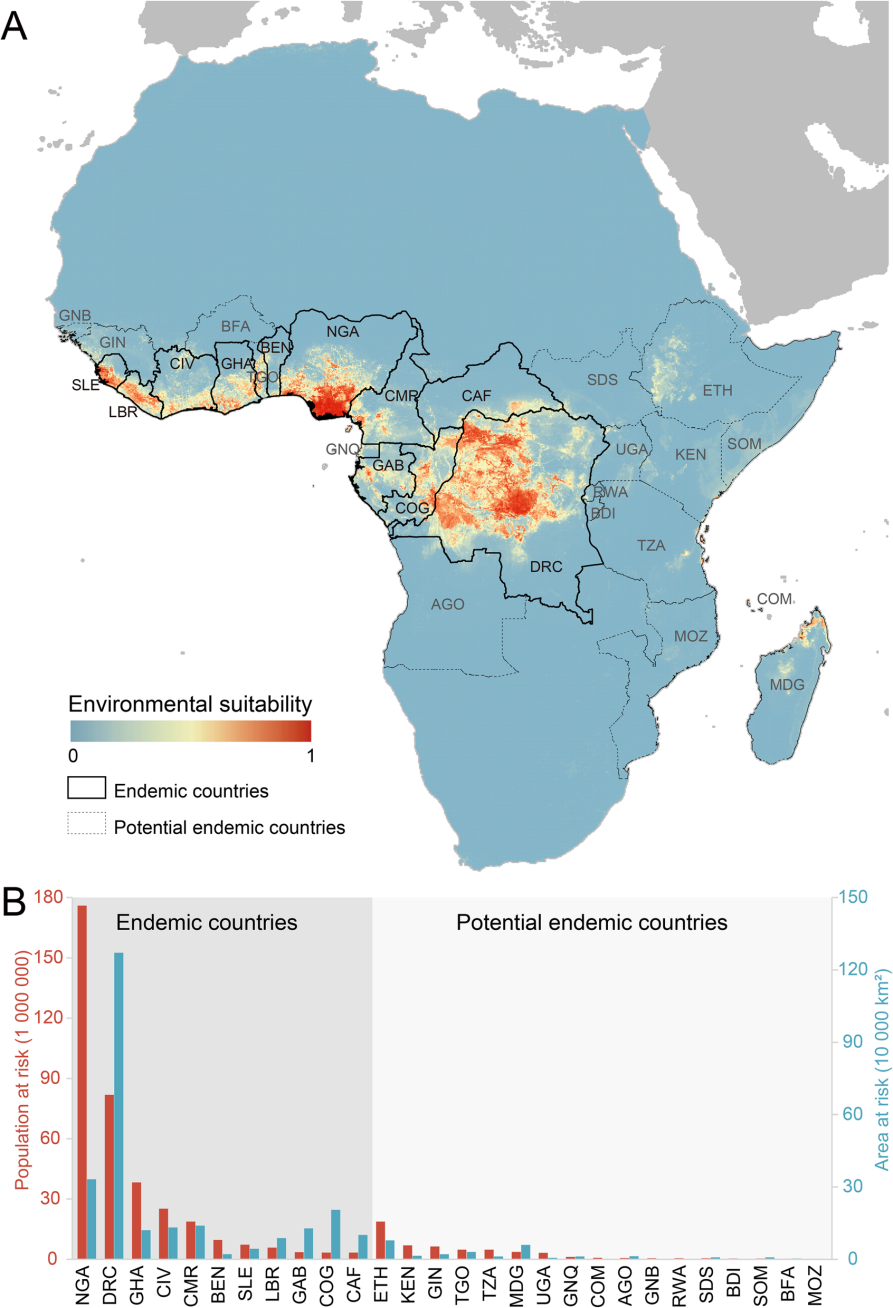
Model-predicted distribution of zoonotic niche of MPXV occurrence and risk assessment in Africa. **A** The geographical distribution of model-predicted zoonotic niche of MPXV occurrence. Countries with MPXV detected positively from humans or animals are outlined by solid line, while others predicted with risk of MPXV occurrence but still without MPXV reporting are outlined by dotted line; **B** The population and area at model-predicted risk of zoonotic transmission for endemic and potential endemic countries. The country abbreviations were listed with full name in Table S26

In addition to the 11 countries that have reported human cases, 17 countries in Africa have a model-predicted high-risk of MPXV occurrence (Fig. 4A). Nigeria has the largest population size at risk of MPXV infections (176.00 million), followed by DRC (with 81.88 million at-risk population) and Côte d'Ivoire (38.30 million). Eight countries were predicted to fall within high-risk areas (> 100 thousand km^2^), with DRC having the widest areas (1 271.12 thousand km^2^), followed by Nigeria (332.44 thousand km^2^) and Congo (205.04 thousand km^2^). Ethiopia has the greatest population size at probable predicted risk of MPXV zoonotic transmission among these potential endemic countries (18.81 million), followed by Kenya (6.92 million) and Guinea (6.37 million). Similarly, Ethiopia has the high-risk areas predicted by the model (78.69 thousand km^2^), followed by Madagascar (59.44 thousand km^2^) and Togo (30.76 thousand km^2^).

The majority of the predicted high-risk areas were within Western Africa and Congo Basin (Fig. 4B, Table S21). In addition to Africa, northern part of South America, the Caribbean States, and several regions in Southeast and South Asia as well as Western Pacific Region were predicted by BRT model as highly suitable for the MPXV occurrence once the virus invades (Figure S15). Brazil, Indonesia, and Colombia have the biggest territory with a MPXV-suitable environment, whereas Indonesia, India and the Philippines have the largest population size at risk of MPXV infection (Table S22 and S23).

### Interregional transmission risk of MPXV by airlines

**Fig. 5:**
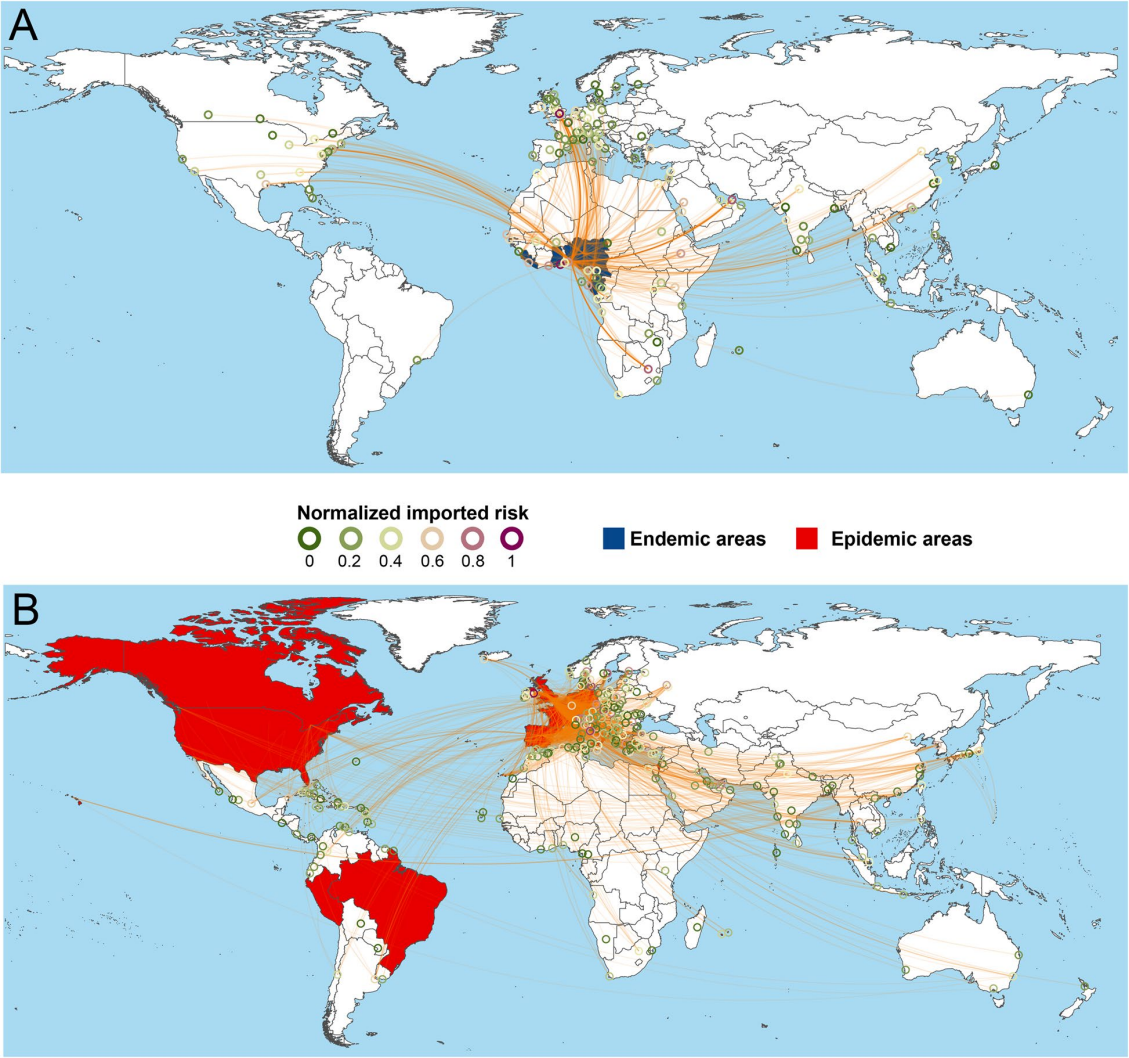
Interregional transmission risk of MPXV. **A** imported risk from endemic countries; **B** imported risk from current epidemic countries in 2022. The endemic and main epidemic countries were indicated by the background of brown and orange color. The orange curves represent air routes from cities in MPXV endemic countries or main epidemic countries to other cities around the world, and its opacity represents the magnitude of the imported risk transmitted by air routes. The imported risks outside endemic countries are also indicated by color of circles (from green to purple). Air routes with the number of passengers less than 500 persons from endemic countries or less than 150 000 persons from current epidemic countries per year are not displayed

Among all the endemic countries, Nigeria, Ghana, and Côte d'Ivoire had the highest odds of exporting MPXV cases, followed by the Cameroon, DRC, Congo, and Gabon (Fig. 5 and Table S24). Western Europe was at risk of case importing from endemic countries, with the highest risk observed in the southern United Kingdom, the northern France, Italy, Germany, and Belgium, where travel-related outbreaks and MPXV transmission from humans to companion animals and pets also exist. Based on viral clade-specific analysis, the travel-related infections in the West African clade originated mainly from Nigeria. Travel-related infections with MPXV of Congo Basin clade originated mainly from Ghana, Côte d'Ivoire, and DRC, and spread to France and the United Kingdom (Figure S16).

The estimated highest MPXV exported-risk countries were the United Kingdom, Spain, and Germany (Table S25). The top 15 predicted imported countries were located in Europe (11 countries, e.g., Italy, Ireland and Poland), Asian (2 countries, including Turkey and China), North America (Mexico) and Africa (Morocco) (Figure S16).

## Discussion

By combining spatial–temporal and phylogenetic data on MPXV, we mapped the global distribution of MPXV infections in humans and animals, defining populations at risk from zoonotic transmission or travel-related infections, thereby providing a robust framework for assessing its global impact. Since its introduction in 1970, a total of 49 432 human cases of MPXV infections have been reported, primarily in Central Africa and Western Africa until 2022, with occasional travel-related cases in other countries and an animal trade-related outbreak in the United States in 2003. The current outbreaks associated with the West African clade have milder disease severity and resulted in less fatalities, however, remains as a serious concern when considering the marginally increased R_t_ from 2000–2009 to the most recent decade, indicating an elevated transmissibility. The international connectivity by airline traffic posed another severe concern, many Europe countries especially those in West Europe have a high risk of travel-related infections both from endemic and current epidemic countries, and three cities (Chongqing, Beijing and Guangzhou) in China have reported five MPXV cases [26], indicating that more attention should be paid to the spillover and human-to-human transmission, and if it continuously spreads, it will be a shock to the vulnerable global health system during the COVID-19 pandemic [27].

BRT models reveal that ecological niche of MPXV was significantly associated with four ecoclimatic factors, such as annual precipitation, min temperature of coldest month, isothermality, and annual mean temperature, as well as three reservoirs-related factors, including the predicted distributions of *Graphiurus lorraineus* and *Graphiurus crassicaudatus*, and the richness of Rodentia. Ecoclimatic factors may lead to an increase in MPXV infections in humans from zoonotic transmission. Precipitation can affect the behavior and movements of reservoir hosts of MPXV. For example, heavy rainfall can lead to changes in the distribution of water sources, affecting the movement patterns of rodents and their interactions with each other and other animals (e.g., non-human primates) [28]. The min temperature of the coldest month, isothermality, and annual mean temperature may affect the activity and reproduction of reservoir hosts, the survival and transmission of the virus, and the overall risk of disease outbreaks [29]. This climatic-driven zoonotic spillover potentially generated by human activities such as deforestation, combined with agricultural activities and hunting in forests, may contribute to the spread of monkeypox in endemic areas [30].

Ecological drivers are part of the complex interrelationship between humans, animals, and the environment during the zoonotic transmission of MPXV. Four rodent reservoirs were used to build the model, two of which (*Graphiurus lorraineus* and *Graphiurus crassicaudatus*) were included in the final model, revealing a highly correlation with the zoonotic niche of MPXV. The negative trend between rodent richness and MPXV ecological niche suggests that the dilution effect may apply to the MPXV distribution [31], as a high richness environment may reduce the activity of reservoir rodents, and consequently reduce the ecological niche of MPXV, which has also been observed in Lassa fever [32]. It is important to note that the inclusion of these rodent species and the overall richness of Rodentia in the analysis highlights the importance of studying rodents and their role in zoonotic diseases. Our study indicates that researches about rodents should not be ignored due to the vital roles of rodents in many zoonotic diseases, e.g., Lassa fever and human infection with hantaviruses that have persistent threats to global health [33, 34]. There is still a hypothetical risk of human-to-animal transmission, and it is worth focusing on those areas outside Africa where our model projects highly suitable environments for MPXV occurrence, avoiding the colonization of the virus in animals. Rodentia animals have been intensively reported to harbor MPXV, our study highlights the association between African dormice (*Graphiurus* spp.) and MPXV niche, and other studies have revealed that rope squirrel (*Funisciurus* spp.) showed the overlap niche with MPXV [35]. These results contribute to natural advantages in viral connectivity or horizontal transmission within reservoir rodents, promoting virus sharing and dissemination among these hosts [36]. Collectively, these insights contribute to our understanding of the complex ecology of MPXV transmission dynamics.

Our model predicted potential ecological niche of the MPXV throughout the Congo Basin and Western Africa, including parts of the DRC, Nigeria, Congo, Côte d'Ivoire, and some areas without reported cases, e.g., Ethiopia, Kenya, and Guinea. Some areas are lack of efficient surveillance network of MPXV infection in animals or clinical patients, in where under-reporting due to limited field investigations or laboratory identification might contribute to its continuous transmission among humans, until outbreaks were noticed [37]. The international community could consider to allocate resources to strengthen surveillance, laboratory testing, case investigation and contact tracing, clinical management, vaccinations and immunization, and risk communication in these countries.

The unprecedented and unexpected outbreaks outside Africa since May 2022, have attracted the focus of global public health systems and have been issued as a PHEIC by WHO [6, 38]. Based on our updated estimates of the transmissibility, the effective reproduction number in European countries (e.g., Germany, France, and Switzerland) since May 2022 is greater than 1, indicating a probable increase in its transmissibility. Countries experiencing higher R_t_ values need to consider implementing or intensifying targeted interventions, such as increased surveillance and case detection, contact tracing, isolation and quarantine measures, public awareness campaigns, and vaccination programs where available. In addition, monitoring changes in R_t_ can reveal the impact of specific interventions and policy implementations, allowing for timely adjustments of control strategies as the outbreak evolves [39, 40]. For countries at a higher-imported risk, e.g., the United Kingdom [3, 41], the United States [41], South Africa [42], and Saudi Arabia [43], measures to increase the sensitivity of case detection are helpful for the control of the disease. As global routine measures of prevention and control of the COVID-19 pandemic were made, the demand for gatherings is increasing day by day. Although the role of MSM transmission in the spread of monkeypox cannot be ruled out at this time [14, 44–47], clustered cases may occur in any group that has been in close contact in a large-scale gathering event. Continuous outbreaks of 2022-monkeypox have revealed significant gaps in understanding the mechanisms of viral transmission and the continuously changing epidemiological characteristics of the disease, and a more integrated approach to epidemic preparedness is long overdue.

The study is subject to several limitations. First, the epidemic status of monkeypox in Africa may be underreported due to weak medical and health conditions in some countries. Second, although smallpox had been eliminated in the world and the smallpox vaccination had been stopped in the 1980s [48], good cross immunizations between smallpox and MPXV have been reported, we did not consider the susceptibility of populations to monkeypox in the analysis of inter-regional transmission of MPXV, which might overestimate the risk of interregional transmission.

## Conclusions

In conclusion, our study provides a comprehensive assessment of the potential zoonotic niche and imported risk of MPXV. Targeted public health surveillance and control measures are necessary to combat these outbreaks. We call for continuously and enhanced active surveillance of MPXV infections in humans and animals as well as the dynamics of its adaptation to humans in its potential endemic areas and current epidemic areas. Studies involving mechanisms of MPXV origin, host range, and spillover events, as well as the prediction of epidemic trends are urgently needed.

### Supplementary Information

**Additional file 1: Text S1.** Phylodynamic analyses of MPXV. **Text S2.** The procedure of estimation of adequate reproductive number (R_t_). **Text S3.** BRT modeling analyses. **Text S4.** The risk of international spread for MPXV and metapopulation model. **Text S5.** Demographic characteristics and spatiotemporal changes of MPXV infections. **Table S1.** Literature search syntax of monkeypox. **Table S2.** Information of MPXV infections from grey literature. **Table S3.** Guideline for inclusion and exclusion of literature of MPXV infections. **Table S4.** Data extracted for literature included in the study. **Table S5.** Criteria of human and animal infections with MPXV. **Table S6.** Genome sequences used in the phylogenetic analyses. **Table S7.** Source used to analysis the effective reproductive number for MPXV from 1990 to 2020. **Table S8.** Definition of MPXV infection in potential reservoir hosts. **Table S9.** Information of data source in the modeling analysis. **Table S10.** Description of 45 potential influencing factors used in the modelling efforts. **Table S11.** Ecological factors potentially associated with the MPXV zoonotic transmission used in the modelling analysis. **Table S12.** Number and resource of occurrence data for animals extracted from Global Biodiversity Information Facility (GBIF). **Table S13.** Source of data to describe the yearly case number in infected regions. **Table S14.** Source of data to describe the monthly case number in infected regions. **Table S15.** Epidemiological features of global human monkeypox patients recorded in 2022. **Table S16.** Case numbers of MPXV infections in humans by country and year. **Table S17.** Cases of MPXV infections in animals by country and year. **Table S18.** Locations of detection of MPXV in animals with transmission to humans. **Table S19.** Locations with MPXV infection in humans and animals used to build BRT models. **Table S20.** Mean (95% percentiles) relative contributions of variables (RC≥3%) and mean AUCs (95% percentiles) of five BRT models in this study. **Table S21.** Model-Predicted populations and areas at risk of MPXV infections in Africa. **Table S22.** Top 10 countries with suitable habitats for MPXV (ranked by population). **Table S23.** Top 10 countries with suitable habitats for MPXV (ranked by area). **Table S24.** The imported risk of MPXV caused by flight from endemic countries. **Table S25.** The imported risk of MPXV caused by flight from epidemic countries. **Table S26.** Country or region abbreviations used throughout the article. **Figure S1.** Timeline of MPXV cases from 1970 to 2022. **Figure S2.** The number of MPXV human infections by periods and countries. **Figure S3.** The locations of reported MPXV infections in animals and zoonotic transmission to human. **Figure S4.** The effective reproduction number (R_t_) of monkeypox in human-to-human transmission. **Figure S5.** Correlation matrix of variables for *Cricetomys gambianus*. **Figure S6.** Correlation matrix of variables for *Funisciurus* spp. **Figure S7.** Correlation matrix of variables for *Graphiurus crassicaudatus*. **Figure S8.** Correlation matrix of variables for *Graphiurus lorraineus*. **Figure S9.** Correlation matrix of variables for MPXV. **Figure S10.** The relative contribution and response curve of BRT models for *Cricetomys gambianus*. **Figure S11.** The relative contribution and response curve of BRT models for *Funisciurus* spp. **Figure S12.** The relative contribution and response curve of BRT models for *Graphiurus crassicaudatus*. **Figure S13.** The relative contribution and response curve of BRT models for *Graphiurus lorraineus*. **Figure S14**. The relative contribution and response curve of BRT models for MPXV. **Figure S15.** Global environmental suitability of MPXV. **Figure S16.** The chord graph of interregional risk of MPXV from endemic countries (A) and main current epidemic countries (B) caused by flight. **Text S6.** Reference.

## Data Availability

All relevant data are within the paper and its Supplementary data.

## Abbreviations

MPXV: Monkeypox virus
R_t_: The effective reproductive number
WHO: The World Health Organization
PHEIC: Public Health Emergency of International Concern
WOS: ISI Web of Science
GIDEON: Global Infectious Diseases and Epidemiology Network
CDC: USA Center for disease control and prevention
NCDC: Nigeria Centre for Disease Control and Prevention
GBIF: Global Biodiversity Information Facility
IUCN: International Union for Conservation of Nature
BRT: Boosted Regression Tree
ROC: Receiver Operating Characteristic Curve
OD: Origin–destination
MSM: Men who have sex with men
DRC: Democratic Republic of the Congo
AUC: Area Under Curve
RC: Relative Contribution
COVID-19: Coronavirus disease 2019
