# Supplementary material for: Mapping global zoonotic niche and interregional transmission risk of monkeypox: a retrospective observational study
Source: Global Health. 2023 Aug 17;19:58. doi: 10.1186/s12992-023-00959-0 (PMC10436417; doi:10.1186/s12992-023-00959-0)
Supplement: Supplementary file 1 — Additional file 1: Text S1. Phylodynamic analyses of MPXV. Text S2. The procedure of estimation of adequate reproductive number (Rt). Text S3. BRT modeling analyses. Text S4. The risk of international spread for MPXV and metapopulation model. Text S5. Demographic characteristics and spatiotemporal changes of MPXV infections. Table S1. Literature search syntax of monkeypox. Table S2. Information of MPXV infections from grey literature. Table S3. Guideline for inclusion and exclusion of literature of MPXV infections. Table S4. Data extracted for literature included in the study. Table S5. Criteria of human and animal infections with MPXV. Table S6. Genome sequences used in the phylogenetic analyses. Table S7. Source used to analysis the effective reproductive number for MPXV from 1990 to 2020. Table S8. Definition of MPXV infection in potential reservoir hosts. Table S9. Information of data source in the modeling analysis. Table S10. Description of 45 potential influencing factors used in the modelling efforts. Table S11. Ecological factors potentially associated with the MPXV zoonotic transmission used in the modelling analysis. Table S12. Number and resource of occurrence data for animals extracted from Global Biodiversity Information Facility (GBIF). Table S13. Source of data to describe the yearly case number in infected regions. Table S14. Source of data to describe the monthly case number in infected regions. Table S15. Epidemiological features of global human monkeypox patients recorded in 2022. Table S16. Case numbers of MPXV infections in humans by country and year. Table S17. Cases of MPXV infections in animals by country and year. Table S18. Locations of detection of MPXV in animals with transmission to humans. Table S19. Locations with MPXV infection in humans and animals used to build BRT models. Table S20. Mean (95% percentiles) relative contributions of variables (RC≥3%) and mean AUCs (95% percentiles) of five BRT models in this study. Table S21. Model- [file 12992_2023_959_MOESM1_ESM.docx]

**Supplementary appendix**

Supplement to: Mapping global zoonotic niche and interregional transmission risk of monkeypox: a retrospective observational study

**Table of Contents**

[Appendix Text S1: Phylodynamic analyses of MPXV 3](#_Toc141368567)

[Appendix Text S2: The procedure of estimation of adequate reproductive number (R_t_) 4](#_Toc141368568)

[Appendix Text S3: BRT modeling analyses 6](#_Toc141368569)

[Appendix Text S4: The risk of international spread for MPXV and metapopulation model 8](#_Toc141368570)

[Appendix Text S5: Demographic characteristics and spatiotemporal changes of MPXV infections 10](#_Toc141368571)

[Appendix Table S1: Literature search syntax of monkeypox 11](#_Toc141368572)

[Appendix Table S2: Information of MPXV infections from grey literature 12](#_Toc141368573)

[Appendix Table S3: Guideline for inclusion and exclusion of literature of MPXV infections 15](#_Toc141368574)

[Appendix Table S4: Data extracted for literature included in the study 16](#_Toc141368575)

[Appendix Table S5: Criteria of human and animal infections with MPXV 17](#_Toc141368576)

[Appendix Table S6: Genome sequences used in the phylogenetic analyses 18](#_Toc141368577)

[Appendix Table S7: Source used to analysis the effective reproductive number for MPXV from 1990 to 2020 30](#_Toc141368578)

[Appendix Table S8: Definition of MPXV infection in potential reservoir hosts 31](#_Toc141368579)

[Appendix Table S9: Information of data source in the modeling analysis 32](#_Toc141368580)

[Appendix Table S10: Description of 45 potential influencing factors used in the modelling efforts 33](#_Toc141368581)

[Appendix Table S11: Ecological factors potentially associated with the MPXV zoonotic transmission used in the modelling analysis 34](#_Toc141368582)

[Appendix Table S12: Number and resource of occurrence data for animals extracted from Global Biodiversity Information Facility (GBIF) 40](#_Toc141368583)

[Appendix Table S13: Source of data to describe the yearly case number in infected regions 41](#_Toc141368584)

[Appendix Table S14: Source of data to describe the monthly case number in infected regions 43](#_Toc141368585)

[Appendix Table S15: Epidemiological features of global human monkeypox patients recorded in 2022 44](#_Toc141368586)

[Appendix Table S16: Case numbers of MPXV infections in humans by country and year 45](#_Toc141368587)

[Appendix Table S17: Cases of MPXV infections in animals by country and year 48](#_Toc141368588)

[Appendix Table S18: Locations of detection of MPXV in animals with transmission to humans 49](#_Toc141368589)

[Appendix Table S19: Locations with MPXV infection in humans and animals used to build BRT models 50](#_Toc141368590)

[Appendix Table S20: Mean (95% percentiles) relative contributions of variables (RC≥3%) and mean AUCs (95% percentiles) of five BRT models in this study 58](#_Toc141368591)

[Appendix Table S21: Model-Predicted populations and areas at risk of MPXV infections in Africa 59](#_Toc141368592)

[Appendix Table S22: Top 10 countries with suitable habitats for MPXV (ranked by population) 60](#_Toc141368593)

[Appendix Table S23: Top 10 countries with suitable habitats for MPXV (ranked by area) 61](#_Toc141368594)

[Appendix Table S24: The imported risk of MPXV caused by flight from endemic countries 62](#_Toc141368595)

[Appendix Table S25: The imported risk of MPXV caused by flight from epidemic countries 63](#_Toc141368596)

[Appendix Table S26: Country or region abbreviations used throughout the article 64](#_Toc141368597)

[Appendix Figure S1: Timeline of MPXV cases from 1970 to 2022. 65](#_Toc141368598)

[Appendix Figure S2: The number of MPXV human infections by periods and countries. 66](#_Toc141368599)

[Appendix Figure S3: The locations of reported MPXV infections in animals and zoonotic transmission to human. 67](#_Toc141368600)

[Appendix Figure S4: The effective reproduction number (Rt) of monkeypox in human-to-human transmission. 68](#_Toc141368601)

[Appendix Figure S5: Correlation matrix of variables for *Cricetomys gambianus*. 69](#_Toc141368602)

[Appendix Figure S6: Correlation matrix of variables for *Funisciurus* spp. 70](#_Toc141368603)

[Appendix Figure S7: Correlation matrix of variables for *Graphiurus crassicaudatus*. 71](#_Toc141368604)

[Appendix Figure S8: Correlation matrix of variables for *Graphiurus lorraineus*. 72](#_Toc141368605)

[Appendix Figure S9: Correlation matrix of variables for MPXV. 73](#_Toc141368606)

[Appendix Figure S10: The relative contribution and response curve of BRT models for *Cricetomys gambianus*. 74](#_Toc141368607)

[Appendix Figure S11: The relative contribution and response curve of BRT models for *Funisciurus* spp. 75](#_Toc141368608)

[Appendix Figure S12: The relative contribution and response curve of BRT models for *Graphiurus crassicaudatus*. 76](#_Toc141368609)

[Appendix Figure S13: The relative contribution and response curve of BRT models for *Graphiurus lorraineus*. 77](#_Toc141368610)

[Appendix Figure S14: The relative contribution and response curve of BRT models for MPXV 78](#_Toc141368611)

[Appendix Figure S15: Global environmental suitability of MPXV 79](#_Toc141368612)

[Appendix Figure S16: The chord graph of interregional risk of MPXV from endemic countries (A) and main current epidemic countries (B) caused by flight 80](#_Toc141368613)

[Appendix Text S6: Reference 81](#_Toc141368614)

# Appendix Text S1: Phylodynamic analyses of MPXV

In order to construct the phylogenomic tree of monkeypox virus (MPXV) genomes, we conducted a meticulous curation process to select representative genomes that played a significant role in enhancing our understanding of MPXV diversity. We carefully considered the relevance and contribution of each genome to ensure a comprehensive representation. For the nucleotide phylogenetic analysis, we incorporated additional contextual data, including chronological and geographical information associated with the genome records. By integrating this valuable information, we were able to gain deeper insights into the evolutionary relationships among the MPXV genomes. This comprehensive approach allowed us to capture the genetic variations and evolutionary history of the virus, providing a more accurate depiction of its diversification patterns and helping us understand the factors that have shaped its evolution over time. The phylodynamic analyses were carried out according to the following scheme:

**Dataset of MPXV genomes**

We conducted a comprehensive analysis on a dataset consisting of 525 high-quality MPXV genomes. These genomes were obtained from the Viral NCBI database and represented a substantial collection of genetic information. The genomes were sequenced over a span of years, from 1958 to July 22, 2022, as detailed in Table S6. To ensure consistency and comparability, the obtained sequences were subjected to trimming and alignment procedures. Specifically, the trimmed sequences were aligned to a reference genome with Accession numbers NC_003310 and NC_063383. This alignment step helped to standardize the data and ensure accurate comparisons of the sequences. In addition to the genomic data, we also extracted temporal and geographic information associated with each genome from the GenBank.

**Analysis of the nucleotide sequences**

We employed the powerful multiple sequence alignment tool MAFFT (FFT-NS-2, v. 7.450) [1]. This allowed us to accurately align the sequences and highlight their similarities and differences. To begin, we inputted the nucleotide sequences of the MPXV genomes into the MAFFT tool. The tool then applied the FFT-NS-2 alignment method, which employs progressive pairwise alignment and iterative refinement processes to align the sequences.

**Phylogenetic tree of MPXV**

To generate the phylogenetic tree, we employed the maximum-likelihood method. Specifically, we utilized the maximum-likelihood approach with 1000 bootstrap replicates. To perform this analysis, we used the RaxML web server [2]. Each bootstrap replicate involved randomly selecting a subset of sequences with replacement from the original dataset, and the maximum-likelihood analysis was performed on each replication. The results of these replicates were then combined to construct a consensus phylogenetic tree. The incorporation of 1000 bootstrap replicates provided a reliable estimation of the branch support values and improved the confidence in the inferred evolutionary relationships portrayed by the phylogenetic tree.

To enhance the visual representation of the phylogenetic tree, we employed the user-friendly and versatile Figtree software (http://tree.bio.ed.ac.uk/software/figtree). Once we obtained the phylogenetic tree from our analysis, we imported the tree file into Figtree for further visualization and refinement. Using Figtree, we were able to modify the tree layout, adjust the branch lengths, and assign colors and labels to the different clades, subclades, or groups within the tree.

**Supplementary reference:**

1. Katoh K, Standley D M. MAFFT multiple sequence alignment software version 7: improvements in performance and usability. *Molecular biology and evolution*. 2013; 30(4): 772-780.

2. Stamatakis A. RAxML version 8: a tool for phylogenetic analysis and post-analysis of large phylogenies. *Bioinformatics*. 2014; 30(9): 1312-1313.

# Appendix Text S2: The procedure of estimation of adequate reproductive number (R_t_)

We used two methods to calculate the effective reproduction number (R_t_) of monkeypox in 1990-2020 and in 2022 respectively.

The R_t_ for monkeypox was projected from 1990 to 2020 using a method for subcritical outbreaks of infectious diseases comprising both zoonotic (reservoir-to-human) and human-to-human transmission. The term "chain of transmission" refers to a sequence of events starting with a zoonotic transmission, followed by subsequent human-to-human transmissions, where R_t_ in human populations is below 1, a proportion of cases can be linked to exposure to the animal reservoir [1].

Based on Lloyd-Smith et al.'s work [2], the length of a transmission chain is denoted as L. The offspring distribution, representing the number of persons infected by a single case, follows a negative binomial distribution with mean *Rt* and overdispersion parameter k (characterizing case-to-case variation in infectiousness). The probability that a chain is of length L is given by [3]:

$$g(L|R_{t},k)=\frac{\Gamma\left( \left( k+1 \right)L-1 \right)k^{kL+1}R_{t}^{L-1}}{\Gamma(kL+1)\Gamma(L){(R_{t}+k)}^{\left( k+1 \right)L-1}}$$

In a typical transmission chain with an average length of $\bar{L}$ , there is one reservoir-to-human transmission event and $(\bar{L}-1)$ subsequent human-to-human infections. The probability G of a case being infected by the reservoir, when randomly picked up by surveillance, is given by$G=1/\bar{L}$. However, for subcritical outbreaks (0<*R_t_*<1), branching process theory reveals that the average chain length is $\bar{L}=1/(1-R_{t})$, resulting in $G=1-R_{t}$. To estimate the upper limit of *R_t_* when the detection of a case doesn't influence other cases in the same cluster, we use $R_{t}=1-G$, where G is the proportion of detected cases that are infected by the reservoir. This is a general result that is independent of the case detection rate ρ and the overdispersion parameter k and that does not require data on clusters. It is valid as long as outbreaks are subcritical (0<*R_t_*<1).

In our study, we employed the proportion of cases infected by the natural reservoir among detected cases (G) to estimate upper *R_t_*. As indicated before, upper *R_t_* was calculated as one minus the fraction of cases infected by the reservoir out of the total number of identified cases. Using the counts of all reported cases and patients infected via reservoirs, we determined upper *Rt* and its 95% confidence interval for monkeypox outbreaks between 1990 and 2020, as presented in Table S7.

To estimate *R_t_* in 2022 monkeypox outbreak, we simply use the estimate_R function in the R package “EpiEstim” [4]. This method relies on a branching process model, which assumes that the incidence of new cases on day t(*It*) can be represented by a Poisson process:

$$I_{t}\sim Pois(R_{t}\sum_{s=1}^{t} I_{t-s}\omega_{s})$$

where *R_t_* is the time-varying reproduction number (i.e., the average number of cases caused by a primary case infected at time *t*, assuming that conditions remain the same after time *t*), and the past incidence (*I_t-s_*) is weighted by $\omega_{s}$, the probability mass function of the generation time (the time between infection in a case and their infector). In practice, as infection itself is difficult to observe, the incidence of symptomatic cases can be used instead and $\omega_{s}$, can be approximated by the serial interval (SI, the time between symptom onset in a case and their infector) [5]. Cori et al. developed a method to estimate *R_t_* from the renewal equation that is suitable for real-time application. The method is implemented in the R package “EpiEstim”, where estimation is performed over user-defined time windows within which R_t_ is assumed constant [6].

On the basis of a Bayesian approach that depends on the time series and the distribution of serial interval, the R_t_ of current monkeypox outbreaks in 2022 was predicted using confirmed cases from May 6 to July 22 in the global.health database. R_t_ is estimated within a Bayesian framework, using a Gamma distributed prior. In the modeling analysis, we assumed a mean and standard deviation of the serial interval of 9.7 and 2.7 days, respectively [7].

**Supplementary reference:**

1. Cauchemez S, Epperson S, Biggerstaff M, Swerdlow D, Finelli L, Ferguson NM. Using routine surveillance data to estimate the epidemic potential of emerging zoonoses: application to the emergence of US swine origin influenza A H3N2v virus. *PLoS Med*. 2013; 10(3): e1001399.

2. Lloyd-Smith JO, Schreiber SJ, Kopp PE, Getz WM. Superspreading and the effect of individual variation on disease emergence. *Nature*. 2005; 438(7066):355-359.

3. Nishiura H, Yan P, Sleeman CK, Mode CJ. Estimating the transmission potential of supercritical processes based on the final size distribution of minor outbreaks. *J Theor Biol*. 2012; 294: 48-55.

4. Cori A, Cauchemez S, Ferguson N M, et al. Package “EpiEstim”. CRAN: Vienna Austria, 2020.

5. Nash RK, Nouvellet P, Cori A. Real-time estimation of the epidemic reproduction number: Scoping review of the applications and challenges. *PLOS Digit Health*. 2022; 1(6): e0000052.

6. Cori A, Ferguson NM, Fraser C, Cauchemez S. A new framework and software to estimate time-varying reproduction numbers during epidemics. *Am J Epidemiol*. 2013; 178(9): 1505-1512.

7. Nolen LD, Osadebe L, Katomba J, Likofata J, Mukadi D, Monroe B, et al. Extended human-to-human transmission during a Monkeypox outbreak in the Democratic Republic of the Congo. *Emerg Infect Dis*. 2016; 22(6): 1014-1021.

# Appendix Text S3: BRT modeling analyses

**Covariates assembled and used in the analyses**

Ecological variables were selected for inclusion in this study because previous work hypothesized that environmental risk factors or the animal factors alone cannot explain the emergence of monkeypox outbreak in Africa [1-3], on the other hand, human activity have greatly increased the interactions between humans and animals [4]. In our study, a suite of ecologically relevant gridded environmental covariates for Africa was compiled to capture the intricate interplay between the environment, animal reservoirs, and human activity in driving the dynamics of MPXV transmission, including data at the spatial layers for 39 explanatory variables that were grouped into three categories, i.e., environmental-related, animal-related, and human activity associated factors. Of them, 31 environmental related predictors were obtained: (1) 19 bioclimatic variables (also called BIO 01−19); (2) leaf area index; (3) elevation; (4) latitude; (5) the land cover CCI climate research data package (CRDP) including nine land cover variables other than cropland and urban construction land. Nine human activity related variables were obtained: (1) two land cover variables in CRDP (cropland and urban construction land); (2) population size; (3) global downscaled GDP and (4) human footprint index. Three animal-related variables (richness of mammal, Rodentia and Primates) were also obtained. Covariates provided with a finer resolution than the study grid (10 km×10 km) were resampled and averaged to match the desired resolution.

**Implicated animal reservoir distributions**

Over recent years, Rodentia had been intensively reported to harbor MPXV. We build the ecological niche models for four hypothesized to be monkeypox potential reservoir hosts: rope squirrels (*Funisciurus* spp. species, family Scuridae), African dormice (two species: *Graphiurus crassicaudatus* and *Graphiurus* *lorraineus*, family Gliridae), and giant pouched rats (*Cricetomys gambianus*, family Nesomyidae). These specific taxa were chosen based on molecular tests confirming their infections with MPXV (see Appendix Table S8). Additionally, they represented both arboreal rodents (rope squirrels) and terrestrial rodents (African dormice and giant pouched rats), which are known to have different types of interactions with humans. [5]. To fully reflect contribution of animals in the model of MPXV transmission, we at the first step developed predictive maps for distribution of four types of animals, which had been implicated as potential reservoirs of the disease: *Funisciurus* spp.*, Graphiurus crassicaudatus, Graphiurus lorraineus* and *Cricetomys gambianus*. While these animals are included in the dataset of mammal/Rodentia richness, there is some disagreement with independently-sourced occurrence data. As a result, a predictive modelling approach was used to create a continuous surface of habitat suitability for these species which we then included as a predictor in the model. Data on the global occurrence for all these animals were extracted from Global Biodiversity Information Facility（GBIF）(https://www.gbif.org) and then the spatial range of *Graphiurus crassicaudatus*, *Graphiurus lorraineus* and *Cricetomys gambianus* determined by the IUCN red list (https://www.iucnredlist.org/) of threatened species were used to modify the occurrence. Given the misclassification of *Cricetomys gambianus* as another species, *Cricetomys ansorgei*, the data from GBIF was modified by spatial range determined by IUCN red list of Threatened Species (i.e. points outside this spatial range determined by IUCN were deleted from animal database).[6, 7] When modeling for these animals, environmental data and covariates of land cover were chosen. As a result, the final predicted distribution of these four types of animals, as well as other 39 variables we collected, were included in the final models to explore the ecological niche of MPXV.

**MPXV distribution modelling**

We created a global grid-map of 10 km×10 km used ArcGIS 10.7 (Esri Inc, Redlands, CA, USA) and then associated with explanatory variables. All location data were geopositioned using Google Map as precisely as possible. For the locations which precise geographic information are available, the latitude and longitude were directly included, while for those with precise geographic information unavailable, a polygon was defined to cover the reported region. All data that had been geopositioned were checked to ensure coordinates were plausible and then occurrences were standardized to remove duplicates, so that each individual record used in our model represented an occurrence of MPXV infections. Then each occurrence data was matched to the grid-map according to its coordinate and only one presence grid was counted if multiple records were associated with the same grid. To reduce multicollinearity among the features for ecological modeling, we evaluated pairwise Pearson correlations. For each pair of features with an absolute Pearson correlation coefficient higher than 0.70, the one with a higher average correlation with all other features was excluded from analyses. Each occurrence data was matched to the grid-map according to its coordinate and only one presence grid was counted if multiple records were associated with the same grid.

A boosted regression tree (BRT) using the ‘gbm.step’ function in the R package “dismo” was used to build the relationship between MPXV occurrence and environmental, ecoclimatic and biological variables [8]. A case-control study design was conducted to build the ecological model. Briefly, occurrences within human infections with zoonotic transmission and animal infections were considered as “cases”, and those occurrences of MPXV where polygon areas were greater than one square degree (about 110 km×110 km in areas at the equator) were excluded from the input of the modeling analysis to avoid the introduction of an additional bias. We also excluded the occurrence of person-to-person transmission. Pseudo-absence data (“controls”) was chosen randomly within a range of 20–1000 km around the presence grids, and the number of pseudo-absences locations is usually three times number of presence data. A tree complexity of five, a learning rate of 0.005 and a bagging fraction of 75% were used based on their satisfactory performance. Bagging is a procedure that resamples data points to fit sequential trees so to improve predictive performance. A 10-fold cross validation was used to identify the optimal number of trees. The output of a BRT model consists of both predicted probabilities of occurrence and relative contributions (or influences) of predictors. The relative contribution is calculated based on how many times a predictor is chosen for splitting and how much each split improves the objective function, averaging over all trees. These relative contributions of all predictors are standardized so that they sum to one. A two-stage bootstrapping procedure was employed to provide a more robust and parsimonious estimation of model parameters. In each stage, the following split-and-fit step was repeated for a certain number of times. A training set with 80% of data points was randomly sampled without replacement, and the remaining 20% served as a test set. A BRT model was built using the training set, and then applied to the test set for validation if needed. In the first stage, the split-and-fitting step was repeated for ten times to screen important predictors. Validation of the trained model using the test set was not performed in this stage. Predictors that had a relative contribution <3% for all bootstrap training sets were excluded from the next stage. In the second stage, the split-and-fitting step was repeated for 100 times using the remaining predictors. As no variable selection was performed in this stage, all 100 models had the same predictors but yielded different contribution estimates. The relative contributions of the predictors were averaged over the 100 BRT models to represent their final relative contributions. The operating characteristic curve (ROC) curves and areas under the curve (AUC) based on the test sets were also averaged to represent the final predictive performance. The standard deviations and 95% percentiles of the relative contributions and AUCs across the 100 models were used to quantify the uncertainty in the estimation. Finally, the predicted probabilities were averaged over the 100 models to represent the final estimates of global probabilities of MPXV transmission presence. The threshold cutoff value used for final predictions of presence or absence was maximizes sensitivity + specificity along the ROC curve for the final BRT model, based on which we estimated the number of vulnerable human and area of risky district by overlaying population map (population count in 2020 from worldpop) with maps of MPXV-suitable areas.

**Supplementary reference:**

1. Arotolu TE, Afe AE, Wang H, Lv J, Shi K, Huang L, et al. Spatial modeling and ecological suitability of monkeypox disease in Southern Nigeria. *PLoS One*. 2022; 17(9): e0274325.

2. Fuller T, Thomassen HA, Mulembakani PM, Johnston SC, Lloyd-Smith JO, Kisalu NK, et al. Using remote sensing to map the risk of human monkeypox virus in the Congo Basin. *Ecohealth*. 2011;8(1):14-25.

3. Thomassen HA, Fuller T, Asefi-Najafabady S, Shiplacoff JA, Mulembakani PM, Blumberg S, et al. Pathogen-host associations and predicted range shifts of human monkeypox in response to climate change in central Africa. *PLoS One*. 2013; 8(7): e66071.

4. Esposito MM, Turku S, Lehrfield L, Shoman A. The impact of human activities on zoonotic infection transmissions. *Animals (Basel)*. 2023; 13(10): 1646.

5. Delany M J. Ecology of small rodents in Africa. *Mammal review*. 1986, 16(1): 1-41.

6. Olayemi A, Nicolas V, Hulselmans J, Missoup A, Fichet-Calvet E, Amundala D, et al. Taxonomy of the African giant pouched rats (Nesomyidae: Cricetomys): molecular and craniometric evidence support an unexpected high species diversity. *Zool J Linnean Society*. 2012; 165(3): 700-19.

7. Kennerley R. Cricetomys gambianus 2019 Available from: <https://www.iucnredlist.org/species/112169507/50534302>.

8. Hijmans R J, Phillips S, Leathwick J, et al. Package "dismo". *Circles*, 2017, 9(1): 1-68.

# Appendix Text S4: The risk of international spread for MPXV and metapopulation model

A limited number of studies already exist on the global risk of the monkeypox outbreak [1]. For instance, Manojit Bhattacharya et al. have provided valuable insights into the significant role of air travel in the international spread of monkeypox. Teng et al. [2] and Hiroki Nakamura et al. [3] have highlighted the use of air travel volume as a method to estimate the input risk of infectious diseases. Similar studies have also focused on other infectious diseases such as the Zika virus [3], yellow fever [4], and others [5]. Although the target of each study varies, some have common interests, both in terms of the role of airports or airlines in disease transmission and using spatial [6] or mapping information for analysis [5]. In light of these findings, the risks of importation and exportation of monkeypox were calculated based on origin–destination (OD) annual air travel flows [3]. The annual air travel flows used in this study were estimated based on Huang et al. and Hiroki Nakamura et al. [3, 7] models and developed two types of risk models to estimate the risk of international spread for monkeypox.

**Model 1-Risk Model from Endemic Countries**: The first risk model (Formula 1) utilized the proportion of the population at risk in endemic countries. The endemic countries for monkeypox were defined according to the criteria provided by the World Health Organization (WHO) [8]. These countries were considered endemic due to their historical record of monkeypox transmission. It considered the predicted probability of monkeypox presence in different regions (pixels) within each endemic country, as determined by the metapopulation model. The model factored in populations at risk in those regions to estimate the spatial distribution of monkeypox risk within each endemic country. This component focused on assessing the potential vulnerability of different regions within endemic countries and their contribution to the overall risk of international spread.

**Model 2-Risk Model from Epidemic Countries:** The second risk model (Formula 2) relied on the number of reported monkeypox cases in epidemic countries. It considered the annual air travel flux from each epidemic country to each destination city and used this information in conjunction with the reported case numbers to estimate the risk of monkeypox importation into the destination country. This component focused on assessing the risks associated with actual disease transmission events in epidemic countries and their potential impact on the spread of monkeypox to other countries.

Both risk models incorporated the origin-destination (OD) annual air travel flows obtained from the International Civil Aviation Organization (ICAO, https://data.icao.int/newDataPlus/Tools). Based on the network created from the assembled flight statistics, we calculated the degree, centrality, and strength of each node, and used these measurements as covariates in the modeling phase. The G-Econ data (http://gecon.yale.edu/) provide indices representing both market exchange rates (MER) and purchasing power parity (PPP) at a 1-degree longitude by 1-degree latitude resolution at a global scale. Due to the large geographical coverage of the grid cells, we extracted the closest PPP value for an airport and calculated the PPP value per capita by dividing the PPP by the population value within each grid cell. These data were utilized as local economic measurements for each airport [7]. These data were derived from a comprehensive metapopulation model [9, 10], which utilized information on passenger flows between 1623 airports on 10,400,836 unique routes with 17,063 airlines. The model considered various factors, such as network characteristics, city population, and local area GDP, from diverse and reliable sources [11].

The risk index ($r$) of MPXV population at risk importing to a destination country ($k$) from one origin country ($j$) was defined as:

$r_{jk1}=t_{jk}\frac{\sum_{i} {Pr}_{i}{Pop}_{i}}{{Pop}_{j}}$(Formula 1)

$r_{jk2}=t_{jk}\frac{\sum_{i} {Num}_{i}}{{Pop}_{j}}$(Formula 2)

The sum of imported risk to a destination city ($k$) from all endemic countries was then

$$R_{k}=\sum_{j} r_{jk}$$

$r_{jk1}$ and $r_{jk2}$ represented the risk index from the endemic country and epidemic country to a destination country, respectively. where $i$ was a pixel in the country $j$; ${Pr}_{i}$ was the predicted probability of a pixel in country $j$; ${Num}_{i}$ was the case number in country $j$; ${Pop}_{i}$ was the population of a pixel in country $j$; $t_{jk}$was the annual travel flux from country $j$ to city $k$; ${Pop}_{j}$ was the total population in the country $j$.

Finally, we computed the normalized (0-1) imported risk ($R$) by the following formula:

$$R_{Noramlized}=\frac{{log}_{10}R-{{(log}_{10}R)}_{min}}{{{(log}_{10}R)}_{max}-{{(log}_{10}R)}_{min}}$$

All data analyses were carried out using R version 4.0.3, graphical presentations were done using the R package “ggplot2” [12].

By applying both risk models, we were able to comprehensively assess the potential vulnerability of different countries to the importation and spread of monkeypox. The combination of these models allowed us to account for both the predicted spatial distribution of monkeypox risk within endemic countries and the actual reported of cases in epidemic countries. These risk estimates have significant implications for global disease surveillance and control efforts, helping inform targeted interventions and measures to prevent and respond to potential monkeypox outbreaks in different regions of the world.

**Supplementary reference:**

1. Bhattacharya M, Dhama K, Chakraborty C. Recently spreading human monkeypox virus infection and its transmission during COVID-19 pandemic period: A travelers' prospective. *Travel Med Infect Dis*. 2022; 49: 102398.

2. Teng AY, Che TL, Zhang AR, Zhang YY, Xu Q, Wang T, et al. Mapping the viruses belonging to the order Bunyavirales in China. *Infect Dis Poverty.* 2022; 11(1): 81.

3. Rocklöv J, Quam MB, Sudre B, German M, Kraemer MUG, Brady O, et al. Assessing seasonal risks for the introduction and mosquito-borne spread of Zika virus in Europe. *EBioMedicine*. 2016; 9: 250-256.

4. Shearer FM, Longbottom J, Browne AJ, Pigott DM, Brady OJ, Kraemer MUG, et al. Existing and potential infection risk zones of yellow fever worldwide: a modelling analysis. *Lancet Glob Health*. 2018; 6(3): e270-e278.

5. Leta S, Beyene TJ, De Clercq EM, Amenu K, Kraemer MUG, Revie CW. Global risk mapping for major diseases transmitted by Aedes aegypti and Aedes albopictus. *Int J Infect Dis*. 2018; 67: 25-35.

6. Hsu CI, Shih HH. Transmission and control of an emerging influenza pandemic in a small-world airline network. *Accid Anal Prev*. 2010; 42(1): 93-100

7. Huang Z, Wu X, Garcia AJ, Fik TJ, Tatem AJ. An open-access modeled passenger flow matrix for the global air network in 2010. *PLoS One*. 2013; 8(5): e64317.

8. WHO. Monkeypox OutbreakToolbox. 2022. https://cdn.who.int/media/docs/default-source/outbreak-toolkit/clean_monkey-pox-data-collection-toolbox_23may2022.pdf?sfvrsn=502d2ef3_1 (accessed May 31 2022).

9. Nakamura H, Managi S. Airport risk of importation and exportation of the COVID-19 pandemic. *Transp Policy* (Oxf). 2020; 96: 40-7.

10. Lee J, Choi BY, Jung E. Metapopulation model using commuting flow for national spread of the 2009 H1N1 influenza virus in the Republic of Korea. *J Theore biol.* 2018; 454: 320-9.

11. IATA. http://www.iata.org (accessed May 31 2022).

12. Singh G, Soman B. Data Visualisation using ggplot2 package in R; 2019.

# Appendix Text S5: Demographic characteristics and spatiotemporal changes of MPXV infections

The demographic characteristics of the patients differed among six reporting areas. A total of 288 deaths were reported globally, and Central Africa had a higher CFR (3.04%, 279/9173) than Western Africa (0.90%, 11/1226). The peak months with monkeypox case reporting differed among regions, appearing in October to December in Western Africa (46.52%, 127/273), June and July in America (97.74%, 4059/4153), and May and June Central Africa (92.60%, 763/824) (Table S14). Since 2000, the number of countries and the number of cases of monkeypox has been increasing every year (Figure S1). In 2022, a total of 16 754 human cases were reported in 72 countries. Among cases with known information, 96.46% (1609/1668) were male and most cases were aged 30–59 years (82.94%, 452/545), 95.83% (92/96) of patients were diagnosed via PCR, 56.86% (116/204) were outpatients, and 37.50% of cases were infected by travelling, 32.86% (163/496) of cases were infected by probable MSM-related infection, followed by community transmission (28.63%) (Table S15).

A total of 32 locations reporting infections in animals were identified in the literature during 1958–2017 (Table S17). These comprised 16 infections in rodents (e.g., *Funisciurus* spp.), six infections in monkeys (e.g., *Cercopithecus ascanius*), four in chimpanzees (Pan troglodytes), one infection in Eulipotyphla (*Petrodromus tetradactylus*) and two in others (fly and pig). MPXV infection in rodents mainly occurred in Democratic Republic of the Congo (DRC), except for the 2003 USA outbreak caused by international animal trade, and chimpanzees with positive detection of MPXV were only reported in Côte d'Ivoire, while other animals infected with MPXV were reported in both countries. Human cases had also been reported in all countries with animal positive detection of MPXV. In addition, 24 locations had reported animal-to-human transmission from 1982 to 2018 (Table S18).

# Appendix Table S1: Literature search syntax of monkeypox

| **Literature database** | **Website** | **Query** |
| --- | --- | --- |
| Web of Science | http://www.webofscience.com | TS = ("monkeypox virus" OR monkeypox OR "monkey pox" OR "orthopoxvirus") |
| PubMed | https://pubmed.ncbi.nlm.nih.gov | ("monkeypox virus" OR monkeypox OR "monkey pox" OR "orthopoxvirus "[Title/Abstract]) |
| GenBank | https://www.ncbi.nlm.nih.gov | "monkeypox virus"[Organism] AND "length > 18000 bp" |
| GIDEON | https://app.gideononline.com | Disease = "monkeypox" |

# Appendix Table S2: Information of MPXV infections from grey literature

| **Institution** | **Report Title** | **Website** |
| --- | --- | --- |
| WHO |  |  |
|  | Emergence of monkeypox in West Africa and Central Africa, 1970–2017 | https://apps.who.int/iris/bitstream/handle/10665/260497/WER9311.pdf;jsessionid=7AB72F28D04CFE6CE24996192FC478FF?sequence=1 |
|  | **Weekly Bulletin on Outbreak and other Emergencies: Week 11: 09 - 15 March 2020** | https://apps.who.int/iris/handle/10665/331451 |
|  | Weekly Bulletin on Outbreak and other Emergencies: Week 31: 29 July—04 August 2019 | https://apps.who.int/iris/handle/10665/326159?search-result=true&query=Weekly+Bulletin+on+Outbreak+and+other+Emergencies%3A+Week+31%3A+29+July-04+August+2019&scope=&rpp=10&sort_by=score&order=desc |
|  | Weekly Bulletin on Outbreaks and Other Emergencies. Week 16: 15—21 April 2017. | http://apps.who.int/iris/bitstream/handle/10665/255050/OEW16-152142017.pdf;jsessionid=194584C7F298C4F3B1307F5C21581531?sequence=1 |
|  | Multi-country outbreak of monkeypox, External situation report #1 - 6 July 2022 | https://cdn.who.int/media/docs/default-source/2021-dha-docs/20220706_monkeypox_external_sitrep_final.pdf?sfvrsn=1b580b3d_4&download=true |
|  | Multi-country outbreak of monkeypox, External situation report #2 - 25 July 2022 | https://www.who.int/docs/default-source/coronaviruse/situation-reports/20220725_monkeypox_external_sitrep_2_final.pdf?sfvrsn=c41fc2dd_3&download=true |
|  | Multi-country monkeypox outbreak: situation update | https://www.who.int/emergencies/disease-outbreak-news/item/2022-DON396 |
|  | Multi-country monkeypox outbreak: situation update | https://www.who.int/emergencies/disease-outbreak-news/item/2022-DON393 |
|  | Multi-country monkeypox outbreak: situation update | https://www.who.int/emergencies/disease-outbreak-news/item/2022-DON392 |
|  | Multi-country monkeypox outbreak: situation update | https://www.who.int/emergencies/disease-outbreak-news/item/2022-DON390 |
|  | Multi-country monkeypox outbreak in non-endemic countries: Update | https://www.who.int/emergencies/disease-outbreak-news/item/2022-DON388 |
|  | Multi-country monkeypox outbreak in non-endemic countries | https://www.who.int/emergencies/disease-outbreak-news/item/2022-DON385 |
|  | Monkeypox - United Kingdom of Great Britain and Northern Ireland | https://www.who.int/emergencies/disease-outbreak-news/item/2022-DON383 |
|  | Monkeypox - United Kingdom of Great Britain and Northern Ireland | https://www.who.int/emergencies/disease-outbreak-news/item/2022-DON381 |
|  | Monkeypox - United States of America | https://www.who.int/emergencies/disease-outbreak-news/item/2021-DON344 |
|  | Monkeypox - United States of America | https://www.who.int/emergencies/disease-outbreak-news/item/monkeypox---the-united-states-of-america |
|  | Monkeypox - United Kingdom of Great Britain and Northern Ireland | https://www.who.int/emergencies/disease-outbreak-news/item/monkeypox---united-kingdom-of-great-britain-and-northern-ireland |
|  | Monkeypox - Singapore | https://www.who.int/emergencies/disease-outbreak-news/item/16-may-2019-monkeypox-singapore-en |
|  | Monkeypox - United Kingdom of Great Britain and Northern Ireland | https://www.who.int/emergencies/disease-outbreak-news/item/monkeypox---united-kingdom-of-great-britain-and-northern-ireland-ex-nigeria |
|  | Monkeypox - Democratic Republic of the Congo | https://www.who.int/emergencies/disease-outbreak-news/item/1997_03_21c-en |
|  | Monkeypox - African Region (AFRO) | https://www.who.int/emergencies/disease-outbreak-news/item/1997_04_14-en |
|  | Monkeypox - African Region (AFRO) | https://www.who.int/emergencies/disease-outbreak-news/item/1997_04_24b-en |
|  | Monkeypox - Democratic Republic of the Congo | https://www.who.int/emergencies/disease-outbreak-news/item/1997_07_31b-en |
|  | Monkeypox - Democratic Republic of the Congo | https://www.who.int/emergencies/disease-outbreak-news/item/1997_11_14b-en |
|  | Monkeypox - Nigeria | https://www.who.int/emergencies/disease-outbreak-news/item/21-december-2017-monkeypox-nigeria-en |
|  | Monkeypox - Cameroon | https://www.who.int/emergencies/disease-outbreak-news/item/05-june-2018-monkeypox-cameroon-en |
|  | Monkeypox - Nigeria | https://www.who.int/emergencies/disease-outbreak-news/item/05-october-2018-monkeypox-nigeria-en |
|  | Monkeypox - Democratic Republic of the Congo | https://www.who.int/emergencies/disease-outbreak-news/item/monkeypox-democratic-republic-of-the-congo |
|  | Emergence of monkeypox in West Africa and Central Africa, 1970–2017 | https://www.who.int/publications/i/item/10665-260497 |
|  | Monkeypox - Central African Republic | https://www.who.int/emergencies/disease-outbreak-news/item/13-october-2016-monkeypox-caf-en |
| CDC |  |  |
|  | U.S. Monkeypox 2022: Situation Summary | https://www.cdc.gov/poxvirus/monkeypox/response/2022/index.html |
|  | Monkeypox in the United States-July 2021 Travel-Associated Case | https://www.cdc.gov/poxvirus/monkeypox/outbreak/us-outbreaks.html |
|  | Monkeypox in the United States-November 2021 Travel-Associated Case | https://www.cdc.gov/poxvirus/monkeypox/outbreak/us-outbreaks.html https://health.maryland.gov/newsroom/Pages/Travel-Associated-Monkeypox-virus-infection-confirmed-in-Maryland-resident.aspx |
| NCDC |  |  |
|  | An Update of Monkeypox Outbreak in Nigeria for Week 28 | https://ncdc.gov.ng/diseases/sitreps/?cat=8&name=An%20Update%20of%20Monkeypox%20Outbreak%20in%20Nigeria |
|  | An Update of Monkeypox Outbreak in Nigeria for Week 27 | https://ncdc.gov.ng/diseases/sitreps/?cat=8&name=An%20Update%20of%20Monkeypox%20Outbreak%20in%20Nigeria |
|  | An Update of Monkeypox Outbreak in Nigeria for Week 26 | https://ncdc.gov.ng/diseases/sitreps/?cat=8&name=An%20Update%20of%20Monkeypox%20Outbreak%20in%20Nigeria |
|  | An Update of Monkeypox Outbreak in Nigeria for Week 25 | https://ncdc.gov.ng/diseases/sitreps/?cat=8&name=An%20Update%20of%20Monkeypox%20Outbreak%20in%20Nigeria |
|  | An Update of Monkeypox Outbreak in Nigeria for Week 24 | https://ncdc.gov.ng/diseases/sitreps/?cat=8&name=An%20Update%20of%20Monkeypox%20Outbreak%20in%20Nigeria |
|  | An Update of Monkeypox Outbreak in Nigeria for Week 23 | https://ncdc.gov.ng/diseases/sitreps/?cat=8&name=An%20Update%20of%20Monkeypox%20Outbreak%20in%20Nigeria |
|  | An Update of Monkeypox Outbreak in Nigeria for Week 22 | https://ncdc.gov.ng/diseases/sitreps/?cat=8&name=An%20Update%20of%20Monkeypox%20Outbreak%20in%20Nigeria |
|  | An Update of Monkeypox Outbreak in Nigeria for Week 21 | https://ncdc.gov.ng/diseases/sitreps/?cat=8&name=An%20Update%20of%20Monkeypox%20Outbreak%20in%20Nigeria |
|  | An Update of Monkeypox Outbreak in Nigeria for Week 17 | https://ncdc.gov.ng/diseases/sitreps/?cat=8&name=An%20Update%20of%20Monkeypox%20Outbreak%20in%20Nigeria |
|  | An Update of Monkeypox Outbreak in Nigeria for Week 13 | https://ncdc.gov.ng/diseases/sitreps/?cat=8&name=An%20Update%20of%20Monkeypox%20Outbreak%20in%20Nigeria |
|  | An Update of Monkeypox Outbreak in Nigeria for Week 8 | https://ncdc.gov.ng/diseases/sitreps/?cat=8&name=An%20Update%20of%20Monkeypox%20Outbreak%20in%20Nigeria |
|  | An Update of Monkeypox Outbreak in Nigeria for Week 4 | https://ncdc.gov.ng/diseases/sitreps/?cat=8&name=An%20Update%20of%20Monkeypox%20Outbreak%20in%20Nigeria |
|  | An Update of Monkeypox Outbreak in Nigeria for Week 52 | https://ncdc.gov.ng/diseases/sitreps/?cat=8&name=An%20Update%20of%20Monkeypox%20Outbreak%20in%20Nigeria |
|  | An Update of Monkeypox Outbreak in Nigeria for Week 47 | https://ncdc.gov.ng/diseases/sitreps/?cat=8&name=An%20Update%20of%20Monkeypox%20Outbreak%20in%20Nigeria |
|  | An Update of Monkeypox Outbreak in Nigeria for Week 43 | https://ncdc.gov.ng/diseases/sitreps/?cat=8&name=An%20Update%20of%20Monkeypox%20Outbreak%20in%20Nigeria |
|  | An Update of Monkeypox Outbreak in Nigeria for Week 38 | https://ncdc.gov.ng/diseases/sitreps/?cat=8&name=An%20Update%20of%20Monkeypox%20Outbreak%20in%20Nigeria |
|  | An Update of Monkeypox Outbreak in Nigeria for Week 34 | https://ncdc.gov.ng/diseases/sitreps/?cat=8&name=An%20Update%20of%20Monkeypox%20Outbreak%20in%20Nigeria |
|  | An Update of Monkeypox Outbreak in Nigeria for Week 30 | https://ncdc.gov.ng/diseases/sitreps/?cat=8&name=An%20Update%20of%20Monkeypox%20Outbreak%20in%20Nigeria |
|  | An Update of Monkeypox Outbreak in Nigeria for Week 26 | https://ncdc.gov.ng/diseases/sitreps/?cat=8&name=An%20Update%20of%20Monkeypox%20Outbreak%20in%20Nigeria |
|  | An Update of Monkeypox Outbreak in Nigeria for Week 21 | https://ncdc.gov.ng/diseases/sitreps/?cat=8&name=An%20Update%20of%20Monkeypox%20Outbreak%20in%20Nigeria |
|  | An Update of Monkeypox Outbreak in Nigeria for Week 53 | https://ncdc.gov.ng/diseases/sitreps/?cat=8&name=An%20Update%20of%20Monkeypox%20Outbreak%20in%20Nigeria |
|  | An Update of Monkeypox Outbreak in Nigeria for Week 48 | https://ncdc.gov.ng/diseases/sitreps/?cat=8&name=An%20Update%20of%20Monkeypox%20Outbreak%20in%20Nigeria |
|  | An Update of Monkeypox Outbreak in Nigeria for Week 44 | https://ncdc.gov.ng/diseases/sitreps/?cat=8&name=An%20Update%20of%20Monkeypox%20Outbreak%20in%20Nigeria |
|  | An Update of Monkeypox Outbreak in Nigeria for Week 40 | https://ncdc.gov.ng/diseases/sitreps/?cat=8&name=An%20Update%20of%20Monkeypox%20Outbreak%20in%20Nigeria |
|  | An Update of Monkeypox Outbreak in Nigeria for Week 35 | https://ncdc.gov.ng/diseases/sitreps/?cat=8&name=An%20Update%20of%20Monkeypox%20Outbreak%20in%20Nigeria |
|  | An Update of Monkeypox Outbreak in Nigeria for Week 5 | https://ncdc.gov.ng/diseases/sitreps/?cat=8&name=An%20Update%20of%20Monkeypox%20Outbreak%20in%20Nigeria |
|  | An Update of Monkeypox Outbreak in Nigeria for Week 50 | https://ncdc.gov.ng/diseases/sitreps/?cat=8&name=An%20Update%20of%20Monkeypox%20Outbreak%20in%20Nigeria |
|  | An Update of Monkeypox Outbreak in Nigeria for Week 46 | https://ncdc.gov.ng/diseases/sitreps/?cat=8&name=An%20Update%20of%20Monkeypox%20Outbreak%20in%20Nigeria |
|  | An Update of Monkeypox Outbreak in Nigeria for Week 41 | https://ncdc.gov.ng/diseases/sitreps/?cat=8&name=An%20Update%20of%20Monkeypox%20Outbreak%20in%20Nigeria |
|  | An Update of Monkeypox Outbreak in Nigeria for Week 37 | https://ncdc.gov.ng/diseases/sitreps/?cat=8&name=An%20Update%20of%20Monkeypox%20Outbreak%20in%20Nigeria |
|  | An Update of Monkeypox Outbreak in Nigeria for Week 9 | https://ncdc.gov.ng/diseases/sitreps/?cat=8&name=An%20Update%20of%20Monkeypox%20Outbreak%20in%20Nigeria |
|  | An Update of Monkeypox Outbreak in Nigeria for Week 4 | https://ncdc.gov.ng/diseases/sitreps/?cat=8&name=An%20Update%20of%20Monkeypox%20Outbreak%20in%20Nigeria |
|  | An Update of Monkeypox Outbreak in Nigeria for Week 52 | https://ncdc.gov.ng/diseases/sitreps/?cat=8&name=An%20Update%20of%20Monkeypox%20Outbreak%20in%20Nigeria |
|  | An Update of Monkeypox Outbreak in Nigeria for Week 50 | https://ncdc.gov.ng/diseases/sitreps/?cat=8&name=An%20Update%20of%20Monkeypox%20Outbreak%20in%20Nigeria |
|  | An Update of Monkeypox Outbreak in Nigeria for Week 48 | https://ncdc.gov.ng/diseases/sitreps/?cat=8&name=An%20Update%20of%20Monkeypox%20Outbreak%20in%20Nigeria |
|  | An Update of Monkeypox Outbreak in Nigeria for Week 48 | https://ncdc.gov.ng/diseases/sitreps/?cat=8&name=An%20Update%20of%20Monkeypox%20Outbreak%20in%20Nigeria |
|  | An Update of Monkeypox Outbreak in Nigeria for Week 45 | https://ncdc.gov.ng/diseases/sitreps/?cat=8&name=An%20Update%20of%20Monkeypox%20Outbreak%20in%20Nigeria |
|  | An Update of Monkeypox Outbreak in Nigeria for Week 43 | https://ncdc.gov.ng/diseases/sitreps/?cat=8&name=An%20Update%20of%20Monkeypox%20Outbreak%20in%20Nigeria |
|  | An Update of Monkeypox Outbreak in Nigeria for Week 43 | https://ncdc.gov.ng/diseases/sitreps/?cat=8&name=An%20Update%20of%20Monkeypox%20Outbreak%20in%20Nigeria |
|  | An Update of Monkeypox Outbreak in Nigeria for Week 42 | https://ncdc.gov.ng/diseases/sitreps/?cat=8&name=An%20Update%20of%20Monkeypox%20Outbreak%20in%20Nigeria |

# Appendix Table S3: Guideline for inclusion and exclusion of literature of MPXV infections

| **Inclusion** | **Exclusion** |
| --- | --- |
| (i) Case report and surveillance of human monkeypox infection | (i) Duplicates literature for monkeypox |
| (ii) Case report or surveillance of animal monkeypox infection | (ii) Not related to monkeypox virus |
| (iii) Outbreak investigation of monkeypox infections | (iii) No data extracted |
| (ⅳ) Epidemiology study of monkeypox infections in human or animals | (ⅳ) Molecular mechanism |
|  | (ⅴ) Review or comment |
|  | (ⅵ) Vaccine trials for monkeypox virus |
|  | (ⅶ) Laboratory methods for monkeypox |
|  | (ⅷ) Drug trial for monkeypox |

# Appendix Table S4: Data extracted for literature included in the study

| **Variable** | **Guidance** |
| --- | --- |
| Basic information |  |
| Literature ID | Unique identifier assigned to a literature. |
| Literature title | Literature title that included in the review. |
| Publish year | Publication year of the article identified for data extraction. |
| Investigating Date | Record the date for the period over which the reported study was conducted. |
| Transmission model | Including infections within the family, community transmission, local natural infection, in-hospital infections, animal trade infections, travel related and unknown. |
| Geographic information |  |
| Country | Geographic location information recorded at country-level. |
| First administrative level | Geographic location information recorded at first-level. |
| Second administrative level | Geographic location information recorded at second-level. |
| Third administrative level | Geographic location information recorded at third-level. |
| Specific location | Record the infection or detection out of third-level. |
| longitude | The longitude of the study site. |
| latitude | The latitude of the study site. |
| Laboratory information |  |
| Testing method | Record the detection testing method used, e.g., PCR, RT-PCR or ELISA. |
| Testing specimen | Record the sample tested for the diagnosis. e.g., blood, CSF. |
| Number of individuals tested all | Record the number of individuals tested for specific pathogen by testing method. |
| Number of individuals diagnosed as positive | Record the number of individuals diagnosed as positive for specific pathogen by testing method. |
| Clinical Information |  |
| Number of cases (Type of infections) | Record the number of human infections of MPXV (confirmed case/ suspect or probable case). |
| Inpatient | Hospitalization or not. |
| Outcome | Record the outcome of MPXV infections. |
| Co-infection | Record the co-infection information of MPXV infections. |
| Clinical symptoms | Record the clinical presentations (including fever, headache, chills, cough, rush, skin lesions, pruritis, diarrhea, muscle pain, sore throat, conjunctiva, lymphadenopathy and mouth ulcer) and the positive number. |
| Treatment | Record the treatment of MPXV infections (including: drug, methods and time). |
| Underlying diseases or specific immune states | Record whether patients have underlying diseases or special immune states. |
| Human information |  |
| Male/ Female | Record the gender ration of MPXV infections. |
| Mean age or median age | Record the mean age or median age information of MPXV infections. |
| Age range | Record the age range information of MPXV infections. |
| Age distribution | Record the age distribution of MPXV infections. |
| Occupation | Record the occupation of MPXV infections. |
| Epidemiological information |  |
| Year of onset | Record the year of onset of MPXV infections. |
| Month of onset | Record the month of onset of MPXV infections. |
| Clustering | Whether the patient is a cluster of cases |
| Exposure site | Record the exposure site (e.g., community or hospital). |
| Contact or field history | Record the contact history (e.g., animals ). |
| Imported case | Record whether the patient is an imported case. |
| Incubation period | Record the incubation period of MPXV infections. |
| Onset days | Record the onset days of MPXV infections. |
| Range of onset days | Record the Range of onset days of MPXV infections. |
| Delayed | Record the delayed period of MPXV infections. |
| Survival period | Record the survival period of MPXV infections. |
| Type of transmission | Whether the cases were zoonotic or person-to-person. |
| Immunization Information |  |
| Smallpox vaccination | Whether the patient was vaccinated against smallpox. |

# Appendix Table S5: Criteria of human and animal infections with MPXV

| Case type | Note | Reference |
| --- | --- | --- |
| Human infections |  |  |
| Suspect or probable case | 1. New characteristic rash AND meets one of the epidemiologic links with confirmed case.  2. New characteristic rash AND reported travel history to a monkeypox endemic countries*.  3. New characteristic rash AND has had multiple or anonymous sexual partners in the 21 days before symptom onset (since May, 2022).  4. Has a positive result of an orthopoxvirus serological assay, in the absence of smallpox vaccination or other known exposure to orthopoxviruses  5. No suspicion of other recent Orthopoxvirus exposure (e.g., Vaccinia virus in ACAM2000 vaccination) AND demonstration of the presence of testing methods (e.g., Orthopoxvirus DNA by polymerase chain reaction of a clinical specimen OR Orthopoxvirus using immunohistochemical or electron microscopy testing methods OR Demonstration of detectable levels of anti-orthopoxvirus IgM antibody during the period of 4 to 56 days after rash onset) | 1. WHO. WHO suggested outbreak case definition for the multi-country monkeypox outbreak, as of 21 May 2022. https://www.who.int/emergencies/outbreak-toolkit/disease-outbreak-toolboxes/monkeypox-outbreak-toolbox (accessed Jun 8, 2022).  2. CDC. Case Definitions for Use in the 2022 Monkeypox Response, as of 8 June 2022. https://www.cdc.gov/poxvirus/monkeypox/clinicians/case-definition.html (accessed Jun 12, 2022).  3. NCDC. Monkeypox, as of 21 May 2022. https://ncdc.gov.ng/diseases/info/M#diagnosis_testing (accessed Jun 12, 2022). |
| Confirmed case | Demonstration of the presence of Monkeypox virus DNA by polymerase chain reaction testing or Next-Generation sequencing of a clinical specimen OR isolation of Monkeypox virus in culture from a clinical specimen |  |
| Zoonotic transmission | Direct contact with the virus through the bite or scratch of an infected animal, by handing wild game, or through the usage of products made from infected animals | 1. CDC US. Monkeypox Cases. 2022. https://www.cdc.gov/poxvirus/monkeypox/transmission.html (accessed Jun 9, 2022). |
| Human-to-human transmission | Direct contact with body fluids or sores on an infected person, or exposure to respiratory secretions during prolonged, face-to-face contact |  |
| Mixed transmission | The existence of both zoonotic and human-to-human transmission |  |
| Animal infections |  |  |
| Confirmed case | The diagnosis can be confirmed by virus isolation or assays for genetic material, such as PCR. Monkeypox virus may be detected in skin lesions or samples from affected organs at necropsy, and sometimes in conjunctival swabs or oral and nasal secretions (e.g., oropharyngeal swabs). | 1. WOAH. Questions and Answers on Monkeypox and Animals. 2022. https://www.woah.org/en/disease/monkeypox (accessed May 30, 2022). |

*: Monkeypox endemic countries include Benin, Cameroon, Central African Republic, Democratic Republic of the Congo (DRC), Gabon, Ghana, Côte d'Ivoire, Liberia, Nigeria, the Republic of the Congo, and Sierra Leone.

# Appendix Table S6: Genome sequences used in the phylogenetic analyses

| ID | GenBank | Year | Country | Region | Clade |
| --- | --- | --- | --- | --- | --- |
| 1 | KC257459 | 2005 | Sudan | Africa | Congo Basin clade |
| 2 | KP849470 | 1971 | Côte d'Ivoire | Africa | West African clade |
| 3 | DQ011156 | 1970 | Liberia | Africa | West African clade |
| 4 | KJ136820 | 2012 | Côte d'Ivoire | Africa | West African clade |
| 5 | AY753185 | 1958 |  | Africa | West African clade |
| 6 | JX878410 | 2006 | Democratic Republic of the Congo | Africa | Congo Basin clade |
| 7 | JX878411 | 2006 | Democratic Republic of the Congo | Africa | Congo Basin clade |
| 8 | DQ011153 | 2003 |  | North America | West African clade |
| 9 | DQ011157 | 2003 |  | North America | West African clade |
| 10 | MT903347 | 2003 | USA | North America | West African clade |
| 11 | MT903348 | 2003 | USA | North America | West African clade |
| 12 | MT903346 | 2003 | USA | North America | West African clade |
| 13 | AY741551 | 1970 | Sierra Leone | Africa | West African clade |
| 14 | KJ642616 | 1968 | France | Europe | West African clade |
| 15 | JX878412 | 2006 | Democratic Republic of the Congo | Africa | Congo Basin clade |
| 16 | JX878409 | 2006 | Democratic Republic of the Congo | Africa | Congo Basin clade |
| 17 | MN346691 | 2017 | Côte d'Ivoire | Africa | West African clade |
| 18 | MN346703 | 2018 | Côte d'Ivoire | Africa | West African clade |
| 19 | MN346701 | 2017 | Côte d'Ivoire | Africa | West African clade |
| 20 | MN346690 | 2017 | Côte d'Ivoire | Africa | West African clade |
| 21 | MN346692 | 2017 | Côte d'Ivoire | Africa | West African clade |
| 22 | MN346694 | 2017 | Côte d'Ivoire | Africa | West African clade |
| 23 | MN346697 | 2017 | Côte d'Ivoire | Africa | West African clade |
| 24 | MN346696 | 2017 | Côte d'Ivoire | Africa | West African clade |
| 25 | MN346695 | 2017 | Côte d'Ivoire | Africa | West African clade |
| 26 | MN346702 | 2018 | Côte d'Ivoire | Africa | West African clade |
| 27 | MN346698 | 2017 | Côte d'Ivoire | Africa | West African clade |
| 28 | MN346699 | 2017 | Côte d'Ivoire | Africa | West African clade |
| 29 | MN346700 | 2017 | Côte d'Ivoire | Africa | West African clade |
| 30 | ON622713 | 2022 | Belgium | Europe | West African clade |
| 31 | OX009124 | 2022 | Sweden | Europe | West African clade |
| 32 | ON622712 | 2022 | Belgium | Europe | West African clade |
| 33 | ON880420 | 2022 | Belgium | Europe | West African clade |
| 34 | ON880419 | 2022 | Belgium | Europe | West African clade |
| 35 | JX878417 | 2006 | Democratic Republic of the Congo | Africa | Congo Basin clade |
| 36 | JX878414 | 2006 | Democratic Republic of the Congo | Africa | Congo Basin clade |
| 37 | ON880422 | 2022 | Belgium | Europe | West African clade |
| 38 | ON959136 | 2022 | USA | North America | West African clade |
| 39 | KJ642615 | 1978 | Nigeria | Africa | West African clade |
| 40 | ON959133 | 2022 | USA | North America | West African clade |
| 41 | JX878421 | 2007 | Democratic Republic of the Congo | Africa | Congo Basin clade |
| 42 | ON954773 | 2022 | USA | North America | West African clade |
| 43 | ON959135 | 2022 | USA | North America | West African clade |
| 44 | ON959132 | 2022 | USA | North America | West African clade |
| 45 | ON959131 | 2022 | USA | North America | West African clade |
| 46 | ON959134 | 2022 | USA | North America | West African clade |
| 47 | ON754987 | 2022 | Slovenia | Europe | West African clade |
| 48 | ON838178 | 2022 | Slovenia | Europe | West African clade |
| 49 | ON754985 | 2022 | Slovenia | Europe | West African clade |
| 50 | JX878416 | 2006 | Democratic Republic of the Congo | Africa | Congo Basin clade |
| 51 | ON880421 | 2022 | Belgium | Europe | West African clade |
| 52 | JX878413 | 2006 | Democratic Republic of the Congo | Africa | Congo Basin clade |
| 53 | ON754984 | 2022 | Slovenia | Europe | West African clade |
| 54 | ON754986 | 2022 | Slovenia | Europe | West African clade |
| 55 | MK783027 | 2017 | Nigeria | Africa | West African clade |
| 56 | MK783028 | 2017 | Nigeria | Africa | West African clade |
| 57 | MK783029 | 2017 | Nigeria | Africa | West African clade |
| 58 | MK783030 | 2017 | Nigeria | Africa | West African clade |
| 59 | MK783031 | 2017 | Nigeria | Africa | West African clade |
| 60 | MK783032 | 2017 | Nigeria | Africa | West African clade |
| 61 | MK783033 | 2017 | Nigeria | Africa | West African clade |
| 62 | MT903338 | 2018 | Nigeria | Africa | West African clade |
| 63 | MT903339 | 2018 | Nigeria | Africa | West African clade |
| 64 | KJ642617 | 1971 | Nigeria | Africa | West African clade |
| 65 | MT250197 | 2019 | Singapore | Asia | West African clade |
| 66 | MT903337 | 2018 | Nigeria | Africa | West African clade |
| 67 | ON609725 | 2022 | Slovenia | Europe | West African clade |
| 68 | ON631241 | 2022 | Slovenia | Europe | West African clade |
| 69 | JX878415 | 2006 | Democratic Republic of the Congo | Africa | Congo Basin clade |
| 70 | JX878428 | 2007 | Democratic Republic of the Congo | Africa | Congo Basin clade |
| 71 | ON631963 | 2022 | Australia | Oceania | West African clade |
| 72 | ON694331 | 2022 | Germany | Europe | West African clade |
| 73 | ON694338 | 2022 | Germany | Europe | West African clade |
| 74 | KP849469 | 2008 | Democratic Republic of the Congo | Africa | Congo Basin clade |
| 75 | ON751962 | 2022 | Brazil | South America | West African clade |
| 76 | ON880413 | 2022 | Brazil | South America | West African clade |
| 77 | MN648051 | 2018 | Israel | Asia | West African clade |
| 78 | ON644344 | 2022 | Italy | Europe | West African clade |
| 79 | ON694339 | 2022 | Germany | Europe | West African clade |
| 80 | ON911481 | 2022 | Mexico | North America | West African clade |
| 81 | ON568298 | 2022 | Germany | Europe | West African clade |
| 82 | JX878427 | 2007 | Democratic Republic of the Congo | Africa | Congo Basin clade |
| 83 | ON694340 | 2022 | Germany | Europe | West African clade |
| 84 | JX878422 | 2007 | Democratic Republic of the Congo | Africa | Congo Basin clade |
| 85 | ON694337 | 2022 | Germany | Europe | West African clade |
| 86 | ON918656 | 2022 | Taiwan | Asia | West African clade |
| 87 | MT903342 | 2019 | Singapore | Asia | West African clade |
| 88 | ON694336 | 2022 | Germany | Europe | West African clade |
| 89 | ON780017 | 2022 | Italy | Europe | West African clade |
| 90 | ON694329 | 2022 | Germany | Europe | West African clade |
| 91 | ON676707 | 2021 | USA | North America | West African clade |
| 92 | KP849471 | 1985 | Democratic Republic of the Congo | Africa | Congo Basin clade |
| 93 | ON694334 | 2022 | Germany | Europe | West African clade |
| 94 | MT903344 | 2018 | United Kingdom | Europe | West African clade |
| 95 | ON843176 | 2022 | Portugal | Europe | West African clade |
| 96 | ON782055 | 2022 | Spain | Europe | West African clade |
| 97 | ON803427 | 2022 | Canada | North America | West African clade |
| 98 | ON843163 | 2022 | Portugal | Europe | West African clade |
| 99 | ON843164 | 2022 | Portugal | Europe | West African clade |
| 100 | ON843165 | 2022 | Portugal | Europe | West African clade |
| 101 | ON843166 | 2022 | Portugal | Europe | West African clade |
| 102 | ON843167 | 2022 | Portugal | Europe | West African clade |
| 103 | ON843168 | 2022 | Portugal | Europe | West African clade |
| 104 | ON843169 | 2022 | Portugal | Europe | West African clade |
| 105 | ON843170 | 2022 | Portugal | Europe | West African clade |
| 106 | ON843172 | 2022 | Portugal | Europe | West African clade |
| 107 | ON843173 | 2022 | Portugal | Europe | West African clade |
| 108 | ON843174 | 2022 | Portugal | Europe | West African clade |
| 109 | ON843177 | 2022 | Portugal | Europe | West African clade |
| 110 | ON843178 | 2022 | Portugal | Europe | West African clade |
| 111 | ON585033 | 2022 | Portugal | Europe | West African clade |
| 112 | ON585035 | 2022 | Portugal | Europe | West African clade |
| 113 | ON880541 | 2022 | Canada | North America | West African clade |
| 114 | ON843179 | 2022 | Portugal | Europe | West African clade |
| 115 | OP013002 | 2022 | Canada | North America | West African clade |
| 116 | ON745215 | 2022 | Italy | Europe | West African clade |
| 117 | ON780016 | 2022 | Italy | Europe | West African clade |
| 118 | ON585032 | 2022 | Portugal | Europe | West African clade |
| 119 | ON803437 | 2022 | Canada | North America | West African clade |
| 120 | ON803441 | 2022 | Canada | North America | West African clade |
| 121 | ON803442 | 2022 | Canada | North America | West African clade |
| 122 | ON694333 | 2022 | Germany | Europe | West African clade |
| 123 | MT903340 | 2018 | Nigeria | Africa | West African clade |
| 124 | NC_063383 | 2018 | Nigeria | Africa | West African clade |
| 125 | ON803417 | 2022 | Canada | North America | West African clade |
| 126 | ON803421 | 2022 | Canada | North America | West African clade |
| 127 | ON803422 | 2022 | Canada | North America | West African clade |
| 128 | ON803423 | 2022 | Canada | North America | West African clade |
| 129 | ON803424 | 2022 | Canada | North America | West African clade |
| 130 | ON803425 | 2022 | Canada | North America | West African clade |
| 131 | ON843171 | 2022 | Portugal | Europe | West African clade |
| 132 | ON843180 | 2022 | Portugal | Europe | West African clade |
| 133 | ON585030 | 2022 | Portugal | Europe | West African clade |
| 134 | ON585031 | 2022 | Portugal | Europe | West African clade |
| 135 | ON585034 | 2022 | Portugal | Europe | West African clade |
| 136 | ON803430 | 2022 | Canada | North America | West African clade |
| 137 | ON843175 | 2022 | Portugal | Europe | West African clade |
| 138 | ON880537 | 2022 | Canada | North America | West African clade |
| 139 | MT903345 | 2018 | United Kingdom | Europe | West African clade |
| 140 | ON563414 | 2022 | USA | North America | West African clade |
| 141 | ON619835 | 2022 | United Kingdom | Europe | West African clade |
| 142 | ON619836 | 2022 | United Kingdom | Europe | West African clade |
| 143 | ON619838 | 2022 | United Kingdom | Europe | West African clade |
| 144 | ON736420 | 2022 | Canada | North America | West African clade |
| 145 | ON745225 | 2022 | Spain | Europe | West African clade |
| 146 | ON754989 | 2022 | Canada | North America | West African clade |
| 147 | ON792322 | 2022 | Switzerland | Europe | West African clade |
| 148 | OP022170 | 2022 | United Kingdom | Europe | West African clade |
| 149 | ON649709 | 2022 | Portugal | Europe | West African clade |
| 150 | ON649718 | 2022 | Portugal | Europe | West African clade |
| 151 | ON649719 | 2022 | Portugal | Europe | West African clade |
| 152 | ON649720 | 2022 | Portugal | Europe | West African clade |
| 153 | ON649721 | 2022 | Portugal | Europe | West African clade |
| 154 | ON649722 | 2022 | Portugal | Europe | West African clade |
| 155 | ON649723 | 2022 | Portugal | Europe | West African clade |
| 156 | ON649724 | 2022 | Portugal | Europe | West African clade |
| 157 | ON649725 | 2022 | Portugal | Europe | West African clade |
| 158 | ON803426 | 2022 | Canada | North America | West African clade |
| 159 | ON803440 | 2022 | Canada | North America | West African clade |
| 160 | ON803443 | 2022 | Canada | North America | West African clade |
| 161 | ON880514 | 2022 | Canada | North America | West African clade |
| 162 | ON880515 | 2022 | Canada | North America | West African clade |
| 163 | ON880544 | 2022 | Canada | North America | West African clade |
| 164 | ON983161 | 2022 | Canada | North America | West African clade |
| 165 | OP013003 | 2022 | Canada | North America | West African clade |
| 166 | OP013012 | 2022 | Canada | North America | West African clade |
| 167 | OP013017 | 2022 | Canada | North America | West African clade |
| 168 | ON803418 | 2022 | Canada | North America | West African clade |
| 169 | ON880533 | 2022 | Canada | North America | West African clade |
| 170 | ON803438 | 2022 | Canada | North America | West African clade |
| 171 | ON803413 | 2022 | Canada | North America | West African clade |
| 172 | ON803414 | 2022 | Canada | North America | West African clade |
| 173 | ON803420 | 2022 | Canada | North America | West African clade |
| 174 | ON803429 | 2022 | Canada | North America | West African clade |
| 175 | ON803431 | 2022 | Canada | North America | West African clade |
| 176 | ON803432 | 2022 | Canada | North America | West African clade |
| 177 | ON803435 | 2022 | Canada | North America | West African clade |
| 178 | ON803439 | 2022 | Canada | North America | West African clade |
| 179 | OP019275 | 2022 | Austria | Europe | West African clade |
| 180 | ON585036 | 2022 | Portugal | Europe | West African clade |
| 181 | ON649708 | 2022 | Portugal | Europe | West African clade |
| 182 | ON649710 | 2022 | Portugal | Europe | West African clade |
| 183 | ON649712 | 2022 | Portugal | Europe | West African clade |
| 184 | ON649717 | 2022 | Portugal | Europe | West African clade |
| 185 | ON694330 | 2022 | Germany | Europe | West African clade |
| 186 | ON803419 | 2022 | Canada | North America | West African clade |
| 187 | ON880524 | 2022 | Canada | North America | West African clade |
| 188 | ON880528 | 2022 | Canada | North America | West African clade |
| 189 | ON880529 | 2022 | Canada | North America | West African clade |
| 190 | ON880532 | 2022 | Canada | North America | West African clade |
| 191 | ON880535 | 2022 | Canada | North America | West African clade |
| 192 | ON880549 | 2022 | Canada | North America | West African clade |
| 193 | OP013006 | 2022 | Canada | North America | West African clade |
| 194 | OP013007 | 2022 | Canada | North America | West African clade |
| 195 | OP012849 | 2022 | Taiwan | Asia | West African clade |
| 196 | DQ011154 | 2003 | Democratic Republic of the Congo | Africa | Congo Basin clade |
| 197 | ON880513 | 2022 | Canada | North America | West African clade |
| 198 | ON880516 | 2022 | Canada | North America | West African clade |
| 199 | ON880517 | 2022 | Canada | North America | West African clade |
| 200 | ON880539 | 2022 | Canada | North America | West African clade |
| 201 | ON880540 | 2022 | Canada | North America | West African clade |
| 202 | ON880542 | 2022 | Canada | North America | West African clade |
| 203 | ON880545 | 2022 | Canada | North America | West African clade |
| 204 | ON880546 | 2022 | Canada | North America | West African clade |
| 205 | ON880548 | 2022 | Canada | North America | West African clade |
| 206 | ON959143 | 2022 | Finland | Europe | West African clade |
| 207 | ON983163 | 2022 | Canada | North America | West African clade |
| 208 | ON983167 | 2022 | Canada | North America | West African clade |
| 209 | OP013004 | 2022 | Canada | North America | West African clade |
| 210 | OP013005 | 2022 | Canada | North America | West African clade |
| 211 | OP013008 | 2022 | Canada | North America | West African clade |
| 212 | OP013009 | 2022 | Canada | North America | West African clade |
| 213 | OP013010 | 2022 | Canada | North America | West African clade |
| 214 | OP013013 | 2022 | Canada | North America | West African clade |
| 215 | OP013014 | 2022 | Canada | North America | West African clade |
| 216 | OP013015 | 2022 | Canada | North America | West African clade |
| 217 | ON880511 | 2022 | Canada | North America | West African clade |
| 218 | ON880526 | 2022 | Canada | North America | West African clade |
| 219 | ON880527 | 2022 | Canada | North America | West African clade |
| 220 | ON880530 | 2022 | Canada | North America | West African clade |
| 221 | ON880534 | 2022 | Canada | North America | West African clade |
| 222 | ON880536 | 2022 | Canada | North America | West African clade |
| 223 | ON880547 | 2022 | Canada | North America | West African clade |
| 224 | ON983162 | 2022 | Canada | North America | West African clade |
| 225 | ON983164 | 2022 | Canada | North America | West African clade |
| 226 | ON649714 | 2022 | Portugal | Europe | West African clade |
| 227 | ON649716 | 2022 | Portugal | Europe | West African clade |
| 228 | ON782021 | 2022 | Finland | Europe | West African clade |
| 229 | ON880538 | 2022 | Canada | North America | West African clade |
| 230 | ON880543 | 2022 | Canada | North America | West African clade |
| 231 | OP013011 | 2022 | Canada | North America | West African clade |
| 232 | OP019276 | 2022 | Austria | Europe | West African clade |
| 233 | OP019277 | 2022 | Austria | Europe | West African clade |
| 234 | ON880505 | 2022 | Canada | North America | West African clade |
| 235 | ON880518 | 2022 | Canada | North America | West African clade |
| 236 | ON880519 | 2022 | Canada | North America | West African clade |
| 237 | ON880525 | 2022 | Canada | North America | West African clade |
| 238 | ON983166 | 2022 | Canada | North America | West African clade |
| 239 | ON649715 | 2022 | Portugal | Europe | West African clade |
| 240 | ON803416 | 2022 | Canada | North America | West African clade |
| 241 | ON983165 | 2022 | Canada | North America | West African clade |
| 242 | OP013016 | 2022 | Canada | North America | West African clade |
| 243 | ON880531 | 2022 | Canada | North America | West African clade |
| 244 | ON983160 | 2022 | Canada | North America | West African clade |
| 245 | ON803434 | 2022 | Canada | North America | West African clade |
| 246 | ON880507 | 2022 | Canada | North America | West African clade |
| 247 | ON927243 | 2022 | Spain | Europe | West African clade |
| 248 | ON649711 | 2022 | Portugal | Europe | West African clade |
| 249 | ON803433 | 2022 | Canada | North America | West African clade |
| 250 | ON838939 | 2022 | Spain | Europe | West African clade |
| 251 | ON803415 | 2022 | Canada | North America | West African clade |
| 252 | ON880512 | 2022 | Canada | North America | West African clade |
| 253 | ON803436 | 2022 | Canada | North America | West African clade |
| 254 | ON803444 | 2022 | Canada | North America | West African clade |
| 255 | ON880522 | 2022 | Canada | North America | West African clade |
| 256 | ON619837 | 2022 | United Kingdom | Europe | West African clade |
| 257 | OP022171 | 2022 | United Kingdom | Europe | West African clade |
| 258 | ON694342 | 2022 | Germany | Europe | West African clade |
| 259 | ON792320 | 2022 | Switzerland | Europe | West African clade |
| 260 | ON950045 | 2022 | Belgium | Europe | West African clade |
| 261 | ON676704 | 2022 | USA | North America | West African clade |
| 262 | ON782022 | 2022 | Finland | Europe | West African clade |
| 263 | OP013001 | 2022 | Canada | North America | West African clade |
| 264 | ON880523 | 2022 | Canada | North America | West African clade |
| 265 | ON676708 | 2021 | USA | North America | West African clade |
| 266 | ON880508 | 2022 | Canada | North America | West African clade |
| 267 | ON880521 | 2022 | Canada | North America | West African clade |
| 268 | ON674051 | 2022 | USA | North America | West African clade |
| 269 | ON838940 | 2022 | Spain | Europe | West African clade |
| 270 | ON880509 | 2022 | Canada | North America | West African clade |
| 271 | ON880510 | 2022 | Canada | North America | West African clade |
| 272 | ON676705 | 2022 | USA | North America | West African clade |
| 273 | JX878425 | 2007 | Democratic Republic of the Congo | Africa | Congo Basin clade |
| 274 | ON813252 | 2022 | Germany | Europe | West African clade |
| 275 | ON880520 | 2022 | Canada | North America | West African clade |
| 276 | ON880506 | 2022 | Canada | North America | West African clade |
| 277 | ON929077 | 2022 | Germany | Europe | West African clade |
| 278 | ON929060 | 2022 | Germany | Europe | West African clade |
| 279 | ON929062 | 2022 | Germany | Europe | West African clade |
| 280 | ON929078 | 2022 | Germany | Europe | West African clade |
| 281 | ON929090 | 2022 | Germany | Europe | West African clade |
| 282 | OP018600 | 2022 | Germany | Europe | West African clade |
| 283 | OP018601 | 2022 | Germany | Europe | West African clade |
| 284 | OP018603 | 2022 | Germany | Europe | West African clade |
| 285 | ON682263 | 2022 | Germany | Europe | West African clade |
| 286 | ON682265 | 2022 | Germany | Europe | West African clade |
| 287 | ON682266 | 2022 | Germany | Europe | West African clade |
| 288 | ON682267 | 2022 | Germany | Europe | West African clade |
| 289 | ON682268 | 2022 | Germany | Europe | West African clade |
| 290 | ON682269 | 2022 | Germany | Europe | West African clade |
| 291 | ON682270 | 2022 | Germany | Europe | West African clade |
| 292 | ON694341 | 2022 | Germany | Europe | West African clade |
| 293 | ON755231 | 2022 | Germany | Europe | West African clade |
| 294 | ON755232 | 2022 | Germany | Europe | West African clade |
| 295 | ON755233 | 2022 | Germany | Europe | West African clade |
| 296 | ON755234 | 2022 | Germany | Europe | West African clade |
| 297 | ON755235 | 2022 | Germany | Europe | West African clade |
| 298 | ON755236 | 2022 | Germany | Europe | West African clade |
| 299 | ON755237 | 2022 | Germany | Europe | West African clade |
| 300 | ON755238 | 2022 | Germany | Europe | West African clade |
| 301 | ON755239 | 2022 | Germany | Europe | West African clade |
| 302 | ON755240 | 2022 | Germany | Europe | West African clade |
| 303 | ON755241 | 2022 | Germany | Europe | West African clade |
| 304 | ON755242 | 2022 | Germany | Europe | West African clade |
| 305 | ON755243 | 2022 | Germany | Europe | West African clade |
| 306 | ON755244 | 2022 | Germany | Europe | West African clade |
| 307 | ON755245 | 2022 | Germany | Europe | West African clade |
| 308 | ON755246 | 2022 | Germany | Europe | West African clade |
| 309 | ON755247 | 2022 | Germany | Europe | West African clade |
| 310 | ON755248 | 2022 | Germany | Europe | West African clade |
| 311 | ON755249 | 2022 | Germany | Europe | West African clade |
| 312 | ON755250 | 2022 | Germany | Europe | West African clade |
| 313 | ON755251 | 2022 | Germany | Europe | West African clade |
| 314 | ON755252 | 2022 | Germany | Europe | West African clade |
| 315 | ON755253 | 2022 | Germany | Europe | West African clade |
| 316 | ON755254 | 2022 | Germany | Europe | West African clade |
| 317 | ON755255 | 2022 | Germany | Europe | West African clade |
| 318 | ON755256 | 2022 | Germany | Europe | West African clade |
| 319 | ON803428 | 2022 | Canada | North America | West African clade |
| 320 | ON813251 | 2022 | Germany | Europe | West African clade |
| 321 | ON813253 | 2022 | Germany | Europe | West African clade |
| 322 | ON813254 | 2022 | Germany | Europe | West African clade |
| 323 | ON813255 | 2022 | Germany | Europe | West African clade |
| 324 | ON813256 | 2022 | Germany | Europe | West African clade |
| 325 | ON813257 | 2022 | Germany | Europe | West African clade |
| 326 | ON813258 | 2022 | Germany | Europe | West African clade |
| 327 | ON813259 | 2022 | Germany | Europe | West African clade |
| 328 | ON813260 | 2022 | Germany | Europe | West African clade |
| 329 | ON813261 | 2022 | Germany | Europe | West African clade |
| 330 | ON813262 | 2022 | Germany | Europe | West African clade |
| 331 | ON813264 | 2022 | Germany | Europe | West African clade |
| 332 | ON813265 | 2022 | Germany | Europe | West African clade |
| 333 | ON813266 | 2022 | Germany | Europe | West African clade |
| 334 | ON813267 | 2022 | Germany | Europe | West African clade |
| 335 | ON853649 | 2022 | Germany | Europe | West African clade |
| 336 | ON853650 | 2022 | Germany | Europe | West African clade |
| 337 | ON853651 | 2022 | Germany | Europe | West African clade |
| 338 | ON853652 | 2022 | Germany | Europe | West African clade |
| 339 | ON853653 | 2022 | Germany | Europe | West African clade |
| 340 | ON853654 | 2022 | Germany | Europe | West African clade |
| 341 | ON853655 | 2022 | Germany | Europe | West African clade |
| 342 | ON853656 | 2022 | Germany | Europe | West African clade |
| 343 | ON853657 | 2022 | Germany | Europe | West African clade |
| 344 | ON853658 | 2022 | Germany | Europe | West African clade |
| 345 | ON853659 | 2022 | Germany | Europe | West African clade |
| 346 | ON853660 | 2022 | Germany | Europe | West African clade |
| 347 | ON853661 | 2022 | Germany | Europe | West African clade |
| 348 | ON853662 | 2022 | Germany | Europe | West African clade |
| 349 | ON853663 | 2022 | Germany | Europe | West African clade |
| 350 | ON853664 | 2022 | Germany | Europe | West African clade |
| 351 | ON853665 | 2022 | Germany | Europe | West African clade |
| 352 | ON853666 | 2022 | Germany | Europe | West African clade |
| 353 | ON853667 | 2022 | Germany | Europe | West African clade |
| 354 | ON853668 | 2022 | Germany | Europe | West African clade |
| 355 | ON853669 | 2022 | Germany | Europe | West African clade |
| 356 | ON853670 | 2022 | Germany | Europe | West African clade |
| 357 | ON853671 | 2022 | Germany | Europe | West African clade |
| 358 | ON853672 | 2022 | Germany | Europe | West African clade |
| 359 | ON853673 | 2022 | Germany | Europe | West African clade |
| 360 | ON853674 | 2022 | Germany | Europe | West African clade |
| 361 | ON853675 | 2022 | Germany | Europe | West African clade |
| 362 | ON853676 | 2022 | Germany | Europe | West African clade |
| 363 | ON853677 | 2022 | Germany | Europe | West African clade |
| 364 | ON853678 | 2022 | Germany | Europe | West African clade |
| 365 | ON929057 | 2022 | Germany | Europe | West African clade |
| 366 | ON929059 | 2022 | Germany | Europe | West African clade |
| 367 | ON929061 | 2022 | Germany | Europe | West African clade |
| 368 | ON929063 | 2022 | Germany | Europe | West African clade |
| 369 | ON929066 | 2022 | Germany | Europe | West African clade |
| 370 | ON929067 | 2022 | Germany | Europe | West African clade |
| 371 | ON929069 | 2022 | Germany | Europe | West African clade |
| 372 | ON929073 | 2022 | Germany | Europe | West African clade |
| 373 | ON929079 | 2022 | Germany | Europe | West African clade |
| 374 | ON929084 | 2022 | Germany | Europe | West African clade |
| 375 | ON929086 | 2022 | Germany | Europe | West African clade |
| 376 | ON929087 | 2022 | Germany | Europe | West African clade |
| 377 | ON929089 | 2022 | Germany | Europe | West African clade |
| 378 | OP018588 | 2022 | Germany | Europe | West African clade |
| 379 | OP018589 | 2022 | Germany | Europe | West African clade |
| 380 | OP018590 | 2022 | Germany | Europe | West African clade |
| 381 | OP018592 | 2022 | Germany | Europe | West African clade |
| 382 | OP018593 | 2022 | Germany | Europe | West African clade |
| 383 | OP018594 | 2022 | Germany | Europe | West African clade |
| 384 | OP018595 | 2022 | Germany | Europe | West African clade |
| 385 | OP018596 | 2022 | Germany | Europe | West African clade |
| 386 | OP018598 | 2022 | Germany | Europe | West African clade |
| 387 | OP018602 | 2022 | Germany | Europe | West African clade |
| 388 | OP018604 | 2022 | Germany | Europe | West African clade |
| 389 | OP018605 | 2022 | Germany | Europe | West African clade |
| 390 | OP018607 | 2022 | Germany | Europe | West African clade |
| 391 | ON813263 | 2022 | Germany | Europe | West African clade |
| 392 | ON929058 | 2022 | Germany | Europe | West African clade |
| 393 | ON929064 | 2022 | Germany | Europe | West African clade |
| 394 | ON929065 | 2022 | Germany | Europe | West African clade |
| 395 | ON929068 | 2022 | Germany | Europe | West African clade |
| 396 | ON929070 | 2022 | Germany | Europe | West African clade |
| 397 | ON929071 | 2022 | Germany | Europe | West African clade |
| 398 | ON929072 | 2022 | Germany | Europe | West African clade |
| 399 | ON929074 | 2022 | Germany | Europe | West African clade |
| 400 | ON929075 | 2022 | Germany | Europe | West African clade |
| 401 | ON929076 | 2022 | Germany | Europe | West African clade |
| 402 | ON929080 | 2022 | Germany | Europe | West African clade |
| 403 | ON929081 | 2022 | Germany | Europe | West African clade |
| 404 | ON929082 | 2022 | Germany | Europe | West African clade |
| 405 | ON929083 | 2022 | Germany | Europe | West African clade |
| 406 | ON929085 | 2022 | Germany | Europe | West African clade |
| 407 | ON929088 | 2022 | Germany | Europe | West African clade |
| 408 | ON929091 | 2022 | Germany | Europe | West African clade |
| 409 | ON959149 | 2022 | Germany | Europe | West African clade |
| 410 | ON959150 | 2022 | Germany | Europe | West African clade |
| 411 | ON959151 | 2022 | Germany | Europe | West African clade |
| 412 | ON959152 | 2022 | Germany | Europe | West African clade |
| 413 | ON959153 | 2022 | Germany | Europe | West African clade |
| 414 | ON959154 | 2022 | Germany | Europe | West African clade |
| 415 | ON959155 | 2022 | Germany | Europe | West African clade |
| 416 | ON959156 | 2022 | Germany | Europe | West African clade |
| 417 | ON959157 | 2022 | Germany | Europe | West African clade |
| 418 | ON959158 | 2022 | Germany | Europe | West African clade |
| 419 | ON959159 | 2022 | Germany | Europe | West African clade |
| 420 | ON959160 | 2022 | Germany | Europe | West African clade |
| 421 | ON959161 | 2022 | Germany | Europe | West African clade |
| 422 | ON959163 | 2022 | Germany | Europe | West African clade |
| 423 | ON959165 | 2022 | Germany | Europe | West African clade |
| 424 | ON959166 | 2022 | Germany | Europe | West African clade |
| 425 | ON959167 | 2022 | Germany | Europe | West African clade |
| 426 | ON959168 | 2022 | Germany | Europe | West African clade |
| 427 | ON959169 | 2022 | Germany | Europe | West African clade |
| 428 | ON959170 | 2022 | Germany | Europe | West African clade |
| 429 | ON959171 | 2022 | Germany | Europe | West African clade |
| 430 | ON959172 | 2022 | Germany | Europe | West African clade |
| 431 | ON959173 | 2022 | Germany | Europe | West African clade |
| 432 | ON959174 | 2022 | Germany | Europe | West African clade |
| 433 | ON959175 | 2022 | Germany | Europe | West African clade |
| 434 | ON959176 | 2022 | Germany | Europe | West African clade |
| 435 | ON959177 | 2022 | Germany | Europe | West African clade |
| 436 | OP018591 | 2022 | Germany | Europe | West African clade |
| 437 | OP018597 | 2022 | Germany | Europe | West African clade |
| 438 | OP018599 | 2022 | Germany | Europe | West African clade |
| 439 | OP018606 | 2022 | Germany | Europe | West African clade |
| 440 | ON959162 | 2022 | Germany | Europe | West African clade |
| 441 | ON959164 | 2022 | Germany | Europe | West African clade |
| 442 | ON853679 | 2022 | Germany | Europe | West African clade |
| 443 | ON853680 | 2022 | Germany | Europe | West African clade |
| 444 | MT903343 | 2018 | United Kingdom | Europe | West African clade |
| 445 | ON853682 | 2022 | Germany | Europe | West African clade |
| 446 | ON682264 | 2022 | Germany | Europe | West African clade |
| 447 | ON637938 | 2022 | Germany | Europe | West African clade |
| 448 | ON637939 | 2022 | Germany | Europe | West African clade |
| 449 | ON808413 | 2022 | United Kingdom | Europe | West African clade |
| 450 | ON808414 | 2022 | United Kingdom | Europe | West African clade |
| 451 | ON808415 | 2022 | United Kingdom | Europe | West African clade |
| 452 | ON808416 | 2022 | United Kingdom | Europe | West African clade |
| 453 | ON808417 | 2022 | United Kingdom | Europe | West African clade |
| 454 | ON853681 | 2022 | Germany | Europe | West African clade |
| 455 | ON622720 | 2022 | Switzerland | Europe | West African clade |
| 456 | ON622722 | 2022 | France | Europe | West African clade |
| 457 | ON627808 | 2022 | USA | North America | West African clade |
| 458 | ON602722 | 2022 | France | Europe | West African clade |
| 459 | ON676703 | 2022 | USA | North America | West African clade |
| 460 | ON720849 | 2022 | Spain | Europe | West African clade |
| 461 | ON694332 | 2022 | Germany | Europe | West African clade |
| 462 | ON585029 | 2022 | Portugal | Europe | West African clade |
| 463 | ON872184 | 2022 | Ireland | Europe | West African clade |
| 464 | ON792321 | 2022 | Switzerland | Europe | West African clade |
| 465 | ON595760 | 2022 | Switzerland | Europe | West African clade |
| 466 | ON720848 | 2022 | Spain | Europe | West African clade |
| 467 | ON983159 | 2022 | Canada | North America | West African clade |
| 468 | ON676706 | 2022 | USA | North America | West African clade |
| 469 | HM172544 | 1979 | Zaire | Africa | Congo Basin clade |
| 470 | ON755040 | 2022 | France | Europe | West African clade |
| 471 | ON675438 | 2022 | USA | North America | West African clade |
| 472 | ON622721 | 2022 | Italy | Europe | West African clade |
| 473 | JX878429 | 2007 | Democratic Republic of the Congo | Africa | Congo Basin clade |
| 474 | NC_003310 | 1996 | - | Africa | Congo Basin clade |
| 475 | HQ857562 | 1979 | Zaire | Africa | Congo Basin clade |
| 476 | ON649879 | 2022 | Israel | Asia | West African clade |
| 477 | JX878426 | 2007 | Democratic Republic of the Congo | Africa | Congo Basin clade |
| 478 | JX878408 | 2006 | Democratic Republic of the Congo | Africa | Congo Basin clade |
| 479 | JX878418 | 2007 | Democratic Republic of the Congo | Africa | Congo Basin clade |
| 480 | JX878419 | 2007 | Democratic Republic of the Congo | Africa | Congo Basin clade |
| 481 | JX878420 | 2007 | Democratic Republic of the Congo | Africa | Congo Basin clade |
| 482 | JX878423 | 2007 | Democratic Republic of the Congo | Africa | Congo Basin clade |
| 483 | KJ642619 | 1988 | Gabon | Africa | Congo Basin clade |
| 484 | ON615424 | 2022 | Netherlands | Europe | West African clade |
| 485 | KC257460 | 1985 | Democratic Republic of the Congo | Africa | Congo Basin clade |
| 486 | KJ642613 | 1970 | Zaire | Africa | Congo Basin clade |
| 487 | JX878407 | 2006 | Democratic Republic of the Congo | Africa | Congo Basin clade |
| 488 | ON622718 | 2022 | Spain | Europe | West African clade |
| 489 | ON694335 | 2022 | Germany | Europe | West African clade |
| 490 | JX878424 | 2007 | Democratic Republic of the Congo | Africa | Congo Basin clade |
| 491 | ON843182 | 2022 | Portugal | Europe | West African clade |
| 492 | ON843181 | 2022 | Portugal | Europe | West African clade |
| 493 | ON585038 | 2022 | Portugal | Europe | West African clade |
| 494 | ON585037 | 2022 | Portugal | Europe | West African clade |
| 495 | ON649713 | 2022 | Portugal | Europe | West African clade |
| 496 | ON755039 | 2022 | France | Europe | West African clade |
| 497 | ON983168 | 2022 | Czech Republic | Europe | West African clade |
| 498 | KJ642612 | 1986 | Zaire | Africa | Congo Basin clade |
| 499 | KJ642618 | 1990 | Cameroon | Africa | Congo Basin clade |
| 500 | MN346693 | 2017 | Cote d'Ivoire | Africa | West African clade |
| 501 | MT724770 | 2014 | Democratic Republic of the Congo | Africa | Congo Basin clade |
| 502 | ON782054 | 2022 | Spain | Europe | West African clade |
| 503 | MT724772 | 2014 | Democratic Republic of the Congo | Africa | Congo Basin clade |
| 504 | MN702445 | 2017 | Central African Republic | Africa | Congo Basin clade |
| 505 | MN702451 | 2017 | Central African Republic | Africa | Congo Basin clade |
| 506 | MN702446 | 2018 | Central African Republic | Africa | Congo Basin clade |
| 507 | MT724771 | 2014 | Democratic Republic of the Congo | Africa | Congo Basin clade |
| 508 | ON614676 | 2022 | Italy | Europe | West African clade |
| 509 | MN702449 | 2016 | Central African Republic | Africa | Congo Basin clade |
| 510 | MN702450 | 2016 | Central African Republic | Africa | Congo Basin clade |
| 511 | MN702452 | 2010 | Central African Republic | Africa | Congo Basin clade |
| 512 | MT724769 | 2012 | Democratic Republic of the Congo | Africa | West African clade |
| 513 | MN702444 | 2017 | Central African Republic | Africa | Congo Basin clade |
| 514 | KJ642614 | 1965 | Netherlands | Europe | West African clade |
| 515 | MN702447 | 2018 | Central African Republic | Africa | Congo Basin clade |
| 516 | MN702448 | 2018 | Central African Republic | Africa | Congo Basin clade |
| 517 | MN702453 | 2001 | Central African Republic | Africa | Congo Basin clade |
| 518 | OL504743 | 2019 | United Kingdom | Europe | West African clade |
| 519 | OL504742 | 2019 | United Kingdom | Europe | West African clade |
| 520 | OL504741 | 2019 | United Kingdom | Europe | West African clade |
| 521 | MT903341 | 2018 | Nigeria | Africa | West African clade |
| 522 | ON645312 | 2022 | United Kingdom | Europe | West African clade |
| 523 | MG693724 | 2017 | Nigeria | Africa | West African clade |
| 524 | MG693723 | 2017 | Nigeria | Africa | West African clade |
| 525 | MG693725 | 2017 | Nigeria | Africa | West African clade |

# Appendix Table S7: Source used to analysis the effective reproductive number for MPXV from 1990 to 2020

| **Clade** | **Country** | **Cases number infected by animal** | **Cases number infected by human to human** | **Year** | **Reference ID** |
| --- | --- | --- | --- | --- | --- |
| Congo Basin clade | Central African Republic | 1 | 9 | 2015 | 2 |
| West African clade | USA | 9 | 0 | 2003 | 7 |
| West African clade | Nigeria | 10 | 36 | 2017 | 23 |
| Congo Basin clade | Sudan | 1 | 9 | 2005 | 83 |
| Congo Basin clade | Sudan | 1 | 2 | 2005 | 83 |
| Congo Basin clade | Sudan | 1 | 1 | 2005 | 83 |
| Congo Basin clade | Sudan | 1 | 3 | 2005 | 83 |
| West African clade | USA | 20 | 7 | 2003 | 36 |
| Congo Basin clade | Central African Republic | 3 | 9 | 2015 | 11 |
| West African clade | Nigeria | 3 | 2 | 2017 | 19 |
| Congo Basin clade | Democratic Republic of the Congo | 1 | 14 | 2017 | 28 |
| Congo Basin clade | Democratic Republic of the Congo | 1 | 3 | 2017 | 28 |
| Congo Basin clade | Democratic Republic of the Congo | 92 | 327 | 1996 | 53 |
| Congo Basin clade | Congo | 1 | 10 | 2003 | 60 |
| Congo Basin clade | Democratic Republic of the Congo | 1 | 70 | 1996 | 61 |
| West African clade | USA | 35 | 52 | 2003 | 36 |

# Appendix Table S8: Definition of MPXV infection in potential reservoir hosts

| **Habitat type** | **Taxa** | **Common name** | **Species** | **Detection methods** | **Reference** |
| --- | --- | --- | --- | --- | --- |
| Terrestrial rodent | *Graphiurus* spp. | African dormouse | *Graphiurus lorraine, Graphiurus crassicaudatus* | PCR | 1. Hutson CL, Lee KN, Abel J, Carroll DS, Montgomery JM, Olson VA, et al. Monkeypox zoonotic associations: insights from laboratory evaluation of animals associated with the multi-state US outbreak. *Am J Trop Med Hyg*. 2007; 76(4): 757-768.  2. Fuller T, Thomassen HA, Mulembakani PM, Johnston SC, Lloyd-Smith JO, Kisalu NK, et al. Using remote sensing to map the risk of human monkeypox virus in the Congo Basin. *Ecohealth*. 2011;8(1):14-25. |
| Terrestrial rodent | *Cricetomys* spp. | Giant pouched rat | *Cricetomys gambianus* | PCR | 1. Hutson CL, Lee KN, Abel J, Carroll DS, Montgomery JM, Olson VA, et al. Monkeypox zoonotic associations: insights from laboratory evaluation of animals associated with the multi-state US outbreak. *Am J Trop Med Hyg*. 2007; 76(4): 757-768.  2. Falendysz EA, Lopera JG, Lorenzsonn F, Salzer JS, Hutson CL, Doty J, et al. Further assessment of Monkeypox virus infection in Gambian pouched rats (*Cricetomys gambianus*) using in vivo bioluminescent imaging. *PLoS Negl Trop Dis*. 2015; 9(10): e0004130.  3. Hutson CL, Nakazawa YJ, Self J, Olson VA, Regnery RL, Braden Z, et al. Laboratory investigations of african pouched rats (*Cricetomys gambianus*) as a potential reservoir host species for Monkeypox virus. *PLoS Negl Trop Dis*. 2015; 9(10): e0004013. |
| Arboreal rodent | *Funisciurus* spp. | Rope squirrel | *Funisciurus anerythrus*,  *Funisciurus carruthersi*,  *Funisciurus congicus*,  *Funisciurus lemniscatus*,  *Funisciurus pyrropus*,  *Funisciurus bayonii*,  *Funisciurus bayonii* | Virus Isolation/PCR | 1. Khodakevich L, Jezek Z, Kinzanzka K. Isolation of Monkeypox virus from wild squirrel infected in nature. *Lancet*. 1986; 1(8472): 98-99.  2. Hutson CL, Lee KN, Abel J, Carroll DS, Montgomery JM, Olson VA, et al. Monkeypox zoonotic associations: insights from laboratory evaluation of animals associated with the multi-state US outbreak. *Am J Trop Med Hyg*. 2007; 76(4): 757-768.  3. Reynolds MG, Carroll DS, Olson VA, Hughes C, Galley J, Likos A, et al. A silent enzootic of an orthopoxvirus in Ghana, West Africa: evidence for multi-species involvement in the absence of widespread human disease. *Am J Trop Med Hyg*. 2010; 82(4): 746-754.  4. Tiee MS, Harrigan RJ, Thomassen HA, Smith TB. Ghosts of infections past: using archival samples to understand a century of monkeypox virus prevalence among host communities across space and time. *R Soc Open Sci*. 2018; 5(1): 171089. |

# Appendix Table S9: Information of data source in the modeling analysis

| **Variables** | **Dataset** | **Temporal extent** | **Spatial resolution** | **Website** | **Reference** |
| --- | --- | --- | --- | --- | --- |
| Climate data | WorldClim | 1980-2018* | 0°2.5′ | <https://www.worldclim.org/> | Fick SE, Hijmans RJ. WorldClim 2: new 1-km spatial resolution climate surfaces for global land areas. *Int. J. Climatol.* 2017; **37**(12): 4302-15.  Harris I, Jones PD, Osborn TJ, Lister DH. Updated high-resolution grids of monthly climatic observations – the CRU TS3.10 Dataset. *Int. J. Climatol.* 2014; **34**(3): 623-42. |
| Elevation | EarthEnv-DEM90 digital elevation model | 2010 | 1km | http://www.earthenv.org/ | Robinson N, Regetz J, Guralnick RP. EarthEnv-DEM90: A nearly-global, void-free, multi-scale smoothed, 90m digital elevation model from fused ASTER and SRTM data. *ISPRS Journal of Photogrammetry and Remote Sensing.* 2014; **87**: 57-67. |
| Leaf area index | GLOBMAP Leaf Area Index (LAI) Version 3 | 1981-2019 | 8km | https://www.resdc.cn/ | Liu Y, Liu R, Chen JM. Retrospective retrieval of long-term consistent global leaf area index (1981–2011) from combined AVHRR and MODIS data. *J. Geophys. Res.* 2012; **117**(G4). |
| Land cover | The Land Cover CCI Climate Research Data Package (CRDP) | 1992-2019 | 0.3km | https://maps.elie.ucl.ac.be/CCI/ | ESA Land Cover CCI project team; Defourny, P. (2019): ESA Land Cover Climate Change Initiative (Land_Cover_cci): Global Land Cover Maps, Version 2.0.7. Centre for Environmental Data Analysis. |
| Richness of Rodentia/Primate | Diversity of Rodentia/Primate | 2018 | 10km | https://biodiversitymapping.org/index.php/mammals/ | Jenkins CN, Pimm SL, Joppa LN. Global patterns of terrestrial vertebrate diversity and conservation. *Proc Natl Acad Sci U S A* 2013; **110**(28): E2602-E10.  Pimm SL, Jenkins CN, Abell R, et al. The biodiversity of species and their rates of extinction, distribution, and protection. *Science.* 2014; **344**(6187): 1246752. |
| Mammalian richness | Global Mammal Richness Grids, 2015 Release (2013) | 2013 | 0°0′30″ | https://sedac.ciesin.columbia.edu/ | IUCN. Gridded Species Distribution: Global Mammal Richness Grids, 2015 Release. Palisades. 2015. https://sedac.ciesin.columbia.edu/ (accessed Jun 10, 2022). |
| Population count | Population counts / Unconstrained global mosaics 2000-2020 (1km resolution) | 2000-2020 | 1km | https://www.worldpop.org/ | WorldPop. Open Spatial Demographic Data and Research. 2018. https://www.worldpop.org/ (accessed Jun 10, 2022).  Olén NB, Lehsten V. High-resolution global population projections dataset developed with CMIP6 RCP and SSP scenarios for year 2010–2100. *Data in Brief.* 2022; **40**: 107804. |
| Global Downscaled GDP | Global 15 x 15 Minute Grids of the Downscaled GDP Based on the SRES B2 Scenario, v1 (1990, 2025) | 1990, 2025 | 0°15′ | https://sedac.ciesin.columbia.edu/ | Search EC. Global 15 x 15 Minute Grids of the Downscaled GDP Based on the SRES B2 Scenario, 1990 and 2025. 2022. https://sedac.ciesin.columbia.edu/ (accessed Jun 10, 2022). |
| Human Footprint | Human Footprint dataset from 2000 to 2018 | 2000-2018 | 1km | https://doi.org/10.6084/m9.figshare.16571064 | Mu H, Li X, Wen Y, et al. A global record of annual terrestrial Human Footprint dataset from 2000 to 2018. *Sci Data.* 2022; **9**(1): 176. |

*This dataset is recalculated according to historical monthly weather data for 1980-2018, by using the ‘biovars’ function in the R package “dismo”.

# Appendix Table S10: Description of 45 potential influencing factors used in the modelling efforts

| **Group** | **Variable** | **Description** | **Type** |
| --- | --- | --- | --- |
| Environment |  |  |  |
|  | BIO1 | Annual mean temperature (℃) | Continuous |
|  | BIO2 | Mean diurnal range (Mean of monthly (max temp-min temp)) (℃) | Continuous |
|  | BIO3 | Isothermality (BIO2/ BIO7) (*100) | Continuous |
|  | BIO4 | Temperature seasonality (standard deviation*100) | Continuous |
|  | BIO5 | Max temperature of warmest month (℃) | Continuous |
|  | BIO6 | Min temperature of coldest month (℃) | Continuous |
|  | BIO7 | Annual range of temperature (BIO5- BIO6) (℃) | Continuous |
|  | BIO8 | Mean temperature of wettest quarter (℃) | Continuous |
|  | BIO9 | Mean temperature of driest quarter (℃) | Continuous |
|  | BIO10 | Mean temperature of warmest quarter (℃) | Continuous |
|  | BIO11 | Mean temperature of coldest quarter (℃) | Continuous |
|  | BIO12 | Annual precipitation (mm) | Continuous |
|  | BIO13 | Precipitation of wettest month (mm) | Continuous |
|  | BIO14 | Precipitation of driest month (mm) | Continuous |
|  | BIO15 | Precipitation seasonality (Coefficient of variation) | Continuous |
|  | BIO16 | Precipitation of wettest quarter (mm) | Continuous |
|  | BIO17 | Precipitation of driest quarter (mm) | Continuous |
|  | BIO18 | Precipitation of warmest quarter (mm) | Continuous |
|  | BIO19 | Precipitation of coldest quarter (mm) | Continuous |
|  | Leaf area index | Area of leaves (m²) over a unit of land (m²) | Continuous |
|  | Elevation | Average elevation (m) | Continuous |
|  | Latitude | Latitude (°) | Continuous |
|  | Mixed cropland and nature vegetation | Percentage coverage of mixed cropland and nature vegetation (1%) | Continuous |
|  | Forest | Percentage coverage of forest (1%) | Continuous |
|  | Shrubland | Percentage coverage of shrubland (1%) | Continuous |
|  | Mixed tree, shrub and herbaceous | Percentage coverage of mixed tree, shrub and herbaceous (1%) | Continuous |
|  | Grassland | Percentage coverage of grassland (1%) | Continuous |
|  | Sparse vegetation land | Percentage coverage of sparse vegetation land (1%) | Continuous |
|  | Flooded vegetation | Percentage coverage of flooded vegetation (1%) | Continuous |
|  | Bare areas | Percentage coverage of bare areas (1%) | Continuous |
|  | Water body | Percentage coverage of inland water body (1%) | Continuous |
| Human activity |  |  |  |
|  | Cropland | Percentage coverage of cropland (1%) | Continuous |
|  | Urban construction land | Percentage coverage of urban construction land (1%) | Continuous |
|  | Population count | Estimated total number of people per grid-cell | Continuous |
|  | Global Downscaled GDP | Geospatial distributions of GDP per unit area | Continuous |
|  | Human footprint | Annual records of the global human footprint (0-50) | Continuous |
| Animal |  |  |  |
|  | Rodentia richness | The number of Rodentia species per km² | Continuous |
|  | Primate richness | The number of Primate species per km² | Continuous |
|  | Mammalian richness | The number of mammal species per km² | Continuous |

# Appendix Table S11: Ecological factors potentially associated with the MPXV zoonotic transmission used in the modelling analysis

| **Variable** | **Reference** | **Content** | **Method** | **Usage and/or result** |
| --- | --- | --- | --- | --- |
| BIO1 | Arotolu TE, et al. [1] | Monkeypox disease in southern Nigeria | Model: MaxEnt | Used in the monkeypox ecological niche model |
|  | Curaudeau M, et al. [2] | MPXV in Africa | Model: MaxEnt | Used in the MPXV ecological niche model |
|  | Fuller T, et.al. [3] | Monkeypox disease in the Congo Basin | Model: Logistic regression and MaxEnt | Used in the monkeypox ecological niche model |
|  | Levine RS, et al. [4] | Monkeypox disease in Africa | Model: GARP | The variable has significant contributions to the transmission of monkeypox disease |
|  | Mandja BA, et.al. [5] | Monkeypox disease in the Democratic Republic of the Congo | Model: Bayesian hierarchical generalized linear mixed model | The IRR of the variable is 1.143 (1.028‒1.261) in the model |
|  | Ellis CK, et.al. [6] | Monkeypox disease in Africa | Model: MaxEnt | Significant environmental variable |
|  | Lash RR, et.al. [7] | Monkeypox disease in Africa | Model: GARP | Used in the monkeypox ecological niche model |
|  | Thomassen HA, et.al. [8] | Monkeypox disease in Central Africa | Model: MaxEnt | Used in the MPXV reservoir species model |
| BIO2 | Arotolu TE, et al. [1] | Monkeypox disease in southern Nigeria | Model: MaxEnt | Used in the monkeypox ecological niche model |
|  | Curaudeau M, et al. [2] | MPXV in Africa | Model: MaxEnt | Used in the MPXV ecological niche model |
|  | Fuller T, et.al. [3] | Monkeypox disease in the Congo Basin | Model: Logistic regression and MaxEnt | Used in the monkeypox ecological niche model |
|  | Levine RS, et al. [4] | Monkeypox disease in Africa | Model: GARP | The variable has significant contributions to the transmission of monkeypox disease |
|  | Ellis CK, et.al. [6] | Monkeypox disease in Africa | Model: MaxEnt | Significant environmental variable |
|  | Lash RR, et.al. [7] | Monkeypox disease in Africa | Model: GARP | Used in the monkeypox ecological niche model |
|  | Thomassen HA, et.al. [8] | Monkeypox disease in Central Africa | Model: MaxEnt | Used in the MPXV reservoir species model |
| BIO3 | Arotolu TE, et al. [1] | Monkeypox disease in southern Nigeria | Model: MaxEnt | Used in the monkeypox ecological niche model |
|  | Curaudeau M, et al. [2] | MPXV in Africa | Model: MaxEnt | Used in the MPXV ecological niche model |
|  | Fuller T, et.al. [3] | Monkeypox disease in the Congo Basin | Model: Logistic regression and MaxEnt | Used in the monkeypox ecological niche model |
|  | Ellis CK, et.al. [6] | Monkeypox disease in Africa | Model: MaxEnt | Used in the monkeypox ecological niche model |
| BIO4 | Arotolu TE, et al. [1] | Monkeypox disease in southern Nigeria | Model: MaxEnt | Used in the monkeypox ecological niche model |
|  | Curaudeau M, et al. [2] | MPXV in Africa | Model: MaxEnt | Used in the MPXV ecological niche model |
|  | Fuller T, et.al. [3] | Monkeypox disease in the Congo Basin | Model: Logistic regression and MaxEnt | Used in the monkeypox ecological niche model |
|  | Ellis CK, et.al. [6] | Monkeypox disease in Africa | Model: MaxEnt | Used in the monkeypox ecological niche model |
|  | Thomassen HA, et.al. [8] | Monkeypox disease in Central Africa | Model: MaxEnt | Used in the MPXV reservoir species model |
| BIO5 | Arotolu TE, et al. [1] | Monkeypox disease in southern Nigeria | Model: MaxEnt | Used in the monkeypox ecological niche model |
|  | Curaudeau M, et al. [2] | MPXV in Africa | Model: MaxEnt | Used in the MPXV ecological niche model |
|  | Fuller T, et.al. [3] | Monkeypox disease in the Congo Basin | Model: Logistic regression and MaxEnt | Used in the monkeypox ecological niche model |
|  | Ellis CK, et.al. [6] | Monkeypox disease in Africa | Model: MaxEnt | Significant environmental variable |
|  | Lash RR, et.al. [7] | Monkeypox disease in Africa | Model: GARP | Used in the monkeypox ecological niche model |
|  | Thomassen HA, et.al. [8] | Monkeypox disease in Central Africa | Model: MaxEnt | Used in the MPXV reservoir species model |
| BIO6 | Arotolu TE, et al. [1] | Monkeypox disease in southern Nigeria | Model: MaxEnt | Used in the monkeypox ecological niche model |
|  | Curaudeau M, et al. [2] | MPXV in Africa | Model: MaxEnt | Used in the MPXV ecological niche model |
|  | Fuller T, et.al. [3] | Monkeypox disease in the Congo Basin | Model: Logistic regression and MaxEnt | Used in the monkeypox ecological niche model |
|  | Ellis CK, et.al. [6] | Monkeypox disease in Africa | Model: MaxEnt | Significant environmental variable |
|  | Lash RR, et.al. [7] | Monkeypox disease in Africa | Model: GARP | Used in the monkeypox ecological niche model |
| BIO7 | Arotolu TE, et al. [1] | Monkeypox disease in southern Nigeria | Model: MaxEnt | Used in the monkeypox ecological niche model |
|  | Curaudeau M, et al. [2] | MPXV in Africa | Model: MaxEnt | Used in the MPXV ecological niche model |
|  | Fuller T, et.al. [3] | Monkeypox disease in the Congo Basin | Model: Logistic regression and MaxEnt | Used in the monkeypox ecological niche model |
|  | Ellis CK, et.al. [6] | Monkeypox disease in Africa | Model: MaxEnt | Used in the monkeypox ecological niche model |
| BIO8 | Arotolu TE, et al. [1] | Monkeypox disease in southern Nigeria | Model: MaxEnt | Used in the monkeypox ecological niche model |
|  | Curaudeau M, et al. [2] | MPXV in Africa | Model: MaxEnt | Used in the MPXV ecological niche mode. |
|  | Fuller T, et.al. [3] | Monkeypox disease in the Congo Basin | Model: Logistic regression and MaxEnt | Used in the monkeypox ecological niche model |
|  | Ellis CK, et.al. [6] | Monkeypox disease in Africa | Model: MaxEnt | Used in the monkeypox ecological niche model |
| BIO9 | Arotolu TE, et al. [1] | Monkeypox disease in southern Nigeria | Model: MaxEnt | Used in the monkeypox ecological niche model |
|  | Curaudeau M, et al. [2] | MPXV in Africa | Model: MaxEnt | Used in the monkeypox ecological niche model |
|  | Fuller T, et.al. [3] | Monkeypox disease in the Congo Basin | Model: Logistic regression and MaxEnt | Used in the monkeypox ecological niche model |
|  | Ellis CK, et.al. [6] | Monkeypox disease in Africa | Model: MaxEnt | Used in the monkeypox ecological niche model |
| BIO10 | Arotolu TE, et al. [1] | Monkeypox disease in southern Nigeria | Model: MaxEnt | Used in the monkeypox ecological niche model |
|  | Curaudeau M, et al. [2] | MPXV in Africa | Model: MaxEnt | Used in the MPXV ecological niche model |
|  | Fuller T, et.al. [3] | Monkeypox disease in the Congo Basin | Model: Logistic regression and MaxEnt | Used in the monkeypox ecological niche model |
|  | Ellis CK, et.al. [6] | Monkeypox disease in Africa | Model: MaxEnt | Used in the monkeypox ecological niche model |
| BIO11 | Arotolu TE, et al. [1] | Monkeypox disease in southern Nigeria | Model: MaxEnt | Used in the monkeypox ecological niche model |
|  | Curaudeau M, et al. [2] | MPXV in Africa | Model: MaxEnt | Used in the MPXV ecological niche model |
|  | Fuller T, et.al. [3] | Monkeypox disease in the Congo Basin | Model: Logistic regression and MaxEnt | Used in the monkeypox ecological niche model |
|  | Ellis CK, et.al. [6] | Monkeypox disease in Africa | Model: MaxEnt | Used in the monkeypox ecological niche model |
| BIO12 | Arotolu TE, et al. [1] | Monkeypox disease in southern Nigeria | Model: MaxEnt | Used in the monkeypox ecological niche model |
|  | Curaudeau M, et al. [2] | MPXV in Africa | Model: MaxEnt | Used in the MPXV ecological niche model |
|  | Fuller T, et.al. [3] | Monkeypox disease in the Congo Basin | Model: Logistic regression and MaxEnt | Used in the monkeypox ecological niche model |
|  | Levine RS, et al. [4] | Monkeypox disease in Africa | Model: GARP | The key environmental variable |
|  | Ellis CK, et.al. [6] | Monkeypox disease in Africa | Model: MaxEnt | Significant environmental variable |
|  | Lash RR, et.al. [7] | Monkeypox disease in Africa | Model: GARP | Used in the monkeypox ecological niche model |
|  | Thomassen HA, et.al. [8] | Monkeypox disease in Central Africa | Model: MaxEnt | Used in the MPXV reservoir species model |
| BIO13 | Arotolu TE, et al. [1] | Monkeypox disease in southern Nigeria | Model: MaxEnt | Used in the monkeypox ecological niche model |
|  | Curaudeau M, et al. [2] | MPXV in Africa | Model: MaxEnt | Used in the MPXV ecological niche model |
|  | Fuller T, et.al. [3] | Monkeypox disease in the Congo Basin | Model: Logistic regression and MaxEnt | Used in the monkeypox ecological niche model |
|  | Ellis CK, et.al. [6] | Monkeypox disease in Africa | Model: MaxEnt | Used in the monkeypox ecological niche model |
|  | Lash RR, et.al. [7] | Monkeypox disease in Africa | Model: GARP | Used in the monkeypox ecological niche model |
| BIO14 | Arotolu TE, et al. [1] | Monkeypox disease in southern Nigeria | Model: MaxEnt | Used in the monkeypox ecological niche model |
|  | Curaudeau M, et al. [2] | MPXV in Africa | Model: MaxEnt | Used in the MPXV ecological niche model |
|  | Fuller T, et.al. [3] | Monkeypox disease in the Congo Basin | Model: Logistic regression and MaxEnt | Used in the monkeypox ecological niche model |
|  | Ellis CK, et.al. [6] | Monkeypox disease in Africa | Model: MaxEnt | Used in the monkeypox ecological niche model |
|  | Lash RR, et.al. [7] | Monkeypox disease in Africa | Model: GARP | Used in the MPXV reservoir species model |
| BIO15 | Arotolu TE, et al. [1] | Monkeypox disease in southern Nigeria | Model: MaxEnt | Used in the monkeypox ecological niche model |
|  | Curaudeau M, et al. [2] | MPXV in Africa | Model: MaxEnt | Used in the MPXV ecological niche model |
|  | Fuller T, et.al. [3] | Monkeypox disease in the Congo Basin | Model: Logistic regression and MaxEnt | Used in the monkeypox ecological niche model |
|  | Ellis CK, et.al. [6] | Monkeypox disease in Africa | Model: MaxEnt | Used in the monkeypox ecological niche model |
| BIO16 | Arotolu TE, et al. [1] | Monkeypox disease in southern Nigeria | Model: MaxEnt | Used in the monkeypox ecological niche model |
|  | Curaudeau M, et al. [2] | MPXV in Africa | Model: MaxEnt | Used in the MPXV ecological niche model |
|  | Fuller T, et.al. [3] | Monkeypox disease in the Congo Basin | Model: Logistic regression and MaxEnt | Used in the monkeypox ecological niche model |
|  | Thomassen HA, et.al. [8] | Monkeypox disease in Central Africa | Model: MaxEnt | Used in the MPXV reservoir species model |
| BIO17 | Arotolu TE, et al. [1] | Monkeypox disease in southern Nigeria | Model: MaxEnt | Contributed 47% in the model, and precipitation of the driest quarter (15–25 mm) could most affect monkeypox ecological suitability. |
|  | Ellis CK, et.al. [6] | Monkeypox disease in Africa | Model: MaxEnt | Used in the monkeypox ecological niche model |
|  | Curaudeau M, et al. [2] | MPXV in Africa | Model: MaxEnt | Used in the MPXV ecological niche model |
|  | Fuller T, et.al. [3] | Monkeypox disease in the Congo Basin | Model: Logistic regression and Model: MaxEnt | Used in the monkeypox ecological niche model |
|  | Ellis CK, et.al. [6] | Monkeypox disease in Africa | Model: MaxEnt | Significant environmental variable |
|  | Thomassen HA, et.al. [8] | Monkeypox disease in Central Africa | Model: MaxEnt | Used in the MPXV reservoir species model |
| BIO18 | Arotolu TE, et al. [1] | Monkeypox disease in southern Nigeria | Model: MaxEnt | Used in the monkeypox ecological niche model |
|  | Curaudeau M, et al. [2] | MPXV in Africa | Model: MaxEnt | Used in the MPXV ecological niche model |
|  | Fuller T, et.al. [3] | Monkeypox disease in the Congo Basin | Model: Logistic regression and MaxEnt | Used in the monkeypox ecological niche model |
|  | Ellis CK, et.al. [6] | Monkeypox disease in Africa | Model: MaxEnt | Used in the monkeypox ecological niche model |
| BIO19 | Arotolu TE, et al. [1] | Monkeypox disease in southern Nigeria | Model: MaxEnt | Used in the monkeypox ecological niche model |
|  | Curaudeau M, et al. [2] | MPXV in Africa | Model: MaxEnt | Used in the MPXV ecological niche model |
|  | Fuller T, et.al. [3] | Monkeypox disease in the Congo Basin | Model: Logistic regression and MaxEnt | Used in the monkeypox ecological niche model |
|  | Ellis CK, et.al. [6] | Monkeypox disease in Africa | Model: MaxEnt | Used in the monkeypox ecological niche model |
| Leaf area index | Fuller T, et.al. [3] | Monkeypox disease in the Congo Basin | Model: Logistic regression and MaxEnt | Used in the monkeypox ecological niche model |
| Elevation | Arotolu TE, et al. [1] | Monkeypox disease in southern Nigeria | Model: MaxEnt | Contributed 26% in the model |
|  | Levine RS, et al. [4] | Monkeypox disease in Africa | Model: GARP | The variable has significant contributions to the model |
|  | Mandja BA, et.al. [5] | Monkeypox disease in the Democratic Republic of the Congo | Model: Bayesian hierarchical generalized linear mixed model | Used in the monkeypox ecological niche model |
|  | Thomassen HA, et.al. [8] | Monkeypox disease in Central Africa | Model: MaxEnt | Used in the MPXV reservoir species model |
| Latitude | Arotolu TE, et al. [1] | Monkeypox disease in southern Nigeria | Model: MaxEnt | The risk decreases as the latitude increases from the south toward the north |
|  | Cao Y, et.al. [9] | Temporal dynamic of monkeypox | Model: Pearson correlation | The growth rate (k) of the monkeypox epidemic showed significant relationships with the locations of the country latitude (r = –0.45, p = 0.038) |
| Mixed cropland and nature vegetation | Thomassen HA, et.al. [8] | Monkeypox disease in Central Africa | Model: MaxEnt | Land cover was used in the analysis of monkeypox infections in humans in Sankuru |
| Forest | Fuller T, et.al. [3] | Monkeypox disease in the Congo Basin | Model: Logistic regression and MaxEnt | The most important ecological variable for determining the ecological niche of the monkeypox virus |
|  | Mandja BA, et.al. [5] | Monkeypox disease in the Democratic Republic of the Congo | Model: Bayesian hierarchical generalized linear mixed model | The IRR of the variable is 1.034 (1.029-1.040) in the model |
|  | Thomassen HA, et.al. [8] | Monkeypox disease in Central Africa | Model: MaxEnt | Land cover was used in the analysis of monkeypox infections in humans in Sankuru |
| Shrubland | Thomassen HA, et.al. [8] | Monkeypox disease in Central Africa | Model: MaxEnt | Land cover was used in the analysis of monkeypox infections in humans in Sankuru |
| Grassland | Thomassen HA, et.al. [8] | Monkeypox disease in Central Africa | Model: MaxEnt | Land cover was used in the analysis of monkeypox infections in humans in Sankuru |
| Sparse vegetation land | Thomassen HA, et.al. [8] | Monkeypox disease in Central Africa | Model: MaxEnt | Land cover was used in the analysis of monkeypox infections in humans in Sankuru |
| Flooded vegetation | Thomassen HA, et.al. [8] | Monkeypox disease in Central Africa | Model: MaxEnt | Land cover was used in the analysis of monkeypox infections in humans in Sankuru |
| Bare areas | Thomassen HA, et.al. [8] | Monkeypox disease in Central Africa | Model: MaxEnt | Land cover was used in the analysis of monkeypox infections in humans in Sankuru |
| Water body | Thomassen HA, et.al. [8] | Monkeypox disease in Central Africa | Model: MaxEnt | Land cover was used in the analysis of monkeypox infections in humans in Sankuru |
| Cropland | Thomassen HA, et.al. [8] | Monkeypox disease in Central Africa | Model: MaxEnt | Land cover was used in the analysis of monkeypox infections in humans in Sankuru |
| Urban construction land | Thomassen HA, et.al. [8] | Monkeypox disease in Central Africa | Model: MaxEnt | Land cover was used in the analysis of monkeypox infections in humans in Sankuru |
| Population count | Arotolu TE, et al. [1] | Monkeypox disease in southern Nigeria | Model: MaxEnt | Contributed 17% in the model, and human population density (10 people/km^2^) can influence monkeypox transmission |
|  | Fuller T, et.al. [3] | Monkeypox disease in the Congo Basin | Model: Logistic regression and MaxEnt | Used in the monkeypox ecological niche model |
|  | Mandja BA, et.al. [5] | Monkeypox disease in the Democratic Republic of the Congo | Model: Bayesian hierarchical generalized linear mixed model | Used in the monkeypox ecological niche model |
| Global Downscaled GDP | Mandja BA, et.al. [5] | Monkeypox disease in the Democratic Republic of the Congo | Model: Bayesian hierarchical generalized linear mixed model | The IRR of the variable is 1.038 (1.031-1.047) in the model |
| Human footprint | Gallardo B, et.al. [10] | The global distribution of invaders | Model: MaxEnt | Factors related to the human footprint explained a substantial amount (23% on average) of species distributions |
|  | Skinner EB, et.al. [11] | The vector-borne diseases | Model: RF | Human footprint is an important predictor of local occurrence and that its nonlinear effects vary predictably with the transmission ecology of each vector-borne diseases |
| Rodentia richness | Usman S, et.al. [12] | Transmission dynamic of MPXV | Model: SEIR | Used in the monkeypox dynamic model |
| Primate richness | Usman S, et.al. [12] | Transmission dynamic of MPXV | Model: SEIR | Used in the monkeypox dynamic model |
| Mammalian richness | Olival KJ, et.al. [13] | Zoonotic spillover from mammals | Model: GAM | Used in the spillover model |
| *Funisciurus* spp. | Curaudeau M, et al. [2] | MPXV in Africa | Model: MaxEnt | *Funisciurus anerythrus* and *Funisciurus pyrropus* ranked first and third based on a niche overlap analysis between MPXV and mammal species, respectively |
|  | Fuller T, et.al. [3] | Monkeypox disease in the Congo Basin | Model: Logistic regression and MaxEnt | The second important ecological variable for determining the ecological niche of the MPXV |
|  | Thomassen HA, et.al. [8] | Monkeypox disease in Central Africa | Model: MaxEnt | *Funisciurus anerythrus* and *Funisciurus* congicus were the most important variables in the model |
|  | Khodakevich L, et.al. [14] | Laboratory testing of MPXV | Testing: isolation | MPXV was isolated from *Funisciurus anerythrus* |
|  | Tiee MS, et.al. [15] | Laboratory testing of MPXV | Testing: PCR | Historical distribution and prevalence of MPXV in *Funisciurus* spp. collected across Central Africa |
|  | Hutson CL, [16] | Laboratory testing of MPXV | Testing: PCR | Potential animal host of MPXV |
| *Graphiurus crassicaudatus* | Fuller T, et.al. [3] | Monkeypox disease in the Congo Basin | Model: Logistic regression and MaxEnt | Used in the monkeypox ecological niche model |
|  | Hutson CL, et.al. [16] | Laboratory testing of MPXV | Testing: PCR | Potential animal host of MPXV |
|  | Reynolds MG, et.al. [17] | Laboratory testing of Orthopoxvirus (OPXV) | Testing: PCR | Susceptible to natural OPXV infection |
| *Graphiurus lorraineus* | Fuller T, et.al. [3] | Monkeypox disease in the Congo Basin | Model: Logistic regression and MaxEnt | Used in the monkeypox ecological niche model |
|  | Curaudeau M, et al. [2] | MPXV in Africa | Model: MaxEnt | *Graphiurus lorraineus* ranked second based on a niche overlap analysis between MPXV and mammal species |
|  | Hutson CL, et.al. [16] | Laboratory testing of MPXV | Testing: PCR | Potential animal host of MPXV |
|  | Reynolds MG, et.al. [17] | Laboratory testing of OPXV | Testing: PCR | Susceptible to natural OPXV infection |
| *Cricetomys gambianus* | Fuller T, et.al. [3] | Monkeypox disease in the Congo Basin | Model: Logistic regression and MaxEnt | Used in the monkeypox ecological niche model |
|  | Thomassen HA, et.al. [8] | Monkeypox disease in Central Africa | Model: MaxEnt | Used in the MPXV ecological niche model |
|  | Hutson CL, et.al. [16] | Laboratory testing of MPXV | Testing: PCR | Potential animal host of MPXV |
|  | Reynolds MG, et.al. [17] | Laboratory testing of OPXV | Testing: PCR | Susceptible to natural OPXV infection |
|  | Falendysz EA, et.al. [18] | Laboratory testing of MPXV | Testing: Animal infection experiment | *Cricetomys gambianus* may play an important role in the transmission of the virus to humans |
|  | Hutson CL, et.al. [19] | Laboratory testing of MPXV | Testing: Animal infection experiment | *Cricetomys gambianus* may be involved in the maintenance of MPXV in wildlife mammalian populations |

**Supplementary reference**:

1. Arotolu TE, Afe AE, Wang H, Lv J, Shi K, Huang L, et al. Spatial modeling and ecological suitability of monkeypox disease in Southern Nigeria. *PLoS One*. 2022; 17(9): e0274325.

2. Curaudeau M, Besombes C, Nakouné E, Fontanet A, Gessain A, Hassanin A. Identifying the Most Probable Mammal Reservoir Hosts for Monkeypox Virus Based on Ecological Niche Comparisons. *Viruses*. 2023; 15(3): 727.

3. Fuller T, Thomassen HA, Mulembakani PM, Johnston SC, Lloyd-Smith JO, Kisalu NK, et al. Using remote sensing to map the risk of human monkeypox virus in the Congo Basin. *Ecohealth*. 2011;8(1):14-25.

4. Levine RS, Peterson AT, Yorita KL, Carroll D, Damon IK, Reynolds MG. Ecological niche and geographic distribution of human monkeypox in Africa. *PLoS One*. 2007; 2(1): e176.

5. Mandja BA, Handschumacher P, Bompangue D, Gonzalez JP, Muyembe JJ, Sauleau EA, et al. Environmental Drivers of Monkeypox Transmission in the Democratic Republic of the Congo. *Ecohealth*. 2022; 19(3): 354-364.

6. Ellis CK, Carroll DS, Lash RR, Peterson AT, Damon IK, Malekani J, et al. Ecology and geography of human monkeypox case occurrences across Africa. *J Wildl Di*s. 2012;48(2):335-347.

7. Lash RR, Carroll DS, Hughes CM, Nakazawa Y, Karem K, Damon IK, et al. Effects of georeferencing effort on mapping monkeypox case distributions and transmission risk. *Int J Health Geogr.* 2012; 11: 23.

8. Thomassen HA, Fuller T, Asefi-Najafabady S, Shiplacoff JA, Mulembakani PM, Blumberg S, et al. Pathogen-host associations and predicted range shifts of human monkeypox in response to climate change in central Africa. *PLoS One*. 2013; 8(7): e66071.

9. Cao Y, Li M, Haihambo N, Wang X, Zhao X, Wang B, et al. Temporal dynamic characteristics of human monkeypox epidemic in 2022 around the world under the COVID-19 pandemic background. *Front Public Health*. 2023; 11: 1120470.

10. Gallardo B, Zieritz A, Aldridge DC. The importance of the human footprint in shaping the global distribution of terrestrial, freshwater and marine invaders. *PLoS One*. 2015; 10(5): e0125801.

11. Skinner EB., Glidden CK., MacDonald AJ. Mordecai EA. Human footprint is associated with shifts in the assemblages of major vector-borne diseases. *Nat Sustain* 2023; 6, 652–661.

12. Usman S, Adamu I I. Modeling the transmission dynamics of the monkeypox virus infection with treatment and vaccination interventions. *J App Math Phys*, 2017; 5(12): 2335.

13. Olival KJ, Hosseini PR, Zambrana-Torrelio C, Ross N, Bogich TL, Daszak P. Host and viral traits predict zoonotic spillover from mammals. *Nature*; 2017; 546(7660): 646-650.

14. Khodakevich L, Jezek Z, Kinzanzka K. Isolation of monkeypox virus from wild squirrel infected in nature. *Lancet*. 1986; 1(8472): 98-99.

15. Tiee MS, Harrigan RJ, Thomassen HA, Smith TB. Ghosts of infections past: using archival samples to understand a century of monkeypox virus prevalence among host communities across space and time. *R Soc Open Sci*. 2018; 5(1): 171089.

16. Hutson CL, Lee KN, Abel J, Carroll DS, Montgomery JM, Olson VA, et al. Monkeypox zoonotic associations: insights from laboratory evaluation of animals associated with the multi-state US outbreak. *Am J Trop Med Hyg*. 2007; 76(4): 757-768.

17. Reynolds MG, Carroll DS, Olson VA, Hughes C, Galley J, Likos A, et al. A silent enzootic of an orthopoxvirus in Ghana, West Africa: evidence for multi-species involvement in the absence of widespread human disease. *Am J Trop Med Hyg*. 2010; 82(4): 746-754.

18. Falendysz EA, Lopera JG, Lorenzsonn F, Salzer JS, Hutson CL, Doty J, et al. Further Assessment of Monkeypox Virus Infection in Gambian Pouched Rats (*Cricetomys gambianus*) Using In Vivo Bioluminescent Imaging. *PLoS Negl Trop Dis*. 2015; 9(10): e0004130.

19. Hutson CL, Nakazawa YJ, Self J, Olson VA, Regnery RL, Braden Z, et al. Laboratory Investigations of African Pouched Rats (*Cricetomys gambianus*) as a Potential Reservoir Host Species for Monkeypox Virus. *PLoS Negl Trop Dis*. 2015; 9(10): e0004013.

# Appendix Table S12: Number and resource of occurrence data for animals extracted from Global Biodiversity Information Facility (GBIF)

| **Animal** | **Number of original occurrences** | **Number of occurrences after removal of deduplication** | **Citation** | **DOI** |
| --- | --- | --- | --- | --- |
| *Cricetomys gambianus* | 955 | 547^*^ | GBIF Occurrence Download. June 7, 2022. https://doi.org/10.15468/dl.rz39au (accessed June 7, 2022) | 10.15468/dl.rz39au |
| *Funisciurus* spp. | 3174 | 715 | GBIF Occurrence Download. June 7, 2022. https://doi.org/10.15468/dl.gtrp3e (accessed June 7, 2022) | 10.15468/dl.gtrp3e |
| *Graphiurus crassicaudatus* | 90 | 47 | GBIF Occurrence Download. June 7, 2022. https://doi.org/10.15468/dl.qrawf2 (accessed June 7, 2022) | 10.15468/dl.qrawf2 |
| *Graphiurus lorraineus* | 380 | 88 | GBIF Occurrence Download. June 7, 2022. https://doi.org/10.15468/dl.ag82kr (accessed June 7, 2022) | 10.15468/dl.ag82kr |

*: After modifying the spatial range of *Cricetomys gambianus* by using the data from ICUN, only 375 occurrences were used in this study.

# Appendix Table S13: Source of data to describe the yearly case number in infected regions

| Year | Region | Case number | Reference |
| --- | --- | --- | --- |
| 2003 | America | 87 | 76 |
| 2021 | America | 2 | 169 |
| 2022 | America | 4052 | Global health |
| 2018 | Asia | 1 | 10 |
| 2019 | Asia | 1 | 37 |
| 2022 | Asia | 134 | Global health |
| 2005 | Eastern Africa | 19 | 83 |
| 2018 | Europe | 3 | 33 |
| 2019 | Europe | 1 | 33 |
| 2021 | Europe | 3 | 33 |
| 2022 | Europe | 11982 | Global health |
| 1970 | Central Africa | 1 | 111 |
| 1972 | Central Africa | 5 | 88, 98 |
| 1973 | Central Africa | 3 | 88, 98 |
| 1974 | Central Africa | 1 | 88, 98 |
| 1975 | Central Africa | 3 | 88, 98 |
| 1976 | Central Africa | 5 | 88, 98 |
| 1977 | Central Africa | 6 | 88, 98 |
| 1978 | Central Africa | 12 | 88, 98 |
| 1979 | Central Africa | 8 | 3, 98, 111 |
| 1980 | Central Africa | 3 | 98 |
| 1981 | Central Africa | 6 | 98 |
| 1982 | Central Africa | 37 | 98 |
| 1983 | Central Africa | 54 | 98 |
| 1984 | Central Africa | 9 | 98, 111 |
| 1987 | Central Africa | 5 | 3, 111 |
| 1989 | Central Africa | 1 | 3, 111 |
| 1996 | Central Africa | 180 | 61, 65 |
| 1997 | Central Africa | 369 | 61, 65 |
| 2001 | Central Africa | 392 | 97, 111 |
| 2002 | Central Africa | 881 | 97 |
| 2003 | Central Africa | 766 | 3, 97 |
| 2004 | Central Africa | 1024 | 97 |
| 2005 | Central Africa | 1708 | 97 |
| 2006 | Central Africa | 783 | 97 |
| 2007 | Central Africa | 970 | 97 |
| 2008 | Central Africa | 1599 | 97 |
| 2009 | Central Africa | 1921 | 3, 97 |
| 2010 | Central Africa | 2324 | 97, 111 |
| 2011 | Central Africa | 2208 | 97 |
| 2012 | Central Africa | 2629 | 97 |
| 2013 | Central Africa | 2460 | 97 |
| 2015 | Central Africa | 7 | 111 |
| 2016 | Central Africa | 17 | 112 |
| 2017 | Central Africa | 96 | 9, 111 |
| 2018 | Central Africa | 2869 | 58, 111, 163 |
| 2019 | Central Africa | 3835 | 111, 163, 167 |
| 2020 | Central Africa | 4594 | 163 |
| 2022 | Central Africa | 23 | 163, Global health |
| 2022 | Northern Africa | 1 | Global health |
| 2022 | Oceania | 43 | Global health |
| 2022 | Southern Africa | 3 | Global health |
| 1970 | Western Africa | 5 | 89,111 |
| 1971 | Western Africa | 3 | 89, 111 |
| 1978 | Western Africa | 1 | 89, 111 |
| 1981 | Western Africa | 1 | 3, 111 |
| 2014 | Western Africa | 1 | 111 |
| 2017 | Western Africa | 288 | 3, 111, 140 |
| 2018 | Western Africa | 163 | 137 |
| 2019 | Western Africa | 160 | 131 |
| 2020 | Western Africa | 23 | 125 |
| 2021 | Western Africa | 125 | 117 |
| 2022 | Western Africa | 138 | 163, Global health |

# Appendix Table S14: Source of data to describe the monthly case number in infected regions

| Month | **Region** | **Cases** | **Reference ID** |
| --- | --- | --- | --- |
| 1 | Western Africa | 10 | 62, 79, 82, 89, 91, 112, 113, 114, 115, 116, 117, 118, 119, 120, 121, 122, 123, 124, 125, 126, 127, 128 |
| 2 | Western Africa | 18 | 112, 113, 114, 115, 116, 117, 118, 119, 120, 121, 122, 123, 124, 125, 126, 127, 128 |
| 3 | Western Africa | 13 | 24, 79, 112, 113, 114, 115, 116, 117, 118, 119, 120, 121, 122, 123, 124, 125, 126, 127, 128 |
| 4 | Western Africa | 22 | 51, 89, 112, 113, 114, 115, 116, 117, 118, 119, 120, 121, 122, 123, 124, 125, 126, 127, 128, Global health |
| 5 | Western Africa | 19 | 51, 112, 113, 114, 115, 116, 117, 118, 119, 120, 121, 122, 123, 124, 125, 126, 127, 128, Global health |
| 6 | Western Africa | 29 | 51, 112, 113, 114, 115, 116, 117, 118, 119, 120, 121, 122, 123, 124, 125, 126, 127, 128 |
| 7 | Western Africa | 12 | 51, 112, 113, 114, 115, 116, 117, 118, 119, 120, 121, 122, 123, 124, 125, 126, 127, 128 |
| 8 | Western Africa | 12 | 51, 112, 113, 114, 115, 116, 117, 118, 119, 120, 121, 122, 123, 124, 125, 126, 127, 128 |
| 9 | Western Africa | 11 | 3, 15, 19, 51, 82, 89, 112, 113, 114, 115, 116, 117, 118, 119, 120, 121, 122, 123, 124, 125, 126, 127, 128 |
| 10 | Western Africa | 48 | 3, 15, 51, 82, 89, 112, 113, 114, 115, 116, 117, 118, 119, 120, 121, 122, 123, 124, 125, 126, 127, 128 |
| 11 | Western Africa | 34 | 15, 51, 89, 112, 113, 114, 115, 116, 117, 118, 119, 120, 121, 122, 123, 124, 125, 126, 127, 128 |
| 12 | Western Africa | 45 | 15, 82, 112, 113, 114, 115, 116, 117, 118, 119, 120, 121, 122, 123, 124, 125, 126, 127, 128 |
| 6 | Eastern Africa | 10 | Global health |
| 9 | Eastern Africa | 2 | 83 |
| 10 | Eastern Africa | 11 | 83 |
| 11 | Eastern Africa | 4 | 83 |
| 12 | Eastern Africa | 2 | 83 |
| 5 | Northern Africa | 1 | Global health |
| 6 | Northern Africa | 1 | Global health |
| 1 | Central Africa | 7 | 2, 5, 9, 28, 41, 57, 85, 89 |
| 2 | Central Africa | 2 | 9, 11, 28, 53, 58 |
| 3 | Central Africa | 38 | 9, 28, 53, 58 |
| 5 | Central Africa | 380 | 9, 13, 14, 53, 58, 60, Global health |
| 6 | Central Africa | 383 | 9, 38, 52, 53, 60 |
| 7 | Central Africa | 1 | Global health |
| 9 | Central Africa | 3 | 9, 14, 46, 53, 91 |
| 10 | Central Africa | 3 | 9, 46, 53 |
| 6 | Southern Africa | 2 | Global health |
| 7 | Southern Africa | 1 | Global health |
| 12 | Central Africa | 7 | 1, 2, 9, 11, 89, 64 |
| 5 | Europe | 564 | 33, 67, 108, 109, 110, 111, Global health |
| 6 | Europe | 4538 | Global health |
| 7 | Europe | 6886 | Global health |
| 9 | Europe | 3 | 6, 23, 33 |
| 1 | America | 2 | 169 |
| 5 | America | 92 | 7, 34, 36, 44, 48, 68, 69, 76, 77, 79, 82, Global health |
| 6 | America | 838 | 7, 12, 44, 48, 68, 69, 76, 79, 82 |
| 7 | America | 3221 | 22, 79, 82, Global health |
| 4 | Asia | 1 | 37, Global health |
| 5 | Asia | 10 | Global health |
| 6 | Asia | 55 | Global health |
| 7 | Asia | 73 | Global health |
| 10 | Asia | 1 | 10, 23, 62 |
| 5 | Oceania | 2 | Global health |
| 6 | Oceania | 10 | Global health |
| 7 | Oceania | 31 | Global health |

# Appendix Table S15: Epidemiological features of global human monkeypox patients recorded in 2022

| Features | **All** | **Central Africa** | **Western Africa** | **Other Regions in Africa^#^** | **Europe** | **America** | **Other areas** |
| --- | --- | --- | --- | --- | --- | --- | --- |
| Total | 16754 | 24 | 476 | 15 | 11985 | 4073 | 181 |
| Type |  |  |  |  |  |  |  |
| Confirmed | 16376 (97.74%) | 24 (100.00%) | 138 (28.99%) | 4 (26.67%) | 11982 (99.97%) | 4051 (99.46%) | 177 (97.79%) |
| Suspect or probable | 378 (2.26%) | 0 (0.00%) | 338 (71.01%) | 11 (73.33%) | 3 (0.03%) | 22 (0.54%) | 4 (2.21%) |
| Gender† |  |  |  |  |  |  |  |
| Male | 1609 (96.46%) | 0 (0.00%) | 80 (68.38%) | 3 (60.00%) | 1034 (99.33%) | 462 (97.47%) | 30 (96.77%) |
| Female | 59 (3.54%) | 0 (0.00%) | 37 (31.62%) | 2 (40.00%) | 7 (0.67%) | 12 (2.53%) | 1 (3.23%) |
| Age† |  |  |  |  |  |  |  |
| 0-14 | 19 (3.49%) | 0 (0.00%) | 15 (12.82%) | 2 (33.33%) | 1 (0.57%) | 0 (0.00%) | 1 (4.17%) |
| 15-29 | 71 (13.03%) | 0 (0.00%) | 48 (41.03%) | 1 (16.67%) | 7 (4.02%) | 14 (6.25%) | 1 (4.17%) |
| 30-59 | 452 (82.94%) | 0 (0.00%) | 54 (46.15%) | 3 (50.00%) | 163 (93.68%) | 210 (93.75%) | 22 (91.67%) |
| ≥60 | 3 (0.55%) | 0 (0.00%) | 0 (0.00%) | 0 (0.00%) | 3 (1.72%) | 0 (0.00%) | 0 (0.00%) |
| Testing Method*† |  |  |  |  |  |  |  |
| PCR | 92 (95.83%) | 0 (0.00%) | 0 (0.00%) | 0 (0.00%) | 85 (95.51%) | 5 (100.00%) | 2 (100.00%) |
| Molecular sequencing | 2 (2.08%) | 0 (0.00%) | 0 (0.00%) | 0 (0.00%) | 2 (2.25%) | 0 (0.00%) | 0 (0.00%) |
| Virus isolation | 0 (0.00%) | 0 (0.00%) | 0 (0.00%) | 0 (0.00%) | 0 (0.00%) | 0 (0.00%) | 0 (0.00%) |
| Electron microscope | 2 (2.08%) | 0 (0.00%) | 0 (0.00%) | 0 (0.00%) | 2 (2.25%) | 0 (0.00%) | 0 (0.00%) |
| Unknown |  |  |  |  |  |  |  |
| Inpatient† |  |  |  |  |  |  |  |
| Yes | 88 (43.14%) | 0 (0.00%) | 0 (0.00%) | 0 (0.00%) | 63 (42.57%) | 12 (30.00%) | 13 (81.25%) |
| No | 116 (56.86%) | 0 (0.00%) | 0 (0.00%) | 0 (0.00%) | 85 (57.43%) | 28 (70.00%) | 3 (18.75%) |
| Transmission model† |  |  |  |  |  |  |  |
| Zoonotic infection | 0 (0.00%) | 0 (0.00%) | 0 (0.00%) | 0 (0.00%) | 0 (0.00%) | 0 (0.00%) | 0 (0.00%) |
| Community transmission | 142 (28.63%) | 0 (0.00%) | 0 (0.00%) | 0 (0.00%) | 135 (36.99%) | 7 (6.67%) | 0 (0.00%) |
| Travel-related infection | 186 (37.50%) | 0 (0.00%) | 2 (100.00%) | 4 (100.00%) | 77 (21.1%) | 83 (79.05%) | 20 (100%) |
| Probable MSM-related infection | 163 (32.86%) | 0 (0.00%) | 0 (0.00%) | 0 (0.00%) | 148 (40.55%) | 15 (14.29%) | 0 (0.00%) |
| Household transmission | 2 (0.40%) | 0 (0.00%) | 0 (0.00%) | 0 (0.00%) | 2 (0.55%) | 0 (0.00%) | 0 (0.00%) |
| Infections via animal trade | 1 (0.20%) | 0 (0.00%) | 0 (0.00%) | 0 (0.00%) | 1 (0.27%) | 0 (0.00%) | 0 (0.00%) |
| Nosocomial infection | 2 (0.40%) | 0 (0.00%) | 0 (0.00%) | 0 (0.00%) | 2 (0.55%) | 0 (0.00%) | 0 (0.00%) |
| Outcome† |  |  |  |  |  |  |  |
| Recover | 482 (98.97%) | 21 (95.45%) | 454 (99.00%) | 0 (0.00%) | 6 (1.00%) | 0 (0.00%) | 1 (100%) |
| Death | 5 (1.03%) | 2 (4.55%) | 3 (1.00%) | 0 (0.00%) | 0 (0.00%) | 0 (0.00%) | 0 (0.00%) |

^#^Other Regions in Africa: Eastern Africa, Southern Africa and Northern Africa. † Data are included with specific information reported. *Only confirmed cases are included.

# Appendix Table S16: Case numbers of MPXV infections in humans by country and year

| **ID** | **Year** | **Continent** | **Country** | **Case number** | **Reference** |
| --- | --- | --- | --- | --- | --- |
| 1 | 1970 | Central Africa | Democratic Republic of the Congo | 1 | 111 |
| 2 | 1970 | Western Africa | Liberia | 4 | 89 |
| 3 | 1970 | Western Africa | Sierra Leone | 1 | 111 |
| 4 | 1971 | Western Africa | Côte d’Ivoire | 1 | 89, 111 |
| 5 | 1971 | Western Africa | Nigeria | 2 | 89, 111 |
| 6 | 1972 | Central Africa | Democratic Republic of the Congo | 5 | 88, 98 |
| 7 | 1973 | Central Africa | Democratic Republic of the Congo | 3 | 88, 98 |
| 8 | 1974 | Central Africa | Democratic Republic of the Congo | 1 | 88, 98 |
| 9 | 1975 | Central Africa | Democratic Republic of the Congo | 3 | 88, 98 |
| 10 | 1976 | Central Africa | Democratic Republic of the Congo | 5 | 88, 98 |
| 11 | 1977 | Central Africa | Democratic Republic of the Congo | 6 | 88, 98 |
| 12 | 1978 | Central Africa | Democratic Republic of the Congo | 12 | 88, 98 |
| 13 | 1978 | Western Africa | Nigeria | 1 | 89, 111 |
| 14 | 1979 | Central Africa | Cameroon | 1 | 3, 111 |
| 15 | 1979 | Central Africa | Democratic Republic of the Congo | 7 | 98 |
| 16 | 1980 | Central Africa | Democratic Republic of the Congo | 3 | 98 |
| 17 | 1981 | Western Africa | Côte d’Ivoire | 1 | 3, 111 |
| 18 | 1981 | Central Africa | Democratic Republic of the Congo | 6 | 98 |
| 19 | 1982 | Central Africa | Democratic Republic of the Congo | 37 | 98 |
| 20 | 1983 | Central Africa | Democratic Republic of the Congo | 54 | 98 |
| 21 | 1984 | Central Africa | Central African Republic | 6 | 111 |
| 22 | 1984 | Central Africa | Democratic Republic of the Congo | 3 | 98 |
| 23 | 1987 | Central Africa | Gabon | 5 | 3, 111 |
| 24 | 1989 | Central Africa | Cameroon | 1 | 3, 111 |
| 25 | 1996 | Central Africa | Democratic Republic of the Congo | 180 | 61, 65 |
| 26 | 1997 | Central Africa | Democratic Republic of the Congo | 369 | 61, 65 |
| 27 | 2001 | Central Africa | Central African Republic | 4 | 111 |
| 28 | 2001 | Central Africa | Democratic Republic of the Congo | 388 | 97 |
| 29 | 2002 | Central Africa | Democratic Republic of the Congo | 881 | 97 |
| 30 | 2003 | Central Africa | Democratic Republic of the Congo | 755 | 97 |
| 31 | 2003 | Central Africa | Republic of Congo | 11 | 3 |
| 32 | 2003 | America | USA | 87 | 76 |
| 33 | 2004 | Central Africa | Democratic Republic of the Congo | 1024 | 97 |
| 34 | 2005 | Central Africa | Democratic Republic of the Congo | 1708 | 97 |
| 35 | 2005 | Eastern Africa | South Sudan | 19 | 83 |
| 36 | 2006 | Central Africa | Democratic Republic of the Congo | 783 | 97 |
| 37 | 2007 | Central Africa | Democratic Republic of the Congo | 970 | 97 |
| 38 | 2008 | Central Africa | Democratic Republic of the Congo | 1599 | 97 |
| 39 | 2009 | Central Africa | Democratic Republic of the Congo | 1919 | 97 |
| 40 | 2009 | Central Africa | Republic of Congo | 2 | 3 |
| 41 | 2010 | Central Africa | Central African Republic | 2 | 111 |
| 42 | 2010 | Central Africa | Democratic Republic of the Congo | 2322 | 97 |
| 43 | 2011 | Central Africa | Democratic Republic of the Congo | 2208 | 97 |
| 44 | 2012 | Central Africa | Democratic Republic of the Congo | 2629 | 97 |
| 45 | 2013 | Central Africa | Democratic Republic of the Congo | 2460 | 97 |
| 46 | 2014 | Western Africa | Sierra Leone | 1 | 111 |
| 47 | 2015 | Central Africa | Central African Republic | 7 | 111 |
| 48 | 2016 | Central Africa | Central African Republic | 16 | 111 |
| 49 | 2016 | Central Africa | Democratic Republic of the Congo | 1 | 1 |
| 50 | 2017 | Central Africa | Central African Republic | 8 | 111 |
| 51 | 2017 | Western Africa | Liberia | 2 | 3 |
| 52 | 2017 | Western Africa | Nigeria | 285 | 140 |
| 53 | 2017 | Central Africa | Republic of Congo | 88 | 9 |
| 54 | 2017 | Western Africa | Sierra Leone | 1 | 111 |
| 55 | 2018 | Central Africa | Cameroon | 7 | 58 |
| 56 | 2018 | Central Africa | Central African Republic | 12 | 111 |
| 57 | 2018 | Central Africa | Democratic Republic of the Congo | 2850 | 163 |
| 58 | 2018 | Asia | Israel | 1 | 10 |
| 59 | 2018 | Western Africa | Nigeria | 163 | 137 |
| 60 | 2018 | Europe | UK | 3 | 33 |
| 61 | 2019 | Central Africa | Cameroon | 3 | 111, 167 |
| 62 | 2019 | Central Africa | Central African Republic | 38 | 111 |
| 63 | 2019 | Central Africa | Democratic Republic of the Congo | 3794 | 163 |
| 64 | 2019 | Western Africa | Nigeria | 160 | 131 |
| 65 | 2019 | Asia | Singapore | 1 | 37 |
| 66 | 2019 | Europe | UK | 1 | 33 |
| 67 | 2020 | Central Africa | Democratic Republic of the Congo | 4594 | 163 |
| 68 | 2020 | Western Africa | Nigeria | 23 | 125 |
| 69 | 2021 | Western Africa | Nigeria | 125 | 117 |
| 70 | 2021 | Europe | UK | 3 | 33 |
| 71 | 2021 | America | USA | 2 | 169 |
| 72 | 2022 | America | Argentina | 13 | Global health |
| 73 | 2022 | Oceania | Australia | 41 | Global health |
| 74 | 2022 | Europe | Austria | 99 | Global health |
| 75 | 2022 | America | Barbados | 1 | Global health |
| 76 | 2022 | Europe | Belgium | 311 | Global health |
| 77 | 2022 | Western Africa | Benin | 3 | Global health |
| 78 | 2022 | Europe | Bosnia and Herzegovina | 1 | Global health |
| 79 | 2022 | America | Brazil | 607 | Global health |
| 80 | 2022 | Europe | Bulgaria | 3 | Global health |
| 81 | 2022 | Central Africa | Cameroon | 3 | 163 |
| 82 | 2022 | America | Canada | 604 | Global health |
| 83 | 2022 | Central Africa | Central African Republic | 8 | Global health |
| 84 | 2022 | America | Chile | 26 | Global health |
| 85 | 2022 | Asia | China | 2 | Global health |
| 86 | 2022 | America | Colombia | 10 | Global health |
| 87 | 2022 | America | Costa Rica | 1 | Global health |
| 88 | 2022 | Europe | Croatia | 7 | Global health |
| 89 | 2022 | Europe | Czechia | 14 | Global health |
| 90 | 2022 | Central Africa | Democratic Republic of the Congo | 10 | 163 |
| 91 | 2022 | Europe | Denmark | 48 | Global health |
| 92 | 2022 | America | Dominican Republic | 3 | Global health |
| 93 | 2022 | America | Ecuador | 2 | Global health |
| 94 | 2022 | Europe | Estonia | 4 | Global health |
| 95 | 2022 | Europe | Finland | 13 | Global health |
| 96 | 2022 | Europe | France | 1562 | Global health |
| 97 | 2022 | Asia | Georgia | 1 | Global health |
| 98 | 2022 | Europe | Germany | 2268 | Global health |
| 99 | 2022 | Western Africa | Ghana | 18 | Global health |
| 100 | 2022 | Europe | Gibraltar | 5 | Global health |
| 101 | 2022 | Europe | Greece | 19 | Global health |
| 102 | 2022 | America | Haiti | 1 | Global health |
| 103 | 2022 | Europe | Hungary | 32 | Global health |
| 104 | 2022 | Europe | Iceland | 6 | Global health |
| 105 | 2022 | Asia | India | 2 | Global health |
| 106 | 2022 | Europe | Ireland | 69 | Global health |
| 107 | 2022 | Asia | Israel | 105 | Global health |
| 108 | 2022 | Europe | Italy | 407 | Global health |
| 109 | 2022 | America | Jamaica | 1 | Global health |
| 110 | 2022 | Europe | Latvia | 3 | Global health |
| 111 | 2022 | Asia | Lebanon | 1 | Global health |
| 112 | 2022 | Europe | Luxembourg | 14 | Global health |
| 113 | 2022 | Europe | Malta | 17 | Global health |
| 114 | 2022 | America | Martinique | 1 | Global health |
| 115 | 2022 | America | Mexico | 48 | Global health |
| 116 | 2022 | Northern Africa | Morocco | 1 | Global health |
| 117 | 2022 | Europe | Netherlands | 712 | Global health |
| 118 | 2022 | Oceania | New Zealand | 2 | Global health |
| 119 | 2022 | Western Africa | Nigeria | 117 | 163 |
| 120 | 2022 | Europe | Norway | 46 | Global health |
| 121 | 2022 | America | Panama | 1 | Global health |
| 122 | 2022 | America | Peru | 143 | Global health |
| 123 | 2022 | Europe | Poland | 40 | Global health |
| 124 | 2022 | Europe | Portugal | 588 | Global health |
| 125 | 2022 | America | Puerto Rico | 8 | Global health |
| 126 | 2022 | Asia | Qatar | 1 | Global health |
| 127 | 2022 | Central Africa | Republic of Congo | 2 | 163 |
| 128 | 2022 | Europe | Romania | 19 | Global health |
| 129 | 2022 | Europe | Russia | 1 | Global health |
| 130 | 2022 | Asia | Saudi Arabia | 1 | Global health |
| 131 | 2022 | Europe | Serbia | 5 | Global health |
| 132 | 2022 | Asia | Singapore | 6 | Global health |
| 133 | 2022 | Europe | Slovakia | 3 | Global health |
| 134 | 2022 | Europe | Slovenia | 27 | Global health |
| 135 | 2022 | Southern Africa | South Africa | 3 | Global health |
| 136 | 2022 | Asia | South Korea | 1 | Global health |
| 137 | 2022 | Europe | Spain | 3125 | Global health |
| 138 | 2022 | Europe | Sweden | 77 | Global health |
| 139 | 2022 | Europe | Switzerland | 229 | Global health |
| 140 | 2022 | Asia | Turkey | 1 | Global health |
| 141 | 2022 | Europe | UK | 2208 | Global health |
| 142 | 2022 | Asia | United Arab Emirates | 13 | Global health |
| 143 | 2022 | America | USA | 2581 | Global health |
| 144 | 2022 | America | Venezuela | 1 | Global health |

Only confirmed cases were included in 2022

# Appendix Table S17: Cases of MPXV infections in animals by country and year

| **Site** | **Country** | **Date range** | **Location** | **Diagnosis** | **Animals** | **Reference ID** |
| --- | --- | --- | --- | --- | --- | --- |
| 1 | Denmark | 1958 |  | Isolation | *Macaca fascicularis* | 103 |
| 2 | Democratic Republic of Congo | 1979 | Yambuku | Serology | *Funisciurus anerythrus* | 99 |
| 3 | Democratic Republic of Congo | 1985 | Yambuku | Isolation | *Funisciurus anerythrus* | 99 |
| 4 | USA | 2003 |  | RT-PCR | Prairie dogs | 95 |
| 5 | USA | 2003 | Texas | PCR & Isolation | *Funiscuirus* spp. | 69 |
| 6 | USA | 2003 | Texas | PCR & Isolation | *Cricetomys* spp. | 69 |
| 7 | USA | 2003 | Texas | PCR & Isolation | *Graphiurus* spp. | 69 |
| 8 | Côte d'Ivoire | 2012 | Taï National Park | Isolation | Monkey | 100 |
| 9 | Côte d'Ivoire | 1969-1972 |  | Serology | *Cercopithecus aethiops* | 35 |
| 10 | Côte d'Ivoire | 1969-1972 |  | Serology | *Cercopithecus petaurista* | 35 |
| 11 | Democratic Republic of Congo | 1985-1987 | Bumba | Serology | *Cercopithecus ascanius* | 107 |
| 12 | Democratic Republic of Congo | 1985-1987 | Ikela | Serology | *Cercopithecus ascanius* | 107 |
| 13 | Democratic Republic of Congo | 1985-1987 | Bumba | Serology | *Funiscuirus* spp. | 107 |
| 14 | Democratic Republic of Congo | 1985-1987 | Ikela | Serology | *Funiscuirus* spp. | 107 |
| 15 | Democratic Republic of Congo | 1985-1987 | Bandundu | Serology | *Funiscuirus* spp. | 107 |
| 16 | Democratic Republic of Congo | 1985-1987 | Bas-Zaire | Serology | *Heliosciurus* spp. | 107 |
| 17 | Democratic Republic of Congo | 1985-1987 | Bumba | Serology | *Heliosciurus* spp. | 107 |
| 18 | Democratic Republic of Congo | 1985-1987 | Ikela | Serology | *Heliosciurus* spp. | 107 |
| 19 | Democratic Republic of Congo | 1985-1987 | Bas-Zaire | Serology | *Funisciurus* spp. | 107 |
| 22 | Democratic Republic of Congo | 1997/02/23-1997/02/27 | Katako-Kombe Health Zone | Serology | Domestic pig | 96 |
| 23 | Democratic Republic of Congo | 1997/02/23-1997/02/27 | Katako-Kombe Health Zone | Serology | *Cricetomys emini* | 96 |
| 24 | Democratic Republic of Congo | 1997/02/23-1997/02/27 | Katako-Kombe Health Zone | Serology | *Petrodromus tetradactylus* | 96 |
| 25 | Democratic Republic of Congo | 1997/02/23-1997/02/27 | Katako-Kombe Health Zone | Serology | *Funisciurus anerythrus* | 96 |
| 26 | Democratic Republic of Congo | 1997/02/23-1997/02/27 | Katako-Kombe Health Zone | Serology | *Funisciurus congicus* | 96 |
| 27 | Democratic Republic of Congo | 1997/02/23-1997/02/27 | Katako-Kombe Health Zone | Serology | *Heliosciurus rufobrachium* | 96 |
| 28 | Côte d'Ivoire | 2016/12/1-2017/4/29 | South of Taï National Park | PCR | *Pan troglodytes* | 101 |
| 29 | Côte d'Ivoire | 2017.1-2018.5 | Taï National Park | PCR | Chimpanzee | 101 |
| 30 | Côte d'Ivoire | 2017.1-2018.5 | Taï National Park | PCR | Fly | 101 |
| 31 | Côte d'Ivoire | 2017/1/28-2017/5/19 | North of Taï National Park | PCR | *Pan troglodytes* | 101 |
| 32 | Côte d'Ivoire | 2017/4/1-2017/8/25 | East of Taï National Park | PCR | *Pan troglodytes* | 101 |

# Appendix Table S18: Locations of detection of MPXV in animals with transmission to humans

| **Site No.** | **Date range** | **Country** | **Location** | **Diagnosis** | **Animal** | **Reference ID** |
| --- | --- | --- | --- | --- | --- | --- |
| 1 | 2015 | Central African Republic | Bakouma, Madigui village | PCR | *Thryonomis* spp. | 2 |
| 2 | 2017 | Congo | Likouala Department | Serology | Wild animals | 5 |
| 3 | 2015-2016 | Central African Republic | Bangassou District | PCR | Rodent | 11 |
| 4 | 1983 | Democratic Republic of the Congo | the West Kasai region,Iyeke | Serology | *Colobus* *pennanti oustaleti* | 13 |
| 5 | 2001 | Democratic Republic of the Congo | Liyefe | PCR | Monkey | 20 |
| 6 | 2017 | Sierra Leone | Pujehun, Galliness Perri, Kpaku village | PCR | Squirrel | 24 |
| 7 | 2016 | Central African Republic |  | PCR, Isolation | Squirrel | 27 |
| 8 | 2017 | Congo | Likouala Department, Dongou | PCR, Serology | Wild animals | 28 |
| 9 | 2017 | Congo | Likouala Department, Impfondo | PCR, Serology | Rodent | 28 |
| 10 | 2010 | Central African Republic |  | PCR | Rodent | 38 |
| 11 | 2011-2015 | Democratic Republic of the Congo | Tshuapa | PCR | Non-human primates, Rats, Squirrels | 42 |
| 12 | 2018 | Central African Republic | Lobaye | PCR | *Civettictis civetta*, *Cricetomys emini*, *Funesciurus anerythrus* | 46 |
| 13 | 2010 | Central African Republic |  | PCR | Rodent | 52 |
| 14 | 1996 | Democratic Republic of the Congo | Kasai Oriental | PCR | Squirrel | 61 |
| 15 | 2011 | Democratic Republic of the Congo | North Kivu | PCR | Monkey | 64 |
| 16 | 1981-1986 | Democratic Republic of the Congo |  | Serology | wild animals | 90 |
| 17 | 2017/9-2018 | Nigeria |  | Unknown | Rodents | 93 |
| 18 | 2013/7/1-2013/12/8 | Democratic Republic of the Congo | Tshuapa, Bokungu Health Zone | PCR | Monkey, Rat, Squirrels | 94 |
| 19 | 1996/02-1997/02 | Democratic Republic of the Congo | Katako-Kombe, Katako-Kombe Health Zone, located around Akungula | PCR, Isolation | Squirrel, Monkey, Rat, Porcupines, Gazelles | 96 |
| 20 | 1981-1986 | Democratic Republic of the Congo |  | Serology | Wild animals | 74 |
| 21 | 2014 | Sierra Leone | Bo, Kpetema town | PCR | Rodent | 79 |
| 22 | 1982 | Democratic Republic of the Congo | Kivu, Puma, Kibwe | Isolation | Chimpanzee | 85 |
| 23 | 2017 | Nigeria |  | PCR, Serology | Monkey | 19 |
| 24 | 2017-2018 | Nigeria |  | PCR, Serology | Monkeys, Rodents, Other wild animal, Domestic animals | 23 |

# Appendix Table S19: Locations with MPXV infection in humans and animals used to build BRT models

| **ID** | **Longitude** | **Latitude** | **Point/Polygon** | **Country** | **Positive Hosts** | **Reference ID** |
| --- | --- | --- | --- | --- | --- | --- |
| 1 | -8.37 | 6.07 | Point | Liberia | Human | 171 |
| 2 | -1.27 | 8.16 | Point | Ghana | Human | 171 |
| 3 | -0.20 | 5.91 | Point | Ghana | Human | 171 |
| 4 | 18.08 | 1.67 | Point | Republic of Congo | Human | 171 |
| 5 | 21.93 | 2.39 | Point | Democratic Republic of the Congo | Human | 171 |
| 6 | 22.24 | 2.91 | Point | Democratic Republic of the Congo | Human | 171 |
| 7 | 24.23 | -3.48 | Point | Democratic Republic of the Congo | Human | 171 |
| 8 | -1.24 | 8.18 | Point | Ghana | Human | 171 |
| 9 | -8.19 | 6.13 | Point | Liberia | Human | 171 |
| 10 | -6.45 | 6.81 | Point | Côte d'Ivoire | Human | 171 |
| 11 | -3.43 | 6.60 | Point | Côte d'Ivoire | Human | 171 |
| 12 | 4.63 | 7.19 | Point | Nigeria | Human | 171 |
| 13 | 7.38 | 5.05 | Point | Nigeria | Human | 171 |
| 14 | 11.52 | 3.68 | Point | Cameroon | Human | 171 |
| 15 | 11.73 | 3.61 | Point | Cameroon | Human | 171 |
| 16 | 16.18 | 2.69 | Point | Central African Republic | Human | 171 |
| 17 | 18.81 | 2.17 | Point | Democratic Republic of the Congo | Human | 171 |
| 18 | 18.37 | 0.04 | Point | Democratic Republic of the Congo | Human | 171 |
| 19 | 17.87 | -2.46 | Point | Democratic Republic of the Congo | Human | 171 |
| 20 | 17.67 | -2.87 | Point | Democratic Republic of the Congo | Human | 171 |
| 21 | 18.56 | -0.78 | Point | Democratic Republic of the Congo | Human | 171 |
| 22 | 18.62 | -1.17 | Point | Democratic Republic of the Congo | Human | 171 |
| 23 | 18.83 | -1.12 | Point | Democratic Republic of the Congo | Human | 171 |
| 24 | 18.47 | -4.23 | Point | Democratic Republic of the Congo | Human | 171 |
| 25 | 18.08 | -4.59 | Point | Democratic Republic of the Congo | Human | 171 |
| 26 | 19.33 | -4.62 | Point | Democratic Republic of the Congo | Human | 171 |
| 27 | 18.59 | -5.40 | Point | Democratic Republic of the Congo | Human | 171 |
| 28 | 18.47 | -6.34 | Point | Democratic Republic of the Congo | Human | 171 |
| 29 | 18.23 | -7.52 | Point | Democratic Republic of the Congo | Human | 171 |
| 30 | 17.81 | -6.80 | Point | Democratic Republic of the Congo | Human | 171 |
| 31 | 17.40 | -6.54 | Point | Democratic Republic of the Congo | Human | 171 |
| 32 | 17.10 | -5.94 | Point | Democratic Republic of the Congo | Human | 171 |
| 33 | 16.72 | -5.79 | Point | Democratic Republic of the Congo | Human | 171 |
| 34 | 20.04 | -5.22 | Point | Democratic Republic of the Congo | Human | 171 |
| 35 | 21.02 | -0.28 | Point | Democratic Republic of the Congo | Human | 171 |
| 36 | 22.19 | -1.27 | Point | Democratic Republic of the Congo | Human | 171 |
| 37 | 24.41 | -0.47 | Point | Democratic Republic of the Congo | Human | 171 |
| 38 | 24.27 | -2.14 | Point | Democratic Republic of the Congo | Human | 171 |
| 39 | 24.57 | -4.06 | Point | Democratic Republic of the Congo | Human | 171 |
| 40 | 21.35 | -4.03 | Point | Democratic Republic of the Congo | Human | 171 |
| 41 | 21.70 | -4.03 | Point | Democratic Republic of the Congo | Human | 171 |
| 42 | 23.01 | -4.22 | Point | Democratic Republic of the Congo | Human | 171 |
| 43 | 22.75 | -3.98 | Point | Democratic Republic of the Congo | Human | 171 |
| 44 | 22.31 | -3.71 | Point | Democratic Republic of the Congo | Human | 171 |
| 45 | 22.66 | -3.51 | Point | Democratic Republic of the Congo | Human | 171 |
| 46 | 22.40 | -3.35 | Point | Democratic Republic of the Congo | Human | 171 |
| 47 | 22.39 | -3.02 | Point | Democratic Republic of the Congo | Human | 171 |
| 48 | 22.90 | -1.41 | Point | Democratic Republic of the Congo | Human | 171 |
| 49 | 23.04 | -1.14 | Point | Democratic Republic of the Congo | Human | 171 |
| 50 | 23.49 | -1.14 | Point | Democratic Republic of the Congo | Human | 171 |
| 51 | 23.32 | -1.42 | Point | Democratic Republic of the Congo | Human | 171 |
| 52 | 22.68 | -0.70 | Point | Democratic Republic of the Congo | Human | 171 |
| 53 | 22.86 | -0.53 | Point | Democratic Republic of the Congo | Human | 171 |
| 54 | 20.72 | 0.50 | Point | Democratic Republic of the Congo | Human | 171 |
| 55 | 20.34 | 2.58 | Point | Democratic Republic of the Congo | Human | 171 |
| 56 | 20.39 | 2.79 | Point | Democratic Republic of the Congo | Human | 171 |
| 57 | 20.39 | 3.34 | Point | Democratic Republic of the Congo | Human | 171 |
| 58 | 23.41 | 2.44 | Point | Democratic Republic of the Congo | Human | 171 |
| 59 | 23.59 | 1.16 | Point | Democratic Republic of the Congo | Human | 171 |
| 60 | 22.51 | 0.53 | Point | Democratic Republic of the Congo | Human | 171 |
| 61 | 22.40 | 1.28 | Point | Democratic Republic of the Congo | Human | 171 |
| 62 | 21.68 | 2.37 | Point | Democratic Republic of the Congo | Human | 171 |
| 63 | 22.56 | 1.85 | Point | Democratic Republic of the Congo | Human | 171 |
| 64 | 22.75 | 2.12 | Point | Democratic Republic of the Congo | Human | 171 |
| 65 | 21.94 | 2.68 | Point | Democratic Republic of the Congo | Human | 171 |
| 66 | 26.65 | -0.53 | Point | Democratic Republic of the Congo | Human | 171 |
| 67 | 22.86 | 2.73 | Point | Democratic Republic of the Congo | Human | 171 |
| 68 | 22.64 | 3.09 | Point | Democratic Republic of the Congo | Human | 171 |
| 69 | 22.25 | 2.55 | Point | Democratic Republic of the Congo | Human | 171 |
| 70 | 22.56 | 2.77 | Point | Democratic Republic of the Congo | Human | 171 |
| 71 | 28.15 | 2.12 | Point | Democratic Republic of the Congo | Human | 171 |
| 72 | -1.18 | 7.65 | Point | Ghana | Human | 172 |
| 73 | -8.14 | 6.09 | Point | Liberia | Human | 172 |
| 74 | -7.82 | 6.17 | Point | Côte d'Ivoire | Human | 172 |
| 75 | -6.48 | 6.78 | Point | Côte d'Ivoire | Human | 172 |
| 76 | -3.43 | 6.60 | Point | Côte d'Ivoire | Human | 172 |
| 77 | 4.58 | 7.19 | Point | Nigeria | Human | 172 |
| 78 | 7.43 | 5.11 | Point | Nigeria | Human | 172 |
| 79 | 7.33 | 4.93 | Point | Nigeria | Human | 172 |
| 80 | 12.07 | 4.16 | Point | Cameroon | Human | 172 |
| 81 | 11.60 | 3.71 | Point | Cameroon | Human | 172 |
| 82 | 11.71 | 3.61 | Point | Cameroon | Human | 172 |
| 83 | 10.36 | -0.10 | Point | Gabon | Human | 172 |
| 84 | 10.34 | -0.39 | Point | Gabon | Human | 172 |
| 85 | 10.56 | -0.29 | Point | Gabon | Human | 172 |
| 86 | 16.21 | 2.68 | Point | Central African Republic | Human | 172 |
| 87 | 18.09 | 3.87 | Point | Central African Republic | Human | 172 |
| 88 | 18.73 | 3.66 | Point | Democratic Republic of the Congo | Human | 172 |
| 89 | 19.16 | 3.12 | Point | Democratic Republic of the Congo | Human | 172 |
| 90 | 18.07 | 2.04 | Point | Democratic Republic of the Congo | Human | 172 |
| 91 | 18.08 | 1.56 | Point | Democratic Republic of the Congo | Human | 172 |
| 92 | 18.50 | 1.34 | Point | Democratic Republic of the Congo | Human | 172 |
| 93 | 19.92 | 3.22 | Point | Democratic Republic of the Congo | Human | 172 |
| 94 | 20.44 | 3.31 | Point | Democratic Republic of the Congo | Human | 172 |
| 95 | 20.37 | 2.79 | Point | Democratic Republic of the Congo | Human | 172 |
| 96 | 20.09 | 2.62 | Point | Democratic Republic of the Congo | Human | 172 |
| 97 | 19.83 | 2.56 | Point | Democratic Republic of the Congo | Human | 172 |
| 98 | 20.33 | 2.22 | Point | Democratic Republic of the Congo | Human | 172 |
| 99 | 20.08 | 1.76 | Point | Democratic Republic of the Congo | Human | 172 |
| 100 | 20.38 | 1.75 | Point | Democratic Republic of the Congo | Human | 172 |
| 101 | 20.68 | 1.73 | Point | Democratic Republic of the Congo | Human | 172 |
| 102 | 21.18 | 1.46 | Point | Democratic Republic of the Congo | Human | 172 |
| 103 | 20.97 | 1.46 | Point | Democratic Republic of the Congo | Human | 172 |
| 104 | 19.92 | 1.19 | Point | Democratic Republic of the Congo | Human | 172 |
| 105 | 18.36 | -0.12 | Point | Democratic Republic of the Congo | Human | 172 |
| 106 | 18.06 | -0.19 | Point | Democratic Republic of the Congo | Human | 172 |
| 107 | 22.25 | 3.66 | Point | Democratic Republic of the Congo | Human | 172 |
| 108 | 22.50 | 4.18 | Point | Central African Republic | Human | 172 |
| 109 | 22.72 | 2.99 | Point | Democratic Republic of the Congo | Human | 172 |
| 110 | 22.83 | 2.72 | Point | Democratic Republic of the Congo | Human | 172 |
| 111 | 23.27 | 2.15 | Point | Democratic Republic of the Congo | Human | 172 |
| 112 | 22.80 | 2.15 | Point | Democratic Republic of the Congo | Human | 172 |
| 113 | 22.54 | 2.15 | Point | Democratic Republic of the Congo | Human | 172 |
| 114 | 22.48 | 2.46 | Point | Democratic Republic of the Congo | Human | 172 |
| 115 | 22.61 | 2.70 | Point | Democratic Republic of the Congo | Human | 172 |
| 116 | 22.40 | 2.95 | Point | Democratic Republic of the Congo | Human | 172 |
| 117 | 21.91 | 2.75 | Point | Democratic Republic of the Congo | Human | 172 |
| 118 | 21.82 | 2.51 | Point | Democratic Republic of the Congo | Human | 172 |
| 119 | 21.66 | 2.16 | Point | Democratic Republic of the Congo | Human | 172 |
| 120 | 21.84 | 1.95 | Point | Democratic Republic of the Congo | Human | 172 |
| 121 | 22.18 | 2.40 | Point | Democratic Republic of the Congo | Human | 172 |
| 122 | 22.23 | 2.69 | Point | Democratic Republic of the Congo | Human | 172 |
| 123 | 22.51 | 0.64 | Point | Democratic Republic of the Congo | Human | 172 |
| 124 | 21.40 | 0.43 | Point | Democratic Republic of the Congo | Human | 172 |
| 125 | 22.50 | 0.52 | Point | Democratic Republic of the Congo | Human | 172 |
| 126 | 20.90 | -0.51 | Point | Democratic Republic of the Congo | Human | 172 |
| 127 | 21.64 | -0.83 | Point | Democratic Republic of the Congo | Human | 172 |
| 128 | 18.77 | -1.19 | Point | Democratic Republic of the Congo | Human | 172 |
| 129 | 18.52 | -1.13 | Point | Democratic Republic of the Congo | Human | 172 |
| 130 | 18.48 | -0.84 | Point | Democratic Republic of the Congo | Human | 172 |
| 131 | 18.22 | -0.83 | Point | Democratic Republic of the Congo | Human | 172 |
| 132 | 18.42 | -1.66 | Point | Democratic Republic of the Congo | Human | 172 |
| 133 | 22.83 | -0.59 | Point | Democratic Republic of the Congo | Human | 172 |
| 134 | 22.83 | -0.93 | Point | Democratic Republic of the Congo | Human | 172 |
| 135 | 22.99 | -1.13 | Point | Democratic Republic of the Congo | Human | 172 |
| 136 | 23.42 | -1.23 | Point | Democratic Republic of the Congo | Human | 172 |
| 137 | 23.23 | -1.35 | Point | Democratic Republic of the Congo | Human | 172 |
| 138 | 23.24 | 1.21 | Point | Democratic Republic of the Congo | Human | 172 |
| 139 | 23.76 | 1.73 | Point | Democratic Republic of the Congo | Human | 172 |
| 140 | 23.70 | 1.12 | Point | Democratic Republic of the Congo | Human | 172 |
| 141 | 23.53 | 1.21 | Point | Democratic Republic of the Congo | Human | 172 |
| 142 | 23.87 | 2.69 | Point | Democratic Republic of the Congo | Human | 172 |
| 143 | 22.57 | 4.04 | Point | Democratic Republic of the Congo | Human | 172 |
| 144 | 25.69 | 3.24 | Point | Democratic Republic of the Congo | Human | 172 |
| 145 | 24.32 | -2.12 | Point | Democratic Republic of the Congo | Human | 172 |
| 146 | 23.39 | -2.36 | Point | Democratic Republic of the Congo | Human | 172 |
| 147 | 23.29 | -2.48 | Point | Democratic Republic of the Congo | Human | 172 |
| 148 | 24.56 | -3.47 | Point | Democratic Republic of the Congo | Human | 172 |
| 149 | 24.25 | -3.39 | Point | Democratic Republic of the Congo | Human | 172 |
| 150 | 24.26 | -3.55 | Point | Democratic Republic of the Congo | Human | 172 |
| 151 | 23.48 | -3.50 | Point | Democratic Republic of the Congo | Human | 172 |
| 152 | 24.56 | -4.78 | Point | Democratic Republic of the Congo | Human | 172 |
| 153 | 22.94 | -4.08 | Point | Democratic Republic of the Congo | Human | 172 |
| 154 | 22.65 | -4.23 | Point | Democratic Republic of the Congo | Human | 172 |
| 155 | 22.67 | -4.00 | Point | Democratic Republic of the Congo | Human | 172 |
| 156 | 22.54 | -3.85 | Point | Democratic Republic of the Congo | Human | 172 |
| 157 | 22.33 | -3.70 | Point | Democratic Republic of the Congo | Human | 172 |
| 158 | 22.46 | -3.46 | Point | Democratic Republic of the Congo | Human | 172 |
| 159 | 22.42 | -3.21 | Point | Democratic Republic of the Congo | Human | 172 |
| 160 | 22.25 | -3.16 | Point | Democratic Republic of the Congo | Human | 172 |
| 161 | 21.66 | -3.95 | Point | Democratic Republic of the Congo | Human | 172 |
| 162 | 21.42 | -4.11 | Point | Democratic Republic of the Congo | Human | 172 |
| 163 | 20.83 | -2.87 | Point | Democratic Republic of the Congo | Human | 172 |
| 164 | 18.49 | -3.16 | Point | Democratic Republic of the Congo | Human | 172 |
| 165 | 17.83 | -2.51 | Point | Democratic Republic of the Congo | Human | 172 |
| 166 | 17.91 | -3.15 | Point | Democratic Republic of the Congo | Human | 172 |
| 167 | 17.46 | -3.39 | Point | Democratic Republic of the Congo | Human | 172 |
| 168 | 18.39 | -4.26 | Point | Democratic Republic of the Congo | Human | 172 |
| 169 | 19.48 | -4.40 | Point | Democratic Republic of the Congo | Human | 172 |
| 170 | 18.97 | -4.54 | Point | Democratic Republic of the Congo | Human | 172 |
| 171 | 18.68 | -4.65 | Point | Democratic Republic of the Congo | Human | 172 |
| 172 | 18.01 | -4.84 | Point | Democratic Republic of the Congo | Human | 172 |
| 173 | 17.83 | -4.63 | Point | Democratic Republic of the Congo | Human | 172 |
| 174 | 18.93 | -5.12 | Point | Democratic Republic of the Congo | Human | 172 |
| 175 | 19.67 | -5.00 | Point | Democratic Republic of the Congo | Human | 172 |
| 176 | 20.04 | -4.97 | Point | Democratic Republic of the Congo | Human | 172 |
| 177 | 18.39 | -5.50 | Point | Democratic Republic of the Congo | Human | 172 |
| 178 | 18.45 | -6.29 | Point | Democratic Republic of the Congo | Human | 172 |
| 179 | 18.06 | -7.30 | Point | Democratic Republic of the Congo | Human | 172 |
| 180 | 17.50 | -6.39 | Point | Democratic Republic of the Congo | Human | 172 |
| 181 | 16.89 | -6.52 | Point | Democratic Republic of the Congo | Human | 172 |
| 182 | 17.16 | -5.91 | Point | Democratic Republic of the Congo | Human | 172 |
| 183 | 16.67 | -5.79 | Point | Democratic Republic of the Congo | Human | 172 |
| 184 | 16.66 | -4.11 | Point | Democratic Republic of the Congo | Human | 172 |
| 185 | 15.34 | -4.39 | Point | Democratic Republic of the Congo | Human | 172 |
| 186 | 16.35 | -2.92 | Point | Democratic Republic of the Congo | Human | 172 |
| 187 | 22.33 | -5.51 | Point | Democratic Republic of the Congo | Human | 172 |
| 188 | 28.85 | -2.53 | Point | Democratic Republic of the Congo | Human | 172 |
| 189 | -1.24 | 8.17 | Point | Ghana | Human | 173 |
| 190 | -8.13 | 6.07 | Point | Liberia | Human | 173 |
| 191 | -6.45 | 6.81 | Point | Côte d'Ivoire | Human | 173 |
| 192 | -0.05 | 5.90 | Point | Ghana | Human | 173 |
| 193 | 4.57 | 7.16 | Point | Nigeria | Human | 173 |
| 194 | 7.31 | 5.01 | Point | Nigeria | Human | 173 |
| 195 | 11.51 | 4.12 | Point | Cameroon | Human | 173 |
| 196 | 10.65 | -0.26 | Point | Gabon | Human | 173 |
| 197 | 18.05 | 1.62 | Point | Republic of Congo | Human | 173 |
| 198 | 19.79 | 1.22 | Point | Democratic Republic of the Congo | Human | 173 |
| 199 | 22.20 | 2.80 | Point | Democratic Republic of the Congo | Human | 173 |
| 200 | 20.91 | -0.24 | Point | Democratic Republic of the Congo | Human | 173 |
| 201 | 23.84 | -2.26 | Point | Democratic Republic of the Congo | Human | 173 |
| 202 | 21.94 | -2.47 | Point | Democratic Republic of the Congo | Human | 173 |
| 203 | 23.97 | -3.25 | Point | Democratic Republic of the Congo | Human | 173 |
| 204 | 24.15 | -3.23 | Point | Democratic Republic of the Congo | Human | 173 |
| 205 | 23.49 | -3.89 | Point | Democratic Republic of the Congo | Human | 173 |
| 206 | 22.74 | -3.71 | Point | Democratic Republic of the Congo | Human | 173 |
| 207 | 22.26 | -3.71 | Point | Democratic Republic of the Congo | Human | 173 |
| 208 | 19.20 | -4.57 | Point | Democratic Republic of the Congo | Human | 173 |
| 209 | 24.74 | -3.04 | Point | Democratic Republic of the Congo | Human | 173 |
| 210 | 2.83 | 6.42 | Point | Nigeria | Human | 112 |
| 211 | 2.99 | 6.45 | Point | Nigeria | Human | 112 |
| 212 | 2.98 | 6.51 | Point | Nigeria | Human | 112 |
| 213 | 3.05 | 6.48 | Point | Nigeria | Human | 112 |
| 214 | 3.11 | 6.43 | Point | Nigeria | Human | 112 |
| 215 | 3.26 | 6.56 | Point | Nigeria | Human | 112 |
| 216 | 3.33 | 6.53 | Point | Nigeria | Human | 112 |
| 217 | 3.26 | 6.42 | Point | Nigeria | Human | 112 |
| 218 | 3.43 | 6.51 | Point | Nigeria | Human | 112 |
| 219 | 3.41 | 6.45 | Point | Nigeria | Human | 112 |
| 220 | 3.48 | 6.45 | Point | Nigeria | Human | 112 |
| 221 | 3.61 | 6.46 | Point | Nigeria | Human | 112 |
| 222 | 3.59 | 6.55 | Point | Nigeria | Human | 112 |
| 223 | 3.51 | 6.59 | Point | Nigeria | Human | 112 |
| 224 | 3.45 | 6.60 | Point | Nigeria | Human | 112 |
| 225 | 3.53 | 6.64 | Point | Nigeria | Human | 112 |
| 226 | 3.59 | 6.68 | Point | Nigeria | Human | 112 |
| 227 | 3.65 | 6.64 | Point | Nigeria | Human | 112 |
| 228 | 3.73 | 6.61 | Point | Nigeria | Human | 112 |
| 229 | 3.81 | 6.61 | Point | Nigeria | Human | 112 |
| 230 | 4.23 | 6.43 | Point | Nigeria | Human | 112 |
| 231 | 4.15 | 6.50 | Point | Nigeria | Human | 112 |
| 232 | 4.04 | 6.49 | Point | Nigeria | Human | 112 |
| 233 | 3.99 | 6.56 | Point | Nigeria | Human | 112 |
| 234 | 4.04 | 6.59 | Point | Nigeria | Human | 112 |
| 235 | 4.02 | 6.66 | Point | Nigeria | Human | 112 |
| 236 | 3.96 | 6.69 | Point | Nigeria | Human | 112 |
| 237 | 4.03 | 6.76 | Point | Nigeria | Human | 112 |
| 238 | 3.21 | 8.12 | Point | Nigeria | Human | 112 |
| 239 | 3.63 | 7.82 | Point | Nigeria | Human | 112 |
| 240 | 3.54 | 8.54 | Point | Nigeria | Human | 112 |
| 241 | 3.37 | 8.66 | Point | Nigeria | Human | 112 |
| 242 | 3.93 | 8.44 | Point | Nigeria | Human | 112 |
| 243 | 4.12 | 7.98 | Point | Nigeria | Human | 112 |
| 244 | 5.26 | 9.97 | Point | Nigeria | Human | 112 |
| 245 | 5.40 | 7.88 | Point | Nigeria | Human | 112 |
| 246 | 5.47 | 7.46 | Point | Nigeria | Human | 112 |
| 247 | 6.15 | 7.32 | Point | Nigeria | Human | 112 |
| 248 | 6.01 | 6.95 | Point | Nigeria | Human | 112 |
| 249 | 6.52 | 6.87 | Point | Nigeria | Human | 112 |
| 250 | 6.65 | 6.53 | Point | Nigeria | Human | 112 |
| 251 | 6.56 | 6.48 | Point | Nigeria | Human | 112 |
| 252 | 6.28 | 6.46 | Point | Nigeria | Human | 112 |
| 253 | 6.49 | 6.30 | Point | Nigeria | Human | 112 |
| 254 | 6.20 | 6.18 | Point | Nigeria | Human | 112 |
| 255 | 6.50 | 6.08 | Point | Nigeria | Human | 112 |
| 256 | 6.68 | 6.02 | Point | Nigeria | Human | 112 |
| 257 | 6.52 | 5.87 | Point | Nigeria | Human | 112 |
| 258 | 6.36 | 5.93 | Point | Nigeria | Human | 112 |
| 259 | 6.95 | 6.24 | Point | Nigeria | Human | 112 |
| 260 | 5.64 | 6.44 | Point | Nigeria | Human | 112 |
| 261 | 5.80 | 6.33 | Point | Nigeria | Human | 112 |
| 262 | 5.33 | 6.23 | Point | Nigeria | Human | 112 |
| 263 | 5.86 | 5.97 | Point | Nigeria | Human | 112 |
| 264 | 5.75 | 5.84 | Point | Nigeria | Human | 112 |
| 265 | 5.64 | 5.69 | Point | Nigeria | Human | 112 |
| 266 | 5.47 | 5.81 | Point | Nigeria | Human | 112 |
| 267 | 5.25 | 5.67 | Point | Nigeria | Human | 112 |
| 268 | 5.85 | 5.68 | Point | Nigeria | Human | 112 |
| 269 | 5.90 | 5.73 | Point | Nigeria | Human | 112 |
| 270 | 5.93 | 5.59 | Point | Nigeria | Human | 112 |
| 271 | 5.39 | 5.50 | Point | Nigeria | Human | 112 |
| 272 | 5.48 | 5.48 | Point | Nigeria | Human | 112 |
| 273 | 5.63 | 5.48 | Point | Nigeria | Human | 112 |
| 274 | 5.58 | 5.42 | Point | Nigeria | Human | 112 |
| 275 | 5.54 | 5.31 | Point | Nigeria | Human | 112 |
| 276 | 5.66 | 5.31 | Point | Nigeria | Human | 112 |
| 277 | 5.70 | 5.41 | Point | Nigeria | Human | 112 |
| 278 | 5.87 | 5.34 | Point | Nigeria | Human | 112 |
| 279 | 5.98 | 5.26 | Point | Nigeria | Human | 112 |
| 280 | 5.62 | 4.93 | Point | Nigeria | Human | 112 |
| 281 | 5.77 | 4.91 | Point | Nigeria | Human | 112 |
| 282 | 5.99 | 4.93 | Point | Nigeria | Human | 112 |
| 283 | 5.60 | 4.80 | Point | Nigeria | Human | 112 |
| 284 | 5.77 | 4.76 | Point | Nigeria | Human | 112 |
| 285 | 5.87 | 4.72 | Point | Nigeria | Human | 112 |
| 286 | 5.77 | 4.67 | Point | Nigeria | Human | 112 |
| 287 | 6.10 | 5.03 | Point | Nigeria | Human | 112 |
| 288 | 6.17 | 5.05 | Point | Nigeria | Human | 112 |
| 289 | 6.34 | 5.16 | Point | Nigeria | Human | 112 |
| 290 | 6.37 | 5.12 | Point | Nigeria | Human | 112 |
| 291 | 6.42 | 5.15 | Point | Nigeria | Human | 112 |
| 292 | 6.40 | 5.31 | Point | Nigeria | Human | 112 |
| 293 | 6.18 | 4.99 | Point | Nigeria | Human | 112 |
| 294 | 6.24 | 4.93 | Point | Nigeria | Human | 112 |
| 295 | 6.28 | 4.95 | Point | Nigeria | Human | 112 |
| 296 | 6.34 | 4.88 | Point | Nigeria | Human | 112 |
| 297 | 6.20 | 4.84 | Point | Nigeria | Human | 112 |
| 298 | 6.12 | 4.81 | Point | Nigeria | Human | 112 |
| 299 | 6.24 | 4.80 | Point | Nigeria | Human | 112 |
| 300 | 6.21 | 4.68 | Point | Nigeria | Human | 112 |
| 301 | 6.15 | 4.68 | Point | Nigeria | Human | 112 |
| 302 | 6.17 | 4.61 | Point | Nigeria | Human | 112 |
| 303 | 6.14 | 4.56 | Point | Nigeria | Human | 112 |
| 304 | 6.09 | 4.60 | Point | Nigeria | Human | 112 |
| 305 | 5.98 | 4.60 | Point | Nigeria | Human | 112 |
| 306 | 5.94 | 4.62 | Point | Nigeria | Human | 112 |
| 307 | 5.98 | 4.67 | Point | Nigeria | Human | 112 |
| 308 | 5.81 | 4.50 | Point | Nigeria | Human | 112 |
| 309 | 5.94 | 4.52 | Point | Nigeria | Human | 112 |
| 310 | 5.99 | 4.45 | Point | Nigeria | Human | 112 |
| 311 | 6.09 | 4.35 | Point | Nigeria | Human | 112 |
| 312 | 6.38 | 4.43 | Point | Nigeria | Human | 112 |
| 313 | 6.45 | 4.41 | Point | Nigeria | Human | 112 |
| 314 | 6.55 | 4.40 | Point | Nigeria | Human | 112 |
| 315 | 6.66 | 4.47 | Point | Nigeria | Human | 112 |
| 316 | 6.77 | 4.40 | Point | Nigeria | Human | 112 |
| 317 | 6.81 | 4.49 | Point | Nigeria | Human | 112 |
| 318 | 6.85 | 4.45 | Point | Nigeria | Human | 112 |
| 319 | 6.67 | 4.74 | Point | Nigeria | Human | 112 |
| 320 | 6.57 | 4.61 | Point | Nigeria | Human | 112 |
| 321 | 6.52 | 4.60 | Point | Nigeria | Human | 112 |
| 322 | 6.43 | 4.59 | Point | Nigeria | Human | 112 |
| 323 | 6.52 | 4.67 | Point | Nigeria | Human | 112 |
| 324 | 6.59 | 4.66 | Point | Nigeria | Human | 112 |
| 325 | 6.48 | 4.72 | Point | Nigeria | Human | 112 |
| 326 | 6.40 | 4.81 | Point | Nigeria | Human | 112 |
| 327 | 6.51 | 4.92 | Point | Nigeria | Human | 112 |
| 328 | 6.57 | 4.80 | Point | Nigeria | Human | 112 |
| 329 | 6.64 | 5.02 | Point | Nigeria | Human | 112 |
| 330 | 6.71 | 5.05 | Point | Nigeria | Human | 112 |
| 331 | 6.71 | 5.12 | Point | Nigeria | Human | 112 |
| 332 | 6.74 | 5.21 | Point | Nigeria | Human | 112 |
| 333 | 6.81 | 5.14 | Point | Nigeria | Human | 112 |
| 334 | 6.80 | 5.06 | Point | Nigeria | Human | 112 |
| 335 | 6.81 | 4.98 | Point | Nigeria | Human | 112 |
| 336 | 6.92 | 4.92 | Point | Nigeria | Human | 112 |
| 337 | 6.82 | 4.87 | Point | Nigeria | Human | 112 |
| 338 | 6.90 | 4.78 | Point | Nigeria | Human | 112 |
| 339 | 6.98 | 4.76 | Point | Nigeria | Human | 112 |
| 340 | 7.04 | 4.70 | Point | Nigeria | Human | 112 |
| 341 | 7.17 | 4.86 | Point | Nigeria | Human | 112 |
| 342 | 7.13 | 4.84 | Point | Nigeria | Human | 112 |
| 343 | 7.21 | 4.84 | Point | Nigeria | Human | 112 |
| 344 | 7.15 | 4.92 | Point | Nigeria | Human | 112 |
| 345 | 7.13 | 5.02 | Point | Nigeria | Human | 112 |
| 346 | 7.03 | 5.04 | Point | Nigeria | Human | 112 |
| 347 | 6.98 | 5.11 | Point | Nigeria | Human | 112 |
| 348 | 6.92 | 5.19 | Point | Nigeria | Human | 112 |
| 349 | 6.74 | 5.33 | Point | Nigeria | Human | 112 |
| 350 | 6.95 | 4.56 | Point | Nigeria | Human | 112 |
| 351 | 7.29 | 4.84 | Point | Nigeria | Human | 112 |
| 352 | 7.29 | 4.75 | Point | Nigeria | Human | 112 |
| 353 | 7.22 | 4.69 | Point | Nigeria | Human | 112 |
| 354 | 7.34 | 4.71 | Point | Nigeria | Human | 112 |
| 355 | 7.29 | 4.63 | Point | Nigeria | Human | 112 |
| 356 | 7.26 | 4.59 | Point | Nigeria | Human | 112 |
| 357 | 7.27 | 4.50 | Point | Nigeria | Human | 112 |
| 358 | 7.31 | 4.51 | Point | Nigeria | Human | 112 |
| 359 | 7.50 | 4.67 | Point | Nigeria | Human | 112 |
| 360 | 7.55 | 4.73 | Point | Nigeria | Human | 112 |
| 361 | 7.61 | 5.05 | Point | Nigeria | Human | 112 |
| 362 | 7.30 | 5.16 | Point | Nigeria | Human | 112 |
| 363 | 7.25 | 5.19 | Point | Nigeria | Human | 112 |
| 364 | 7.63 | 5.19 | Point | Nigeria | Human | 112 |
| 365 | 7.66 | 5.21 | Point | Nigeria | Human | 112 |
| 366 | 7.75 | 5.19 | Point | Nigeria | Human | 112 |
| 367 | 7.18 | 5.40 | Point | Nigeria | Human | 112 |
| 368 | 7.00 | 5.37 | Point | Nigeria | Human | 112 |
| 369 | 6.95 | 5.37 | Point | Nigeria | Human | 112 |
| 370 | 6.82 | 5.43 | Point | Nigeria | Human | 112 |
| 371 | 6.66 | 5.42 | Point | Nigeria | Human | 112 |
| 372 | 6.64 | 5.51 | Point | Nigeria | Human | 112 |
| 373 | 6.58 | 5.56 | Point | Nigeria | Human | 112 |
| 374 | 6.91 | 5.72 | Point | Nigeria | Human | 112 |
| 375 | 6.78 | 5.80 | Point | Nigeria | Human | 112 |
| 376 | 7.26 | 5.87 | Point | Nigeria | Human | 112 |
| 377 | 7.47 | 5.61 | Point | Nigeria | Human | 112 |
| 378 | 7.52 | 5.71 | Point | Nigeria | Human | 112 |
| 379 | 7.72 | 5.66 | Point | Nigeria | Human | 112 |
| 380 | 7.95 | 4.84 | Point | Nigeria | Human | 112 |
| 381 | 8.03 | 4.96 | Point | Nigeria | Human | 112 |
| 382 | 8.15 | 4.76 | Point | Nigeria | Human | 112 |
| 383 | 8.38 | 5.16 | Point | Nigeria | Human | 112 |
| 384 | 8.07 | 5.55 | Point | Nigeria | Human | 112 |
| 385 | 7.46 | 6.08 | Point | Nigeria | Human | 112 |
| 386 | 7.55 | 6.35 | Point | Nigeria | Human | 112 |
| 387 | 8.00 | 6.28 | Point | Nigeria | Human | 112 |
| 388 | 7.21 | 6.68 | Point | Nigeria | Human | 112 |
| 389 | 7.49 | 6.84 | Point | Nigeria | Human | 112 |
| 390 | 8.58 | 6.75 | Point | Nigeria | Human | 112 |
| 391 | 8.83 | 6.75 | Point | Nigeria | Human | 112 |
| 392 | 8.92 | 6.65 | Point | Nigeria | Human | 112 |
| 393 | 8.89 | 6.54 | Point | Nigeria | Human | 112 |
| 394 | 8.70 | 6.56 | Point | Nigeria | Human | 112 |
| 395 | 8.91 | 6.23 | Point | Nigeria | Human | 112 |
| 396 | 9.10 | 6.23 | Point | Nigeria | Human | 112 |
| 397 | 8.61 | 6.00 | Point | Nigeria | Human | 112 |
| 398 | 8.76 | 5.78 | Point | Nigeria | Human | 112 |
| 399 | 8.65 | 5.47 | Point | Nigeria | Human | 112 |
| 400 | 8.66 | 5.25 | Point | Nigeria | Human | 112 |
| 401 | 8.65 | 11.31 | Point | Nigeria | Human | 112 |
| 402 | 10.03 | 9.33 | Point | Nigeria | Human | 112 |
| 403 | 9.04 | 9.42 | Point | Nigeria | Human | 112 |
| 404 | 8.44 | 8.77 | Point | Nigeria | Human | 112 |
| 405 | 9.58 | 8.77 | Point | Nigeria | Human | 112 |
| 406 | 7.48 | 9.14 | Point | Nigeria | Human | 112 |
| 407 | 7.48 | 9.02 | Point | Nigeria | Human | 112 |
| 408 | 7.36 | 8.92 | Point | Nigeria | Human | 112 |
| 409 | 7.20 | 8.56 | Point | Nigeria | Human | 112 |
| 410 | 6.93 | 8.57 | Point | Nigeria | Human | 112 |
| 411 | 6.91 | 8.83 | Point | Nigeria | Human | 112 |
| 412 | 6.93 | 9.10 | Point | Nigeria | Human | 112 |
| 413 | 7.85 | 8.21 | Point | Nigeria | Human | 112 |
| 414 | 9.01 | 7.63 | Point | Nigeria | Human | 112 |
| 415 | 8.53 | 7.44 | Point | Nigeria | Human | 112 |
| 416 | 12.41 | 8.92 | Point | Nigeria | Human | 112 |
| 417 | 12.08 | 8.93 | Point | Nigeria | Human | 112 |
| 418 | 12.56 | 9.78 | Point | Nigeria | Human | 112 |
| 419 | 6.32 | 5.48 | Point | Nigeria | Human | 112 |
| 420 | 6.23 | 5.52 | Point | Nigeria | Human | 112 |
| 421 | 6.26 | 5.69 | Point | Nigeria | Human | 112 |
| 422 | 6.15 | 5.77 | Point | Nigeria | Human | 112 |
| 423 | 22.73 | 5.74 | Point | Central African Republic | Human | 112 |
| 424 | 22.76 | 5.70 | Point | Central African Republic | Human | 112 |
| 425 | 22.80 | 5.70 | Point | Central African Republic | Human | 112 |
| 426 | 22.85 | 5.68 | Point | Central African Republic | Human | 112 |
| 427 | 22.86 | 5.70 | Point | Central African Republic | Human | 112 |
| 428 | 22.88 | 5.68 | Point | Central African Republic | Human | 112 |
| 429 | 22.86 | 5.66 | Point | Central African Republic | Human | 112 |
| 430 | 22.95 | 4.90 | Point | Central African Republic | Human | 112 |
| 431 | 22.81 | 4.77 | Point | Central African Republic | Human | 112 |
| 432 | 22.83 | 4.77 | Point | Central African Republic | Human | 112 |
| 433 | 22.81 | 4.74 | Point | Central African Republic | Human | 112 |
| 434 | 22.84 | 4.73 | Point | Central African Republic | Human | 112 |
| 435 | 19.65 | 3.35 | Point | Democratic Republic of the Congo | Human | 112 |
| 436 | 19.63 | 3.11 | Point | Democratic Republic of the Congo | Human | 112 |
| 437 | 20.71 | 1.94 | Point | Democratic Republic of the Congo | Human | 112 |
| 438 | 20.29 | 1.00 | Point | Democratic Republic of the Congo | Human | 112 |
| 439 | 21.71 | 2.83 | Point | Democratic Republic of the Congo | Human | 112 |
| 440 | 21.67 | 2.61 | Point | Democratic Republic of the Congo | Human | 112 |
| 441 | 21.66 | 2.46 | Point | Democratic Republic of the Congo | Human | 112 |
| 442 | 21.88 | 2.30 | Point | Democratic Republic of the Congo | Human | 112 |
| 443 | 22.01 | 2.38 | Point | Democratic Republic of the Congo | Human | 112 |
| 444 | 22.13 | 2.46 | Point | Democratic Republic of the Congo | Human | 112 |
| 445 | 22.12 | 2.28 | Point | Democratic Republic of the Congo | Human | 112 |
| 446 | 22.24 | 2.32 | Point | Democratic Republic of the Congo | Human | 112 |
| 447 | 22.32 | 2.17 | Point | Democratic Republic of the Congo | Human | 112 |
| 448 | 22.32 | 0.62 | Point | Democratic Republic of the Congo | Human | 112 |
| 449 | 23.11 | -1.14 | Point | Democratic Republic of the Congo | Human | 112 |
| 450 | 17.82 | -2.45 | Point | Democratic Republic of the Congo | Human | 112 |
| 451 | 23.57 | -2.85 | Point | Democratic Republic of the Congo | Human | 112 |
| 452 | 22.11 | -2.67 | Point | Democratic Republic of the Congo | Human | 112 |
| 453 | 22.20 | -3.32 | Point | Democratic Republic of the Congo | Human | 112 |
| 454 | 22.29 | -3.49 | Point | Democratic Republic of the Congo | Human | 112 |
| 455 | 22.50 | -3.48 | Point | Democratic Republic of the Congo | Human | 112 |
| 456 | 22.22 | -3.60 | Point | Democratic Republic of the Congo | Human | 112 |
| 457 | 22.42 | -3.74 | Point | Democratic Republic of the Congo | Human | 112 |
| 458 | 27.91 | -1.53 | Point | Democratic Republic of the Congo | Human | 112 |
| 459 | 17.91 | -2.75 | Point | Democratic Republic of the Congo | Human | 112 |
| 460 | 18.70 | -3.75 | Point | Democratic Republic of the Congo | Human | 112 |
| 461 | 17.64 | -4.72 | Point | Democratic Republic of the Congo | Human | 112 |
| 462 | 17.09 | -5.40 | Point | Democratic Republic of the Congo | Human | 112 |
| 463 | 17.82 | -5.72 | Point | Democratic Republic of the Congo | Human | 112 |
| 464 | 18.24 | -6.39 | Point | Democratic Republic of the Congo | Human | 112 |
| 465 | 8.41 | 4.89 | Point | Nigeria | Human | 112 |
| 466 | 7.38 | 4.96 | Point | Nigeria | Human | 112 |
| 467 | 3.63 | 7.28 | Point | Nigeria | Human | 112 |
| 468 | -3.38 | 6.29 | Point | Côte d'Ivoire | Human | 112 |
| 469 | -7.65 | 5.15 | Point | Liberia | Human | 112 |
| 470 | -8.06 | 5.84 | Point | Liberia | Human | 112 |
| 471 | -8.49 | 5.22 | Point | Liberia | Human | 112 |
| 472 | -9.44 | 6.06 | Point | Liberia | Human | 112 |
| 473 | -1.13 | 8.09 | Point | Ghana | Human | 112 |
| 474 | 22.17 | -5.51 | Point | Democratic Republic of the Congo | Human | 112 |
| 475 | 24.26 | -4.39 | Point | Democratic Republic of the Congo | Human | 112 |
| 476 | 22.25 | -2.55 | Point | Democratic Republic of the Congo | Human | 112 |
| 477 | 18.99 | -4.85 | Point | Democratic Republic of the Congo | Human | 112 |
| 478 | 17.81 | -5.99 | Point | Democratic Republic of the Congo | Human | 112 |
| 479 | 22.47 | -0.77 | Point | Democratic Republic of the Congo | Human | 112 |
| 480 | 18.21 | -1.94 | Point | Democratic Republic of the Congo | Human | 112 |
| 481 | 26.36 | -1.71 | Point | Democratic Republic of the Congo | Human | 112 |
| 482 | 28.43 | -2.38 | Point | Democratic Republic of the Congo | Human | 112 |
| 483 | 19.89 | 1.05 | Point | Democratic Republic of the Congo | Human | 112 |
| 484 | 22.69 | 2.11 | Point | Democratic Republic of the Congo | Human | 112 |
| 485 | 25.10 | 2.97 | Point | Democratic Republic of the Congo | Human | 112 |
| 486 | 20.53 | 3.77 | Point | Democratic Republic of the Congo | Human | 112 |
| 487 | 19.35 | 3.47 | Point | Democratic Republic of the Congo | Human | 112 |
| 488 | 13.59 | 3.08 | Point | Cameroon | Human | 112 |
| 489 | 7.01 | 4.82 | Point | Nigeria | Human | 112 |
| 490 | 3.87 | 7.45 | Point | Nigeria | Human | 112 |
| 491 | -4.20 | 6.48 | Point | Côte d'Ivoire | Human | 112 |
| 492 | -8.52 | 5.93 | Point | Liberia | Human | 112 |
| 493 | -1.19 | 7.62 | Point | Ghana | Human | 112 |
| 494 | 7.35 | 8.96 | polygon | Nigeria | Human | 6 |
| 495 | 7.03 | 4.75 | polygon | Nigeria | Human | 10 |
| 496 | 23.22 | 5.06 | polygon | Central African Republic | Human | 11 |
| 497 | 18.18 | 2.55 | point | Republic of Congo | Human | 14 |
| 498 | 18.48 | 3.17 | point | Republic of Congo | Human | 14 |
| 499 | 22.70 | 3.57 | point | Democratic Republic of the Congo | Human | 20 |
| 500 | 20.97 | 1.70 | point | Democratic Republic of the Congo | Human | 20 |
| 501 | -11.56 | 7.28 | polygon | Sierra Leone | Human | 24 |
| 502 | 17.80 | 0.86 | polygon | Republic of Congo | Human | 28 |
| 503 | 3.56 | 6.53 | polygon | Nigeria | Human | 32 |
| 504 | 23.46 | -3.51 | polygon | Democratic Republic of the Congo | Human | 47 |
| 505 | 6.11 | 4.77 | polygon | Nigeria | Human | 51 |
| 506 | 9.86 | 6.14 | polygon | Cameroon | Human | 58 |
| 507 | 27.63 | -1.33 | point | Democratic Republic of the Congo | Human | 64 |
| 508 | 29.36 | -5.59 | point | Democratic Republic of the Congo | Human | 64 |
| 509 | 16.89 | -5.09 | point | Democratic Republic of the Congo | Human | 64 |
| 510 | -11.62 | 7.32 | polygon | Sierra Leone | Human | 79 |
| 511 | -11.76 | 8.24 | point | Sierra Leone | Human | 79 |
| 512 | 25.69 | -9.73 | point | Democratic Republic of the Congo | Human | 85 |
| 513 | 22.63 | -0.60 | point | Democratic Republic of the Congo | Human | 89 |
| 514 | -8.21 | 5.94 | polygon | Liberia | Human | 89 |
| 515 | -9.82 | 5.80 | point | Liberia | Human | 89 |
| 516 | -12.36 | 8.04 | polygon | Sierra Leone | Human | 89 |
| 517 | 7.51 | 5.45 | polygon | Nigeria | Human | 89 |
| 518 | -3.50 | 6.67 | polygon | Côte d'Ivoire | Human | 89 |
| 519 | 22.11 | 2.49 | point | Democratic Republic of the Congo | Human | 89 |
| 520 | 16.50 | -5.33 | point | Democratic Republic of the Congo | Human | 89 |
| 521 | 14.01 | -5.05 | point | Democratic Republic of the Congo | Human | 89 |
| 522 | 18.94 | 4.67 | point | Democratic Republic of the Congo | Human | 89 |
| 523 | 21.81 | 2.77 | point | Democratic Republic of the Congo | Human | 89 |
| 524 | 22.47 | 2.18 | point | Democratic Republic of the Congo | Human | 89 |
| 525 | 15.40 | -4.38 | point | Democratic Republic of the Congo | Human | 89 |
| 526 | 17.99 | -4.52 | point | Democratic Republic of the Congo | Human | 89 |
| 527 | 19.36 | -4.70 | point | Democratic Republic of the Congo | Human | 89 |
| 528 | 14.92 | -0.24 | point | Republic of Congo | Human | 89 |
| 529 | 21.97 | -2.45 | point | Democratic Republic of the Congo | Human | 89 |
| 530 | 4.60 | 7.17 | point | Nigeria | Human | 89 |
| 531 | -7.15 | 5.74 | point | Côte d'Ivoire | *Cercocebus atys atys* | 100 |
| 532 | -7.03 | 5.29 | point | Côte d'Ivoire | *Pan troglodytes* | 101 |
| 533 | -7.29 | 6.32 | point | Côte d'Ivoire | *Pan troglodytes* | 101 |
| 534 | -6.92 | 5.94 | point | Côte d'Ivoire | *Pan troglodytes* | 101 |
| 535 | -7.15 | 5.74 | point | Côte d'Ivoire | *Cercocebus atys atys*; chimpanzee; fly | 101 |
| 536 | 16.21 | 2.50 | polygon | Central African Republic | Human | 178 |
| 537 | -3.43 | 6.63 | point | Côte d'Ivoire | Human | 175 |
| 538 | 7.51 | 5.45 | polygon | Nigeria | Human | 174 |
| 539 | 22.45 | -3.46 | point | Democratic Republic of the Congo | Human | 179 |
| 540 | 11.58 | 3.63 | point | Cameroon | Mammal | 180 |
| 541 | 22.30 | 2.98 | point | Democratic Republic of the Congo | Human | 176 |
| 542 | 22.30 | 2.98 | point | Democratic Republic of the Congo | Mammal | 177 |
| 543 | 22.48 | 2.78 | point | Democratic Republic of the Congo | Mammal | 177 |
| 544 | 16.19 | 2.47 | polygon | Central African Republic | Human | 178 |
| 545 | -11.63 | 7.37 | point | Sierra Leone | Human | 181 |
| 546 | -11.67 | 7.96 | polygon | Sierra Leone | Human | 181 |
| 547 | 17.66 | 2.36 | point | Republic of Congo | Human | 181 |

# Appendix Table S20: Mean (95% percentiles) relative contributions of variables (RC≥3%) and mean AUCs (95% percentiles) of five BRT models in this study

| **Variable** | ***Cricetomys gambianus*** | ***Funisciurus* spp.** | ***Graphiurus crassicaudatus*** | ***Graphiurus lorraineus*** | **MPXV** |
| --- | --- | --- | --- | --- | --- |
| Environment |  |  |  |  |  |
| Annual mean temperature | 9.58 (7.09‒12.46) | 9.01 (7.28‒10.55) | 8.26 (2.95‒13.55) | ‒ | 6.90 (5.28‒8.48) |
| Mean diurnal range | 14.41 (10.05‒19.23) | 34.00 (29.78‒38.12) | ‒ | 6.09 (3.84‒9.00) | ‒ |
| Isothermality | 10.51 (8.21‒13.26) | 10.38 (8.74‒12.26) | ‒ | 4.00 (2.09‒6.69) | 9.27 (7.88‒10.67) |
| Min temperature of coldest month | ‒ | ‒ | 45.98 (26.06‒63.55) | 6.75 (2.61‒14.44) | 29.89 (22.14‒36.70) |
| Mean temperature of coldest quarter | 9.85 (7.00‒13.35) | ‒ | ‒ | ‒ | ‒ |
| Annual precipitation | 11.87 (9.31‒14.92) | 13.80 (11.50‒16.81) | 9.85 (4.49‒17.35) | 7.83 (4.95‒11.50) | 31.83 (26.24‒40.09) |
| Precipitation of driest month | ‒ | 7.06 (4.99‒9.57) | ‒ | 7.39 (3.55‒13.09) | ‒ |
| Precipitation of coldest quarter | ‒ | ‒ | ‒ | 27.87 (17.43‒38.05) | ‒ |
| Forest | ‒ | 3.56 (2.51‒4.69) | ‒ | 5.75 (2.92‒10.03) | ‒ |
| Shrubland | ‒ | ‒ | 7.78 (2.42‒14.41) | ‒ | ‒ |
| Mixed cropland and nature vegetation | 9.49 (6.74‒13.43) | 8.59 (6.62‒11.04) | ‒ | 25.54 (17.40‒36.91) | ‒ |
| Mixed tree, shrub and herbaceous | ‒ | 3.87 (2.52‒5.16) | ‒ | ‒ | ‒ |
| Water body | ‒ | 3.51 (2.63‒4.68) | ‒ | ‒ | ‒ |
| Human activity |  |  |  |  |  |
| Cropland | ‒ | 6.22 (4.62‒8.17) | ‒ | 8.79 (3.43‒15.79) | ‒ |
| Urbanland | ‒ | ‒ | 8.05 (0.91‒21.02) | ‒ | ‒ |
| Human footprint | 34.27 (29.87‒40.27) | ‒ | 20.08 (8.14‒40.83) | ‒ | ‒ |
| Animal |  |  |  |  |  |
| *Graphiurus crassicaudatus* | ‒ | ‒ | ‒ | ‒ | 6.51 (5.01‒8.10) |
| *Graphiurus lorraineus* | ‒ | ‒ | ‒ | ‒ | 8.02 (6.31‒9.78) |
| Rodentia richness | ‒ | ‒ | ‒ | ‒ | 7.57 (6.33‒8.75) |
| AUC | 0.93 (0.88‒0.96) | 0.90 (0.86‒0.92) | 0.96 (0.86‒1.00) | 0.88 (0.79‒0.96) | 0.95 (0.94‒0.97) |

# Appendix Table S21: Model-Predicted populations and areas at risk of MPXV infections in Africa

| **Country** | **Risk population (million)** | **Risk area (10 000 km^2^)** |
| --- | --- | --- |
| Endemic countries |  |  |
| Nigeria | 176.00 | 33.24 |
| Democratic Republic of the Congo | 81.88 | 127.11 |
| Ghana | 38.30 | 12.12 |
| Ivory Coast | 25.22 | 13.25 |
| Cameroon | 18.77 | 13.97 |
| Benin | 9.66 | 2.17 |
| Sierra Leone | 7.30 | 4.43 |
| Liberia | 5.79 | 8.83 |
| Gabon | 3.59 | 12.81 |
| Republic of Congo | 3.40 | 20.50 |
| Central African Republic | 3.36 | 10.13 |
| Potential endemic country |  |  |
| Ethiopia | 18.81 | 7.87 |
| Kenya | 6.92 | 1.53 |
| Guinea | 6.37 | 2.16 |
| Togo | 4.78 | 3.08 |
| Tanzania | 4.77 | 1.19 |
| Madagascar | 3.69 | 5.94 |
| Uganda | 3.28 | 0.68 |
| Equatorial Guinea | 1.22 | 1.21 |
| Comoros | 0.82 | 0.13 |
| Angola | 0.69 | 1.28 |
| Guinea-Bissau | 0.57 | 0.18 |
| Rwanda | 0.55 | 0.11 |
| South Sudan | 0.51 | 0.87 |
| Burundi | 0.39 | 0.15 |
| Somalia | 0.38 | 0.84 |
| Burkina Faso | 0.31 | 0.35 |
| Mozambique | 0.27 | 0.04 |

# **Appendix Table S22: Top 10 countries with suitable habitats for MPXV (ranked by population)**

| **Continent** | **Country** | **Risk area (km^2^)** | **Risk population** | **Imported risk index** |
| --- | --- | --- | --- | --- |
| Asia | Indonesia | 1 712 636 | 377 745 544 | 2641 |
| Asia | India | 153 934 | 194 915 913 | 46 748 |
| Asia | Philippines | 276 819 | 145 292 685 | 6023 |
| Americas | Brazil | 4 602 118 | 116 330 366 | 3498 |
| Asia | Vietnam | 99 232 | 72 098 242 | 1239 |
| Americas | Colombia | 999 272 | 57 707 030 | 305 |
| Asia | Thailand | 121 555 | 52 857 369 | 4459 |
| Asia | Malaysia | 239 964 | 47 536 545 | 9632 |
| Asia | Sri Lanka | 62 031 | 28 304 773 | 669 |
| Asia | Cambodia | 146 311 | 25 275 963 | 556 |

# **Appendix Table S23: Top 10 countries with suitable habitats for MPXV (ranked by area)**

| **Continent** | **Country** | **Risk area (km^2^)** | **Risk population** | **Imported risk index** |
| --- | --- | --- | --- | --- |
| Americas | Brazil | 4 602 118 | 116 330 366 | 3497 |
| Asia | Indonesia | 1 712 636 | 377 745 544 | 2640 |
| Americas | Colombia | 999 272 | 57 707 030 | 304 |
| Americas | Venezuela | 656 277 | 17 494 623 | 107 |
| Americas | Peru | 502 883 | 4 069 359 | 177 |
| Oceania | Papua New Guinea | 394 458 | 11 497 008 | 10 |
| Asia | Philippines | 276 819 | 145 292 685 | 6022 |
| Asia | Malaysia | 239 964 | 47 536 545 | 9632 |
| Americas | Guyana | 190 973 | 1 013 011 | 6 |
| Asia | India | 153 934 | 194 915 913 | 46 747 |

# **Appendix Table S24: The imported risk of MPXV caused by flight from endemic countries**

| **Imported countries** | **Exported countries** | | | | | | | | | | | | |
| --- | --- | --- | --- | --- | --- | --- | --- | --- | --- | --- | --- | --- | --- |
|  | Sum of  West African  clade | Sum of  Congo Basin  clade | Nigeria | Ghana | Cote d'Ivoire | Cameroon* | Democratic  Republic  of the Congo | Republic of  the Congo | Gabon | Sierra Leone | Benin | Liberia | Central  African  Republic |
| United Kingdom | 159 380 | 79 796 | 155 474 | 49 137 | 6167 | 3886 | 3808 | 4413 | 2197 | 11 623 | 391 | 1963 | 117 |
| France | 31 265 | 149 834 | 13 674 | 2933 | 54 682 | 34 314 | 13 483 | 22 812 | 23 542 | 2647 | 10 858 | 434 | 1720 |
| United States | 110 008 | 43 155 | 107 441 | 28 061 | 5373 | 3431 | 2447 | 1571 | 1605 | 1082 | 1136 | 851 | 165 |
| South Africa | 55 538 | 49 369 | 53 899 | 16 505 | 4399 | 2487 | 15 085 | 3394 | 6072 | 1760 | 776 | 395 | 135 |
| United Arab Emirates | 64 350 | 26 837 | 62 575 | 9629 | 3824 | 3240 | 1083 | 8331 | 1308 | 443 | 454 | 155 | 145 |
| China | 40 497 | 39 406 | 37 176 | 12 166 | 4255 | 3984 | 7228 | 5184 | 4562 | 2856 | 940 | 1329 | 223 |
| Italy | 26 720 | 32 862 | 24 642 | 10 882 | 8653 | 3707 | 1493 | 5912 | 1510 | 878 | 1529 | 225 | 153 |
| Ethiopia | 25 094 | 31 503 | 23 705 | 2326 | 4325 | 2568 | 13 196 | 8381 | 736 | 177 | 1014 | 106 | 64 |
| Senegal | 16 052 | 36 605 | 14 869 | 6793 | 17 904 | 1307 | 303 | 4277 | 3466 | 1006 | 2088 | 530 | 115 |
| India | 28 541 | 18 207 | 27 404 | 5418 | 4246 | 967 | 3182 | 1615 | 1670 | 615 | 921 | 653 | 58 |
| Germany | 19 814 | 22 787 | 17 017 | 12 302 | 2364 | 4913 | 1239 | 814 | 1142 | 909 | 1524 | 341 | 36 |
| Morocco | 4651 | 33 820 | 1557 | 1838 | 13 620 | 2075 | 2352 | 4067 | 5247 | 1871 | 810 | 2057 | 2977 |
| Saudi Arabia | 30 211 | 6688 | 29947 | 4698 | 924 | 503 | 72 | 115 | 187 | 133 | 302 | 13 | 6 |
| Kenya | 14 235 | 22 215 | 12194 | 5318 | 2085 | 1862 | 8100 | 1175 | 406 | 1655 | 1829 | 1110 | 715 |
| Belgium | 10 976 | 21 369 | 1968 | 2050 | 2641 | 3520 | 9492 | 224 | 344 | 4102 | 741 | 7247 | 15 |

Only the top 15 risk exporting countries and the top 15 risk importing countries are reserved for the number of risk population exported from infected countries to other countries in the world by flight.

*For Cameroon, we assumed that the Congo Basin clade has the same export risk as the West African clade.

# **Appendix Table S25: The imported risk of MPXV caused by flight from epidemic countries**

| **Imported countries** | Exported countries | | | | | | | | | | | | |
| --- | --- | --- | --- | --- | --- | --- | --- | --- | --- | --- | --- | --- | --- |
|  | United Kingdom | Spain | Germany | France | United States | Netherlands | Switzerland | Portugal | Canada | Belgium | Brazil | Peru | Israel |
| Italy | 217.83 | 367.08 | 138.45 | 104.34 | 20.81 | 69.58 | 30.89 | 41.44 | 8.78 | 44.07 | 1.34 | 0.44 | 0.86 |
| Ireland | 200.272 | 119.71 | 22.259 | 18.679 | 10.602 | 19.406 | 5.632 | 25.836 | 3.481 | 7.11 | 0.112 | 0.013 | 0.052 |
| Turkey | 49.868 | 23.4236 | 139.2068 | 14.9246 | 5.8403 | 22.4245 | 15.6412 | 3.6719 | 1.4509 | 5.4033 | 0.0693 | 0.0071 | 0.4404 |
| Poland | 117.7292 | 48.9736 | 28.4154 | 13.6801 | 3.2504 | 16.7056 | 6.1228 | 7.0925 | 1.7827 | 8.913 | 0.0675 | 0.0081 | 0.2421 |
| Greece | 79.926 | 18.6255 | 70.7981 | 19.973 | 2.7959 | 14.6369 | 13.513 | 2.9441 | 2.5518 | 11.6082 | 0.0692 | 0.0058 | 0.4191 |
| Austria | 28.596 | 52.501 | 65.785 | 11.187 | 4.053 | 16.653 | 15.649 | 7.5 | 1.134 | 3.979 | 0.117 | 0.022 | 0.195 |
| Denmark | 53.786 | 69.439 | 22.682 | 13.204 | 6.377 | 14.878 | 7.145 | 7.737 | 1.598 | 6.23 | 0.12 | 0.032 | 0.069 |
| Sweden | 37.966 | 72.454 | 27.131 | 11.951 | 5.971 | 12.229 | 6.959 | 6.237 | 1.098 | 3.581 | 0.109 | 0.054 | 0.047 |
| Mexico | 8.09 | 24.81 | 5.15 | 5.07 | 98.92 | 2.05 | 0.87 | 0.76 | 28.37 | 0.46 | 0.81 | 0.93 | 0 |
| Norway | 38.079 | 73.848 | 16.122 | 9.621 | 4.407 | 12.691 | 3.821 | 5.015 | 0.797 | 2.28 | 0.075 | 0.016 | 0.042 |
| China | 22.532 | 23.589 | 21.026 | 13.133 | 32.506 | 6.883 | 3.581 | 2.225 | 14.114 | 1.5 | 0.273 | 0.095 | 0 |
| Romania | 36.6489 | 47.1175 | 23.0805 | 8.3073 | 0.9 | 6.7213 | 3.4961 | 2.8965 | 1.1442 | 7.7232 | 0.0315 | 0.0035 | 0.5088 |
| Russian | 11.851 | 46.454 | 33.794 | 12.63 | 4.205 | 7.747 | 6.528 | 3.936 | 1.025 | 2.848 | 0.072 | 0.023 | 0.739 |
| Morocco | 15.2096 | 32.4925 | 8.0388 | 43.3768 | 0.577 | 8.2353 | 3.3379 | 2.3568 | 1.4079 | 12.2773 | 0.0301 | 0.0013 | 0 |
| Hungary | 32.8038 | 23.1079 | 15.9233 | 6.5021 | 1.5278 | 11.1069 | 4.4629 | 3.4989 | 1.2561 | 5.9403 | 0.0425 | 0.0042 | 0.2502 |

Only the top 15 risk exporting countries and the top 15 risk importing countries are reserved for the number of risk population exported from infected countries to other countries in the world by flight.

# Appendix Table S26: Country or region abbreviations used throughout the article

| **ID** | **Country or region** | **Abbreviation** |  | **ID** | **Country or region** | **Abbreviation** |
| --- | --- | --- | --- | --- | --- | --- |
| 1 | Australia | AUS |  | 33 | Morocco | MAR |
| 2 | Austria | AUT |  | 34 | Mozambique | MOZ |
| 3 | Belgium | BEL |  | 35 | Netherlands | NLD |
| 4 | Benin | BEN |  | 36 | Nigeria | NGA |
| 5 | Brazil | BRA |  | 37 | Norway | NOR |
| 6 | Burkina Faso | BFA |  | 38 | Poland | POL |
| 7 | Cameroon | CMR |  | 39 | Portugal | PRT |
| 8 | Canada | CAN |  | 40 | Romania | ROU |
| 9 | Central African Republic | CAF |  | 41 | Russian | RUS |
| 10 | China | CHN |  | 42 | Rwanda | RWA |
| 11 | Congo | COG |  | 43 | Saudi Arabia | SAU |
| 12 | Côte d'Ivoire | CIV |  | 44 | Senegal | SEN |
| 13 | Democratic Republic of the Congo | DRC |  | 45 | Sierra Leone | SLE |
| 14 | Denmark | DNK |  | 46 | Singapore | SGP |
| 15 | Equatorial Guinea | GNQ |  | 47 | Slovenia | SVN |
| 16 | Ethiopia | ETH |  | 48 | Somalia | SOM |
| 17 | France | FRA |  | 49 | South Africa | ZAF |
| 18 | Gabon | GAB |  | 50 | South Sudan | SDS |
| 19 | Germany | DEU |  | 51 | Spain | ESP |
| 20 | Ghana | GHA |  | 52 | Spain | ESP |
| 21 | Greece | GRC |  | 53 | Sudan | SDN |
| 22 | Guinea | GIN |  | 54 | Sweden | SWE |
| 23 | Guinea Bissau | GNB |  | 55 | Switzerland | CHE |
| 24 | Hungary | HUN |  | 56 | Switzerland | CHE |
| 25 | India | IND |  | 57 | Tanzania | TZA |
| 26 | Ireland | IRL |  | 58 | Togo | TGO |
| 27 | Israel | ISR |  | 59 | Turkey | TUR |
| 28 | Italy | ITA |  | 60 | Uganda | UGA |
| 29 | Kenya | KEN |  | 61 | United Arab Emirates | ARE |
| 30 | Liberia | LBR |  | 62 | United Kingdom | GBR |
| 31 | Madagascar | MDG |  | 63 | United States of America | USA |
| 32 | Mexico | MEX |  |  |  |  |

Appendix Figure S1: Timeline of MPXV cases from 1970 to 2022. (A) the annual and cumulative cases of monkeypox by continent, (B) the seasonality of MPXV case numbers.


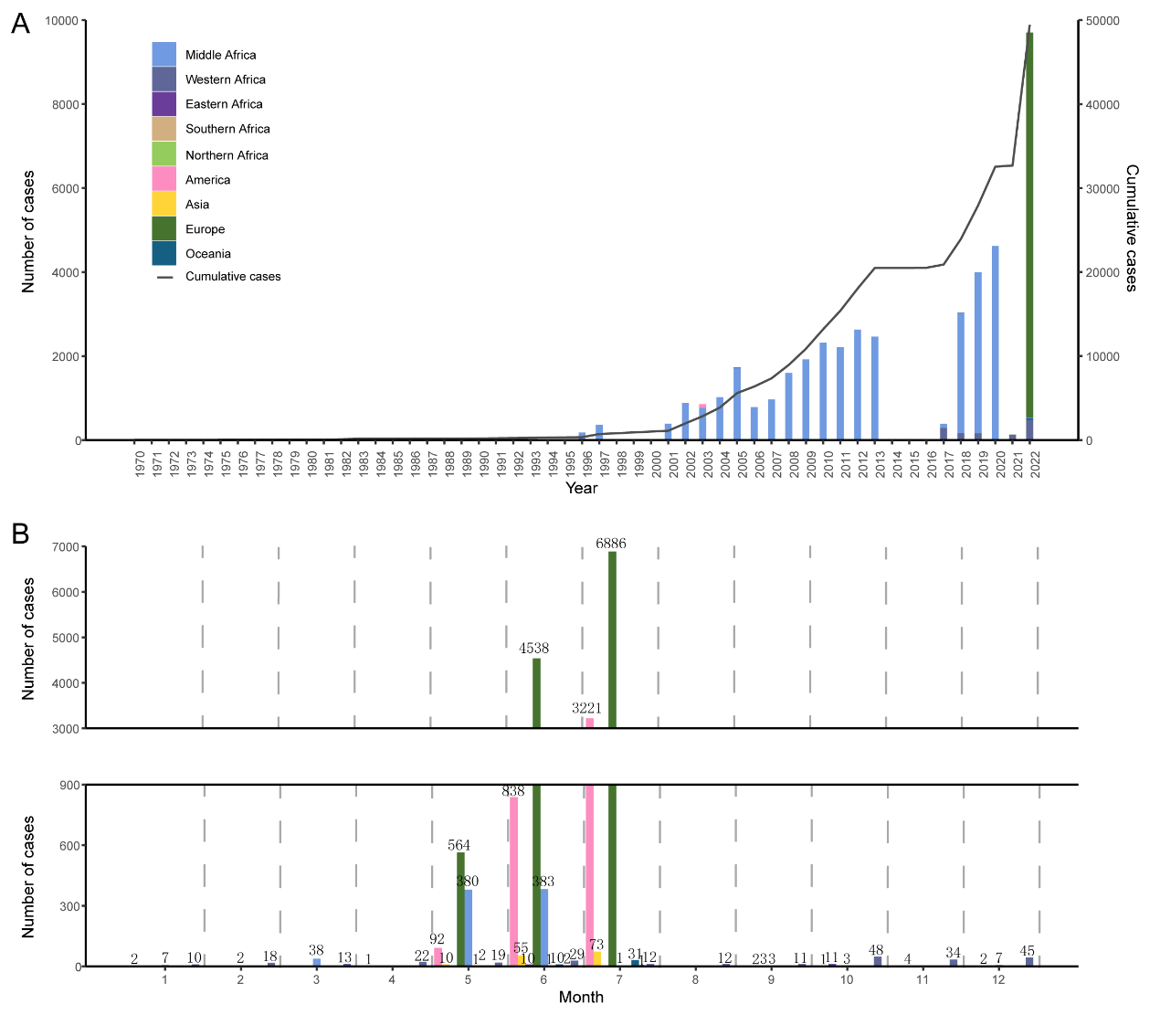


Appendix Figure S2: The number of MPXV human infections by periods and countries. (A) Illustrates the number of MPXV human infections through time, with the area of each circle and its position along the y-axis representing the number of cases. The onset year is represented by the color as per. (B–G) Show these numbers of human infections over a series of time periods. Panel H show all infections through time. In (B–H) the clades of MPXV is illustrated by the symbol shape, the number of cases by size and reported years by color. Endemic areas have been reported are colored in blue. The country abbreviations were listed with full name in Table S24.


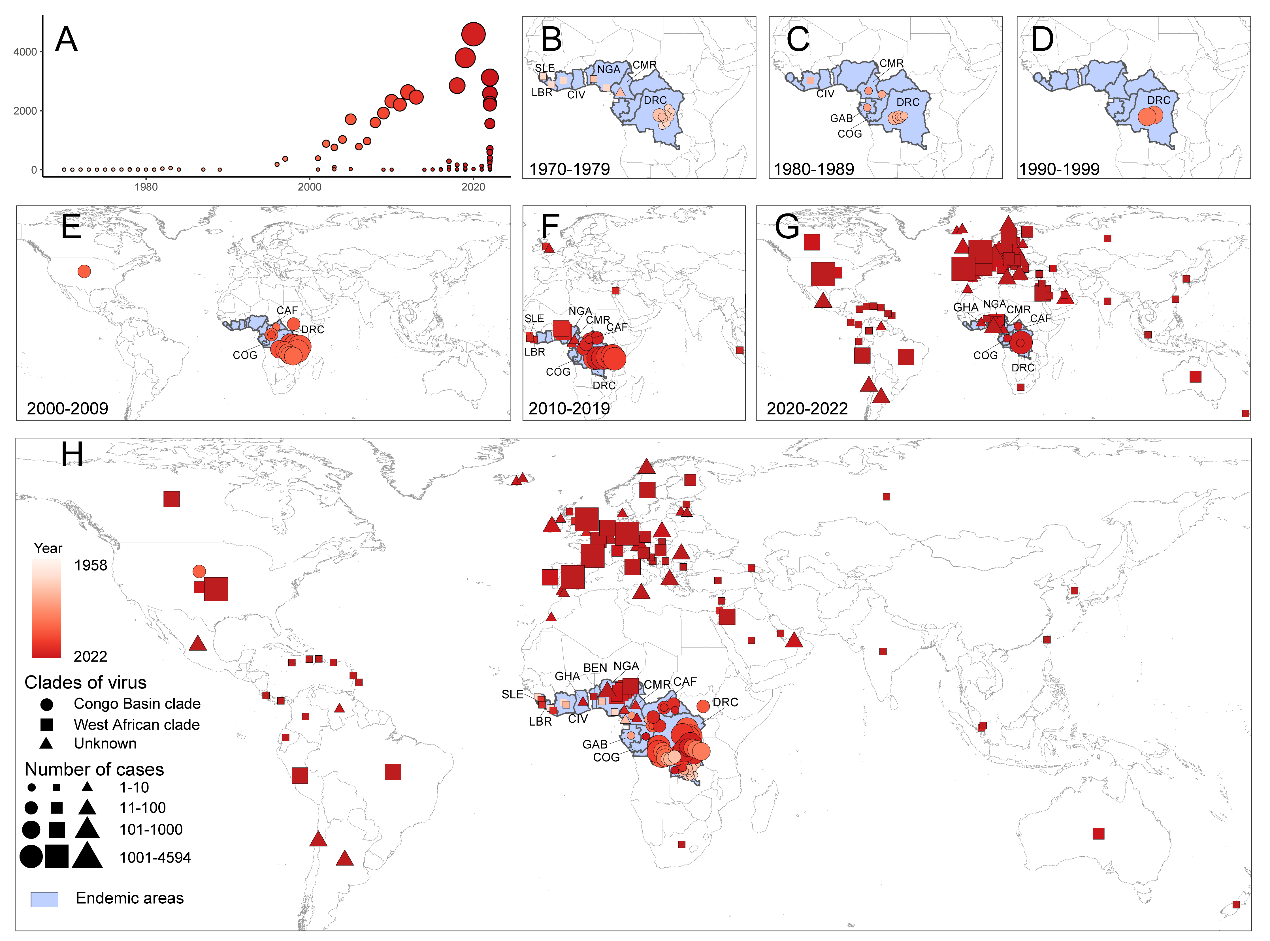


Appendix Figure S3: The locations of reported MPXV infections in animals and zoonotic transmission to human. (A) Shows the locations of reported MPXV virus infection in animals and zoonotic transmission to human. (B–D) Show these records over three different time periods. Numbers refer to records as listed in Appendix Table S11and S12. In all panels, the species in which infection was detected is given by symbol shape and the year recorded by symbol color. Endemic areas which has been reported are colored in blue.


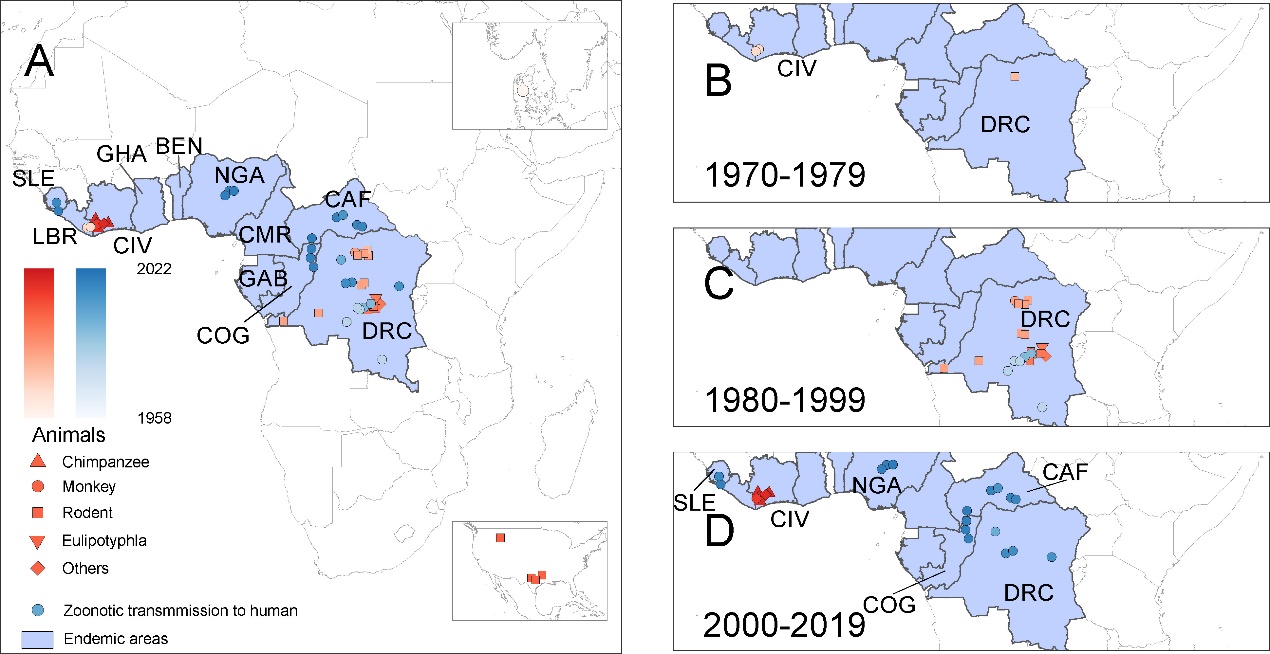


Appendix Figure S4: The effective reproduction number (Rt) of monkeypox in human-to-human transmission. (A) The effective reproduction number (Rt) with 95% CI from 1990 to 2020 between West African clade and Congo Basin clade; (B) The effective reproduction number (Rt) in main epidemic countries from April 21 to July 22, 2022.


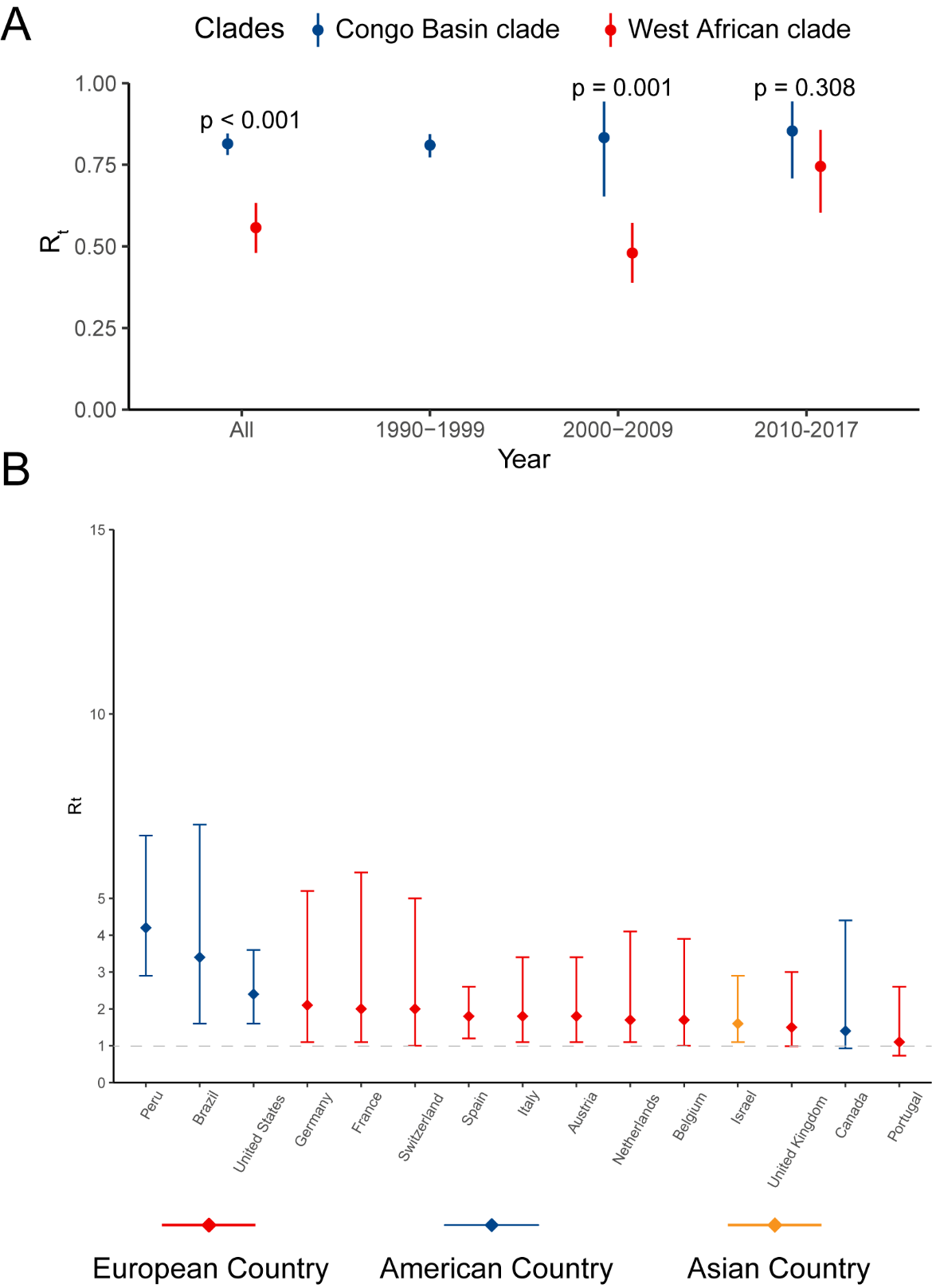


Appendix Figure S5: Correlation matrix of variables for *Cricetomys gambianus*. Heatmap representing the correlation between features using Spearman correlation coefficient. Red and blue colors in the plot represent the positive and negative monotonic relationship respectively. The absolute value of the correlation coefficient was represented by the size of the circle, and the exact value shown in the lower triangle. The variables marked red represent those excluded.


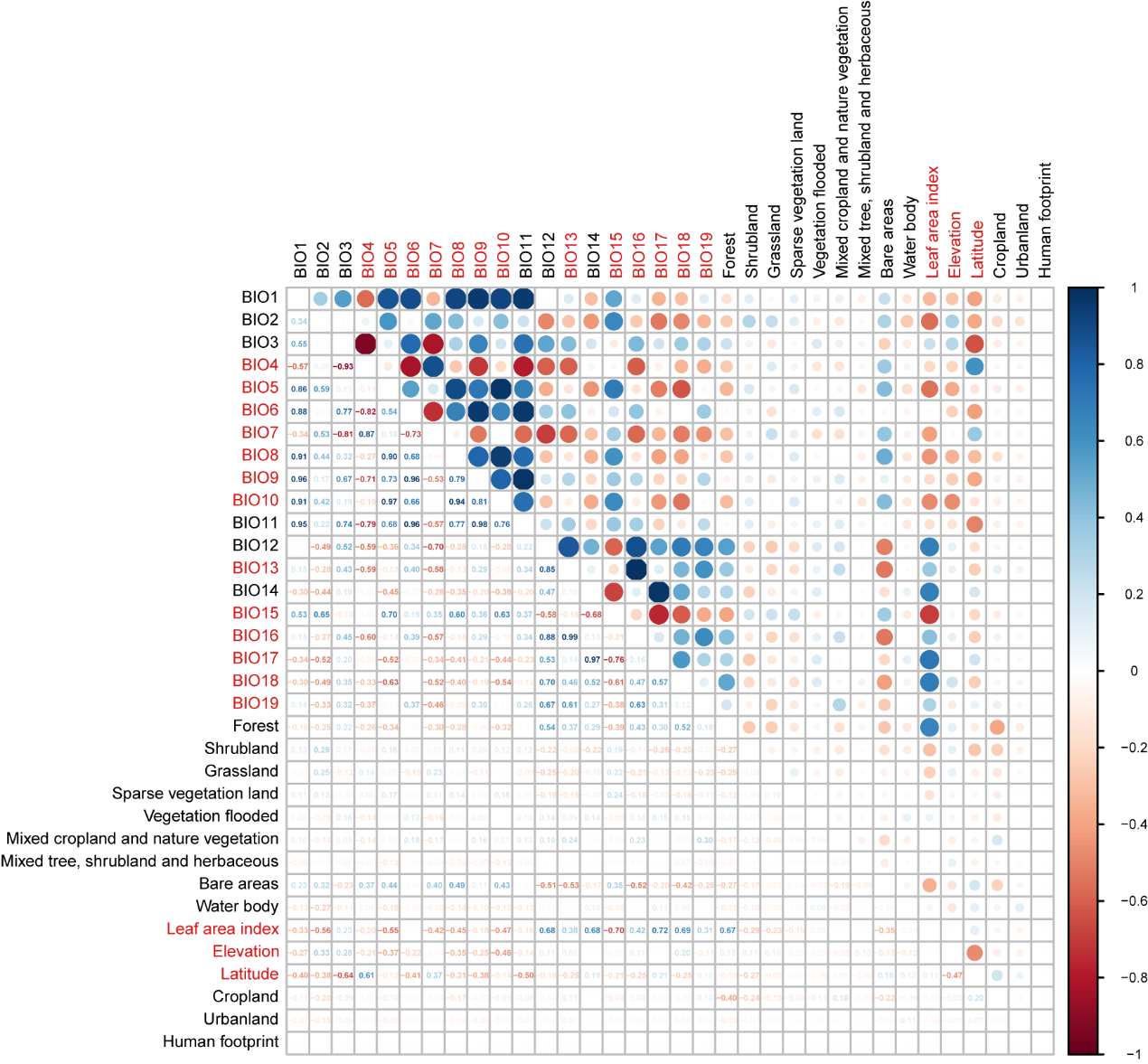


Appendix Figure S6: Correlation matrix of variables for *Funisciurus* spp. Heatmap representing the correlation between features using Spearman correlation coefficient. Red and blue colors in the plot represent the positive and negative monotonic relationship respectively. The absolute value of the correlation coefficient was represented by the size of the circle, and the exact value shown in the lower triangle. The variables marked red represent those excluded.


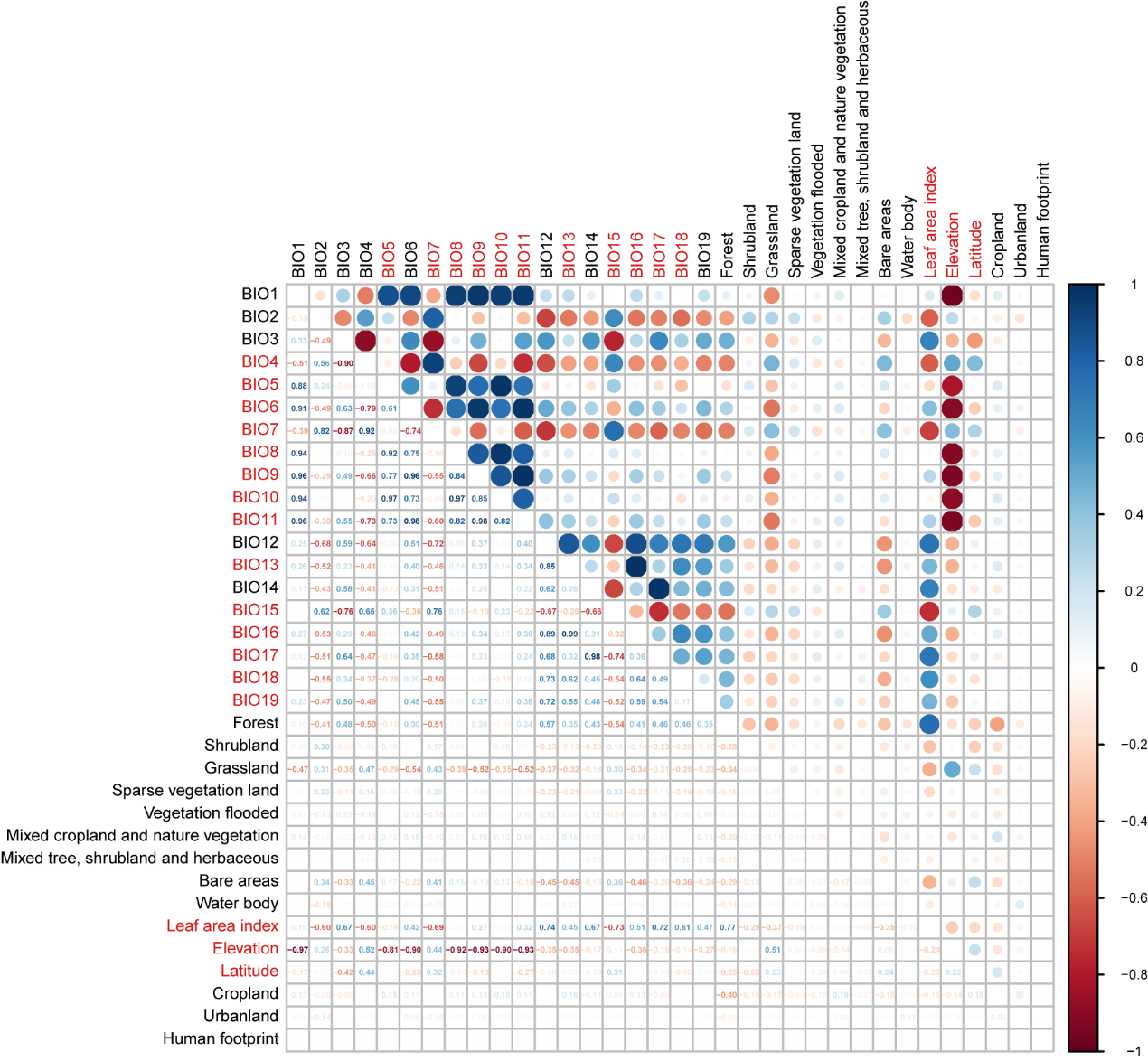


Appendix Figure S7: Correlation matrix of variables for *Graphiurus crassicaudatus*. Heatmap representing the correlation between features using Spearman correlation coefficient. Red and blue colors in the plot represent the positive and negative monotonic relationship respectively. The absolute value of the correlation coefficient was represented by the size of the circle, and the exact value shown in the lower triangle. The variables marked red represent those excluded.


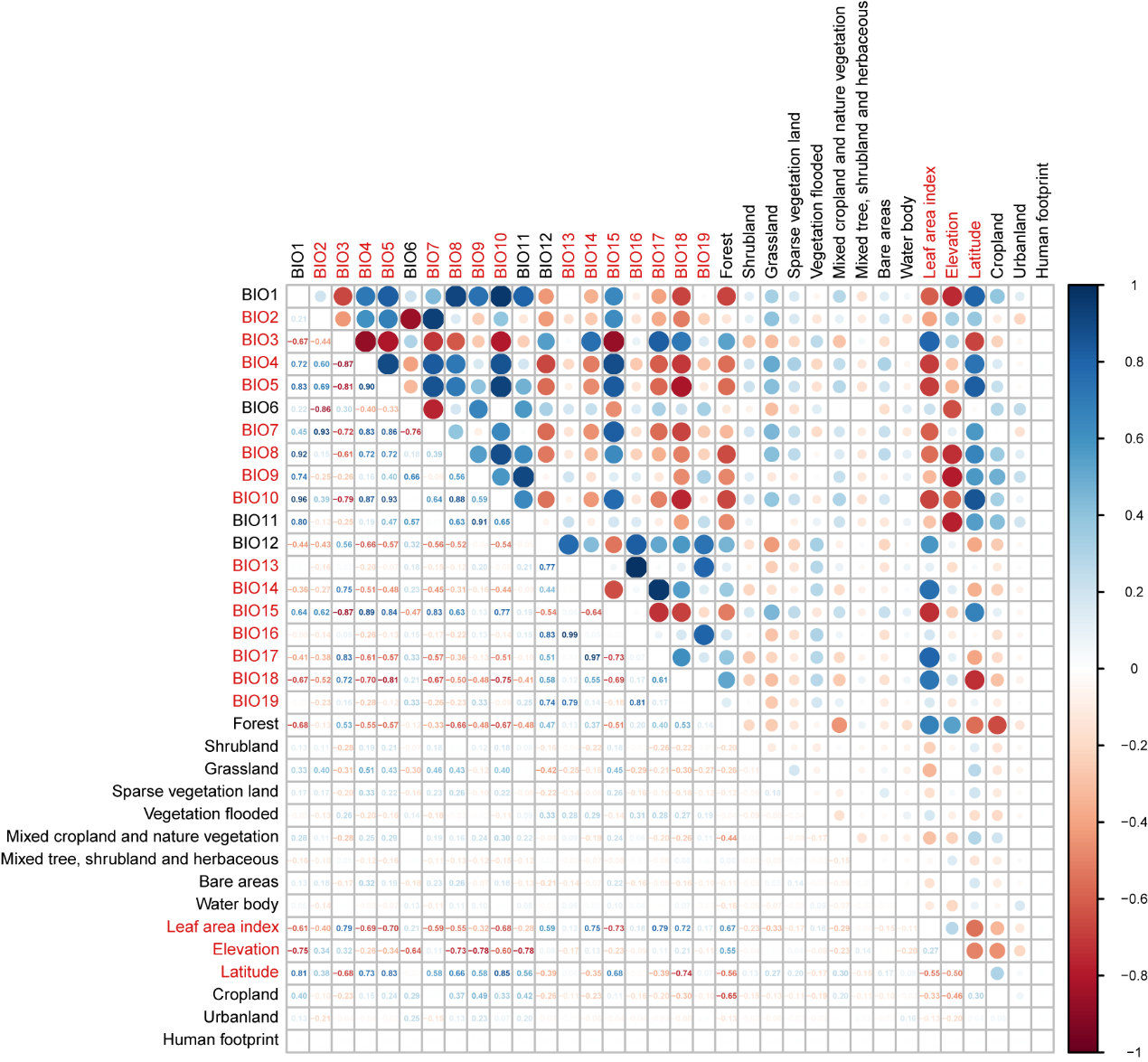


Appendix Figure S8: Correlation matrix of variables for *Graphiurus lorraineus*. Heatmap representing the correlation between features using Spearman correlation coefficient. Red and blue colors in the plot represent the positive and negative monotonic relationship respectively. The absolute value of the correlation coefficient was represented by the size of the circle, and the exact value shown in the lower triangle. The variables marked red represent those excluded.


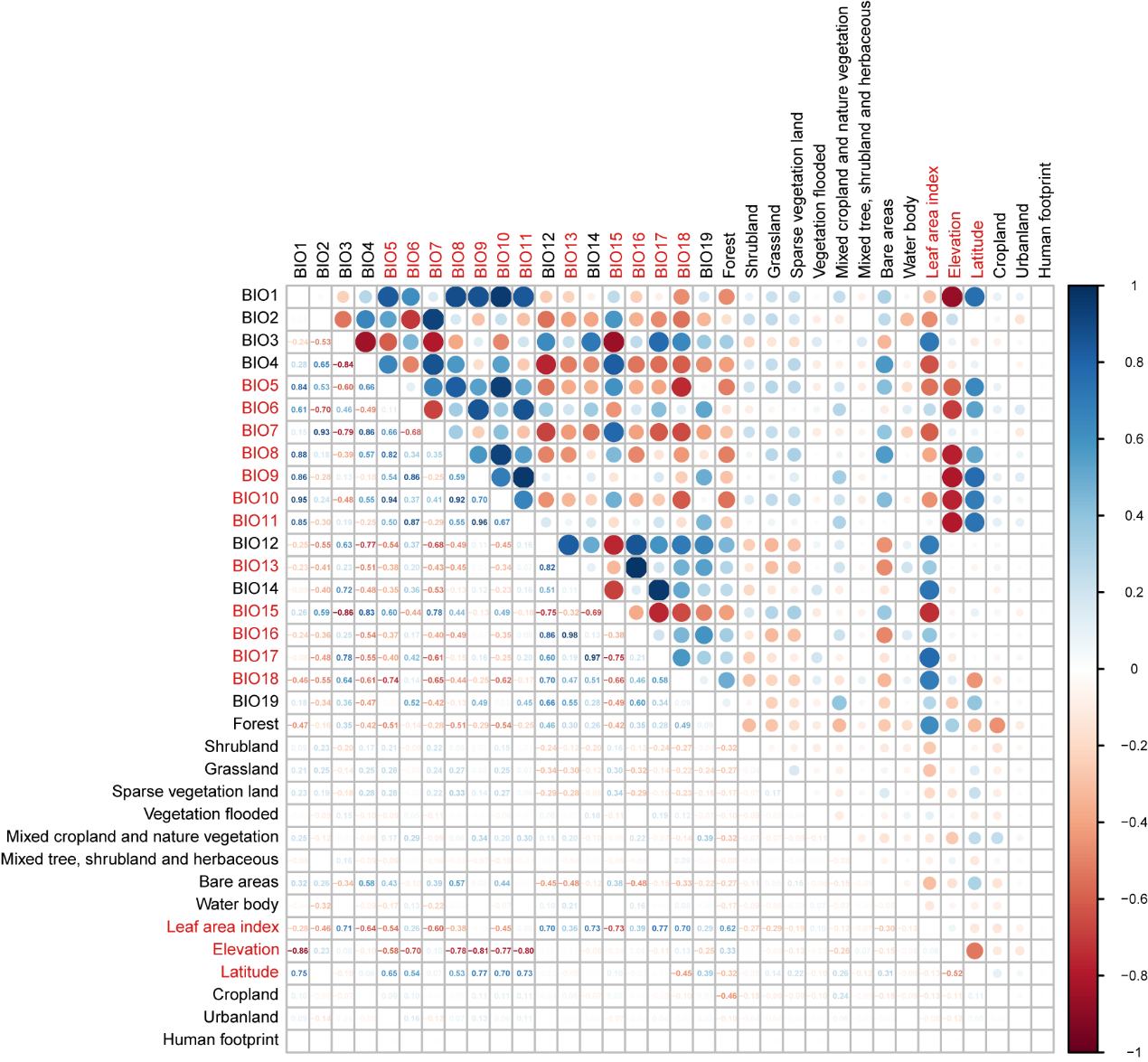


Appendix Figure S9: Correlation matrix of variables for MPXV. Heatmap representing the correlation between features using Spearman correlation coefficient. Red and blue colors in the plot represent the positive and negative monotonic relationship respectively. The absolute value of the correlation coefficient was represented by the size of the circle, and the exact value shown in the lower triangle. The variables marked red represent those excluded.


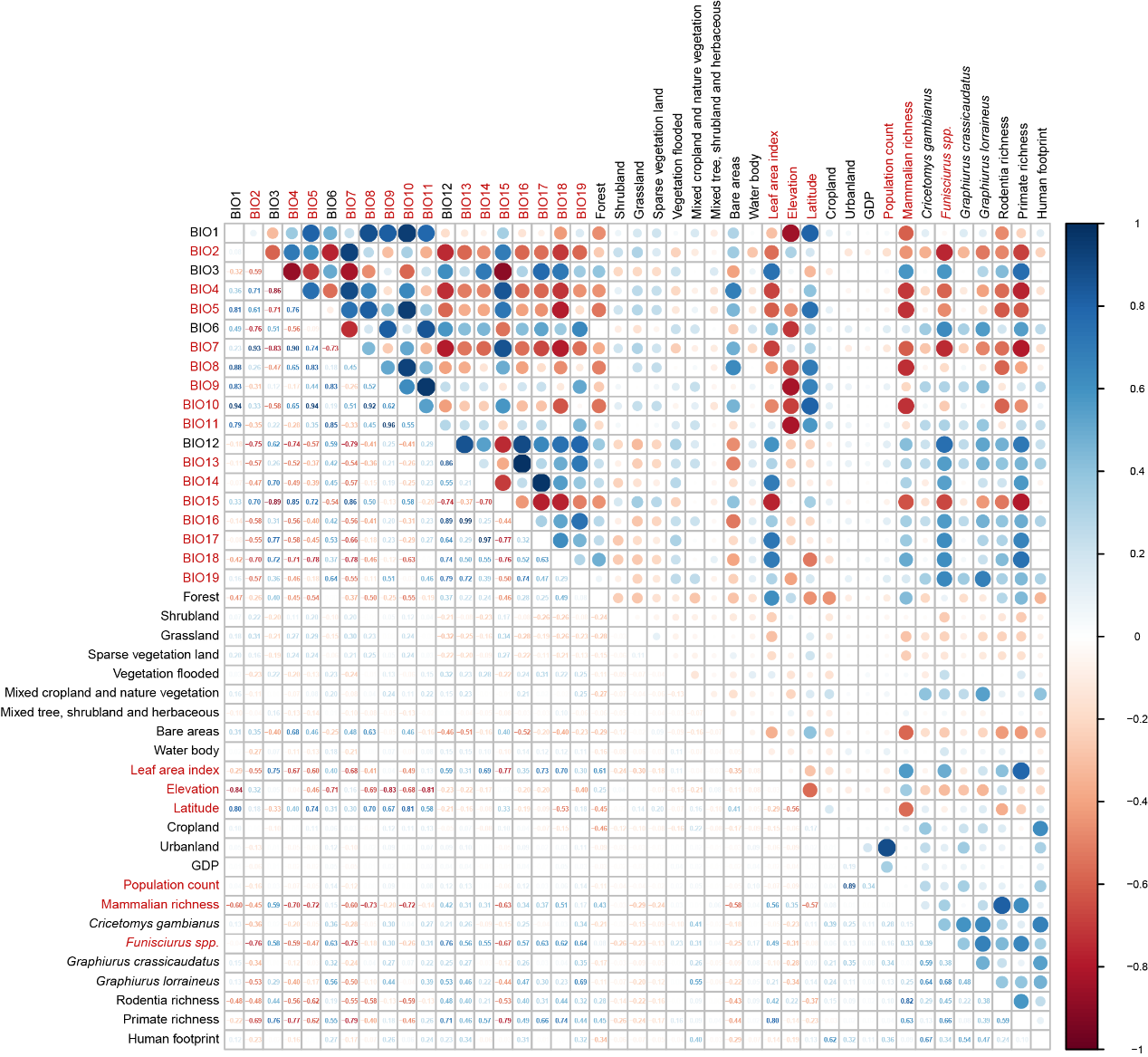


Appendix Figure S10: The relative contribution and response curve of BRT models for *Cricetomys gambianus*. (A) The relative influence of predictors on occurrence probability. (B) Effect plots of predictors based on BRT models ordered by mean relative contributions, which was present in parentheses. The mean curves (red) and 95% percentiles (red) show the influence on predicting the probability of occurrence. The histograms (grey) show the frequency distributions of the predictors.


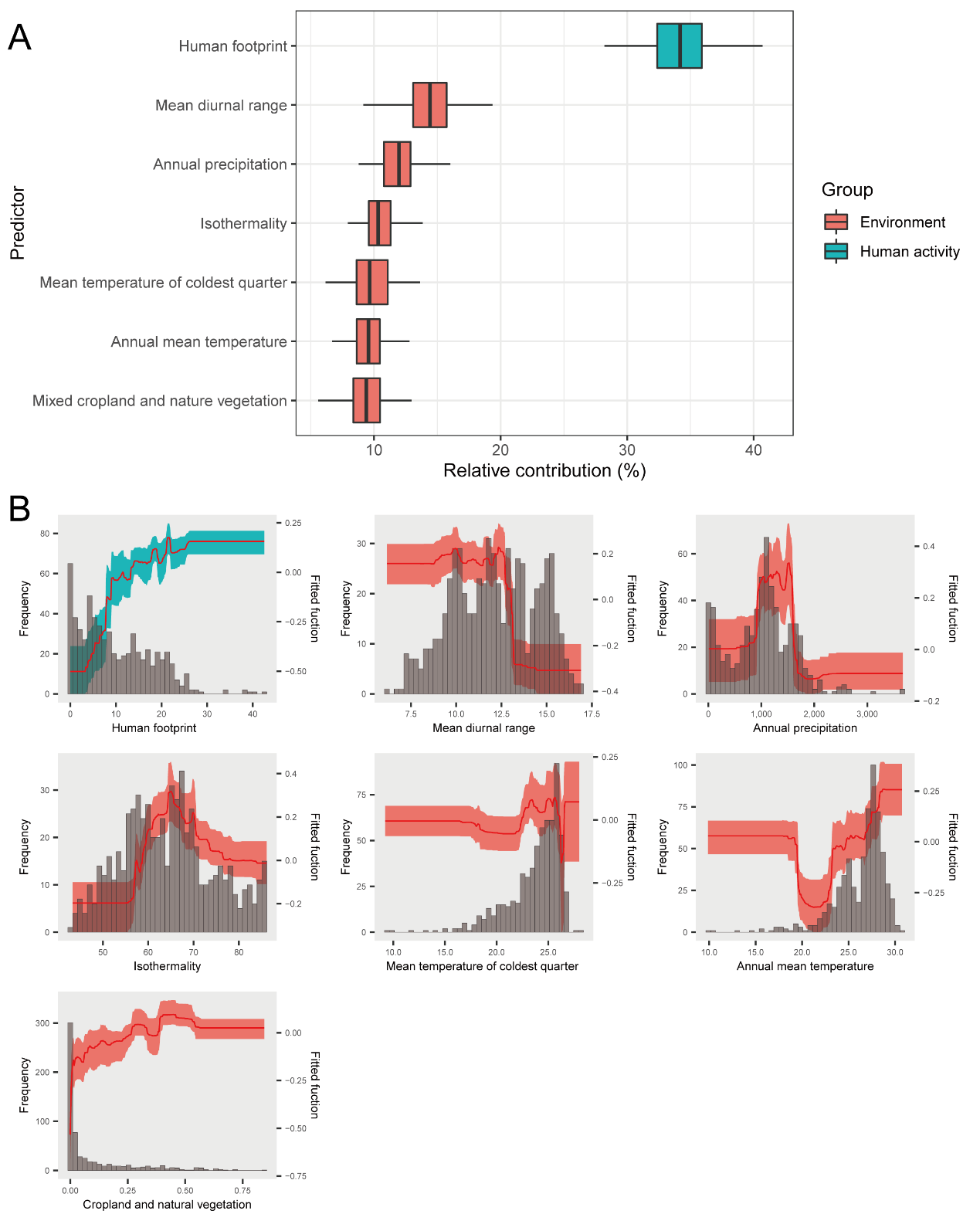


Appendix Figure S11: The relative contribution and response curve of BRT models for *Funisciurus* spp. (A) The relative influence of predictors on occurrence probability. (B) Effect plots of predictors based on BRT models ordered by mean relative contributions, which was present in parentheses. The mean curves (red) and 95% percentiles (red) show the influence on predicting the probability of occurrence. The histograms (grey) show the frequency distributions of the predictors.


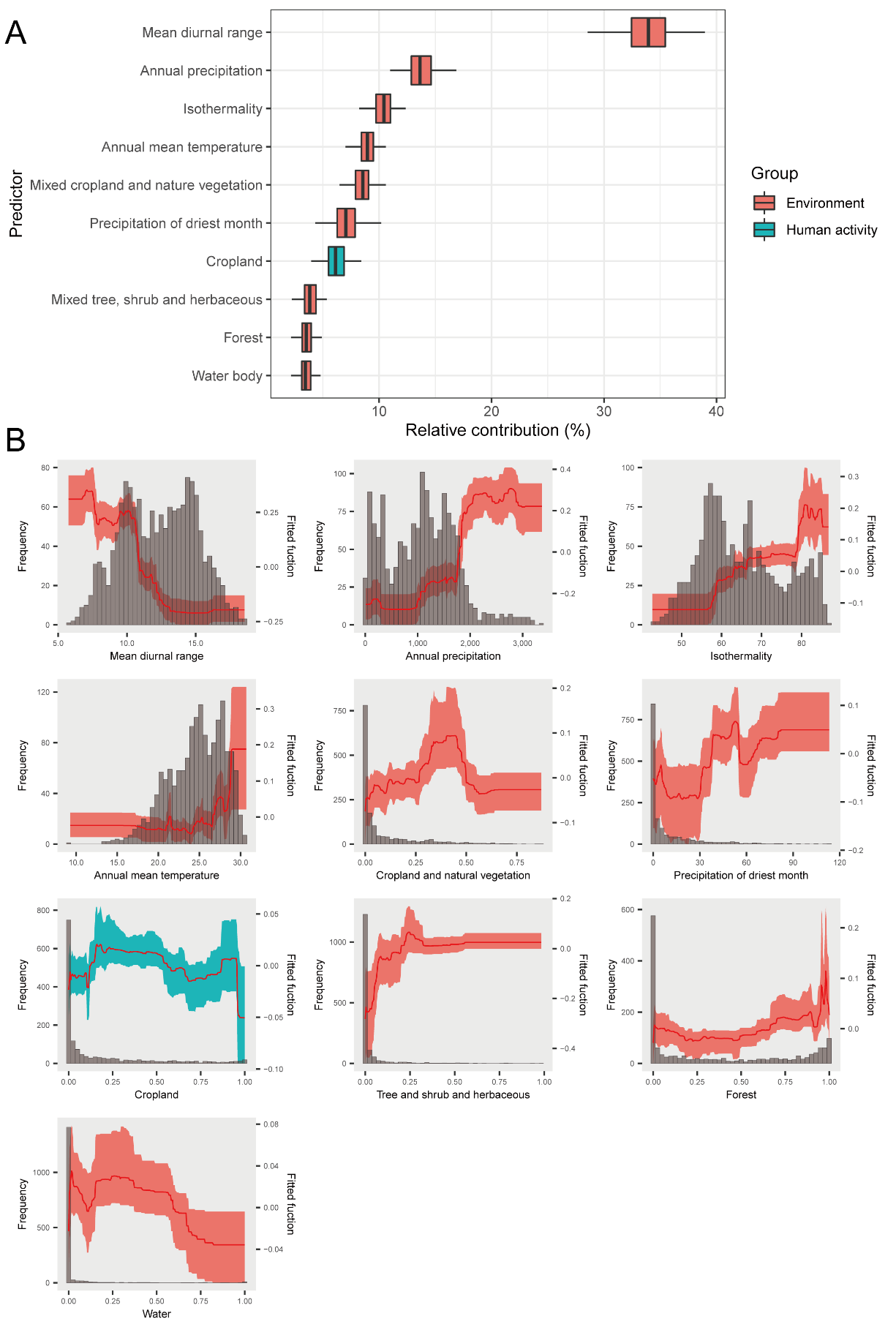


Appendix Figure S12: The relative contribution and response curve of BRT models for *Graphiurus crassicaudatus*. (A) The relative influence of predictors on occurrence probability. (B) Effect plots of predictors based on BRT models ordered by mean relative contributions, which was present in parentheses. The mean curves (red) and 95% percentiles (red) show the influence on predicting the probability of occurrence. The histograms (grey) show the frequency distributions of the predictors.


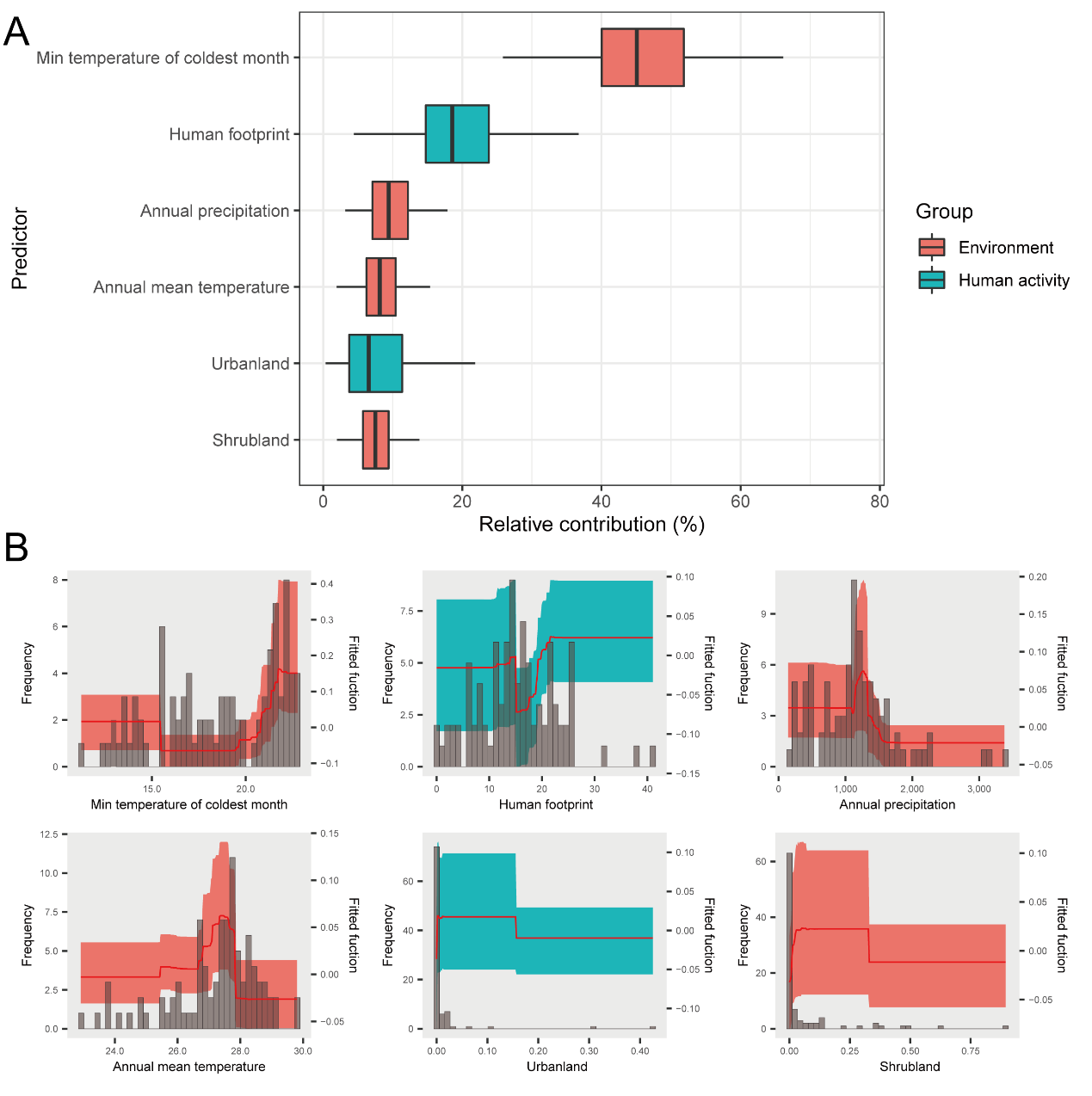


Appendix Figure S13: The relative contribution and response curve of BRT models for *Graphiurus lorraineus*. (A) The relative influence of predictors on occurrence probability. (B) Effect plots of predictors based on BRT models ordered by mean relative contributions, which was present in parentheses. The mean curves (red) and 95% percentiles (red) show the influence on predicting the probability of occurrence. The histograms (grey) show the frequency distributions of the predictors.


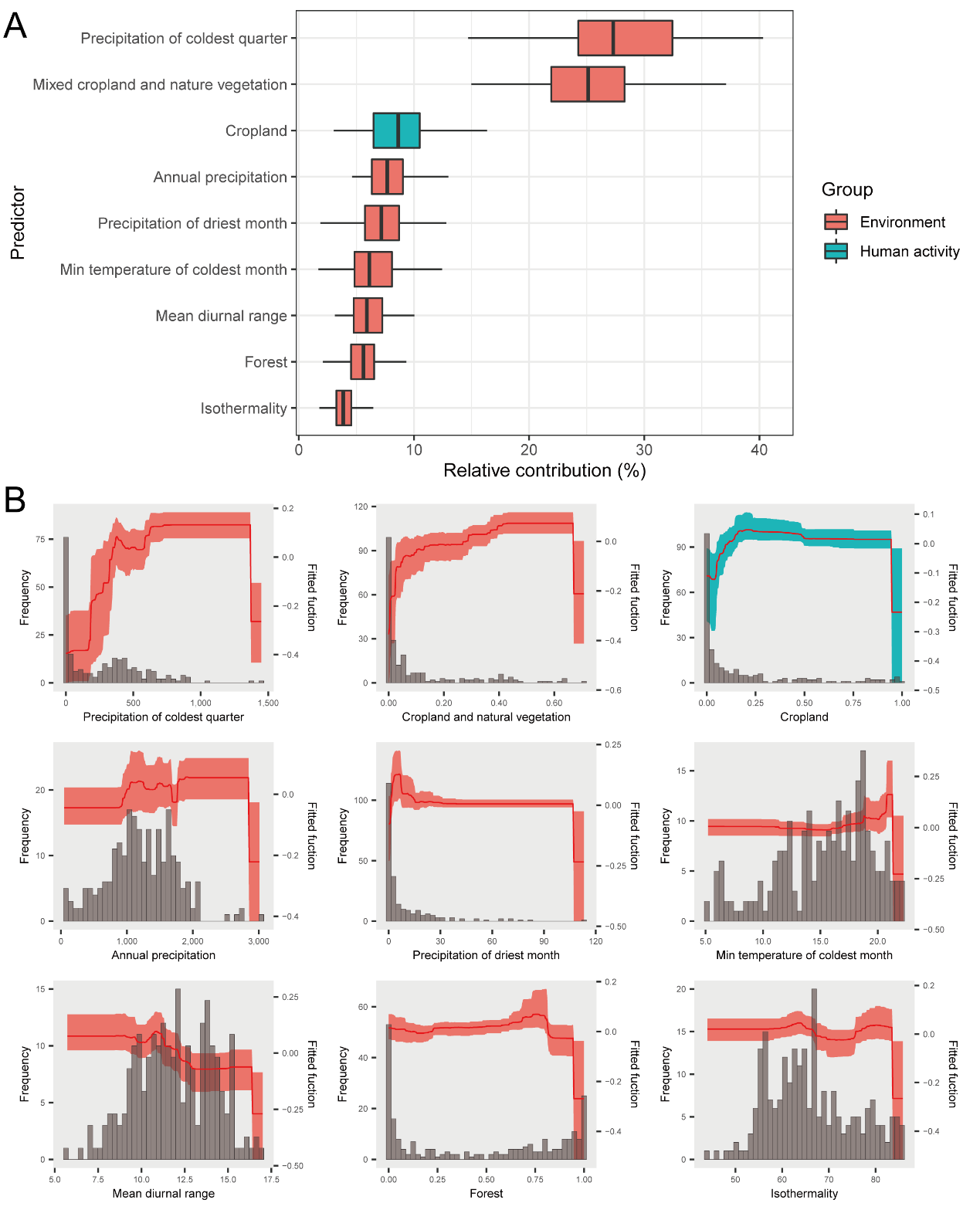


Appendix Figure S14: The relative contribution and response curve of BRT models for MPXV**.** (A) The relative influence of predictors on occurrence probability. (B) Effect plots of predictors based on BRT models ordered by mean relative contributions, which was present in parentheses. The mean curves (red) and 95% percentiles (red) show the influence on predicting the probability of occurrence. The histograms (grey) show the frequency distributions of the predictors. The partial dependence plots showed the relationships between these explanatory factors and MPXV zoonotic niche (Table S18). Two animal-related predictors (probability of *Graphiurus lorraineus*, and *Graphiurus crassicaudatus*) showed a positive correlation with zoonotic transmission of MPXV. Annual precipitation, min temperature of coldest month and isothermality were generally shown a positive correlation with the occurrence of with MPXV, and annual mean temperature was shown a quadratic pattern, in where low or high annual precipitation decreased the environmental suitability for MPXV. A negative correlation was shown between the Rodentia richness and zoonotic MPXV occurrence.


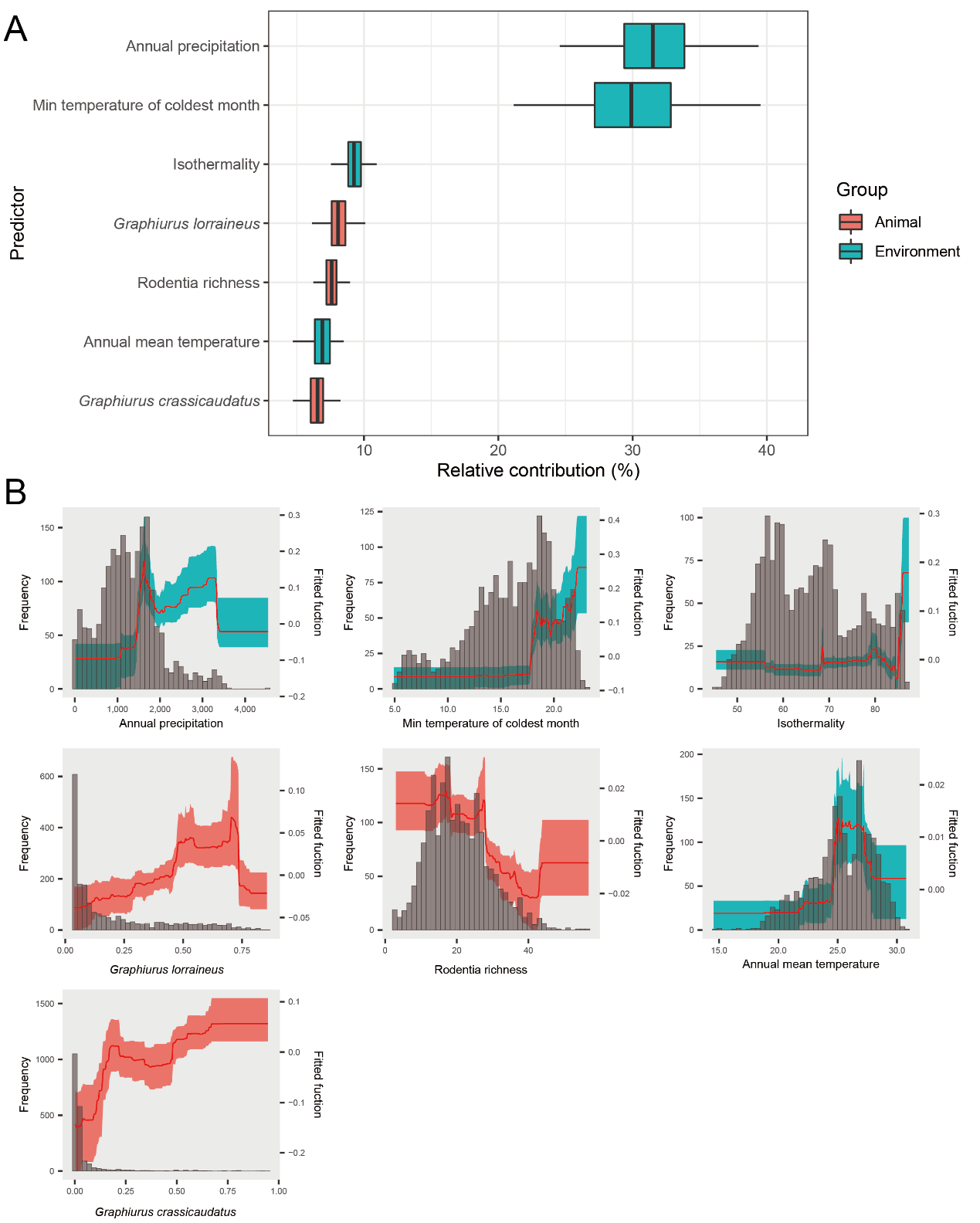


Appendix Figure S15: Global environmental suitability of MPXV**.** The environmental suitability was colored from blue to red by ascending order. In addition to Africa, the BRT model predicted that the northern part of South America, Caribbean States, and many areas in Southeast and South Asia as well as Western Pacific Region have highly suitable environment for the MPXV occurrence once the virus invades.


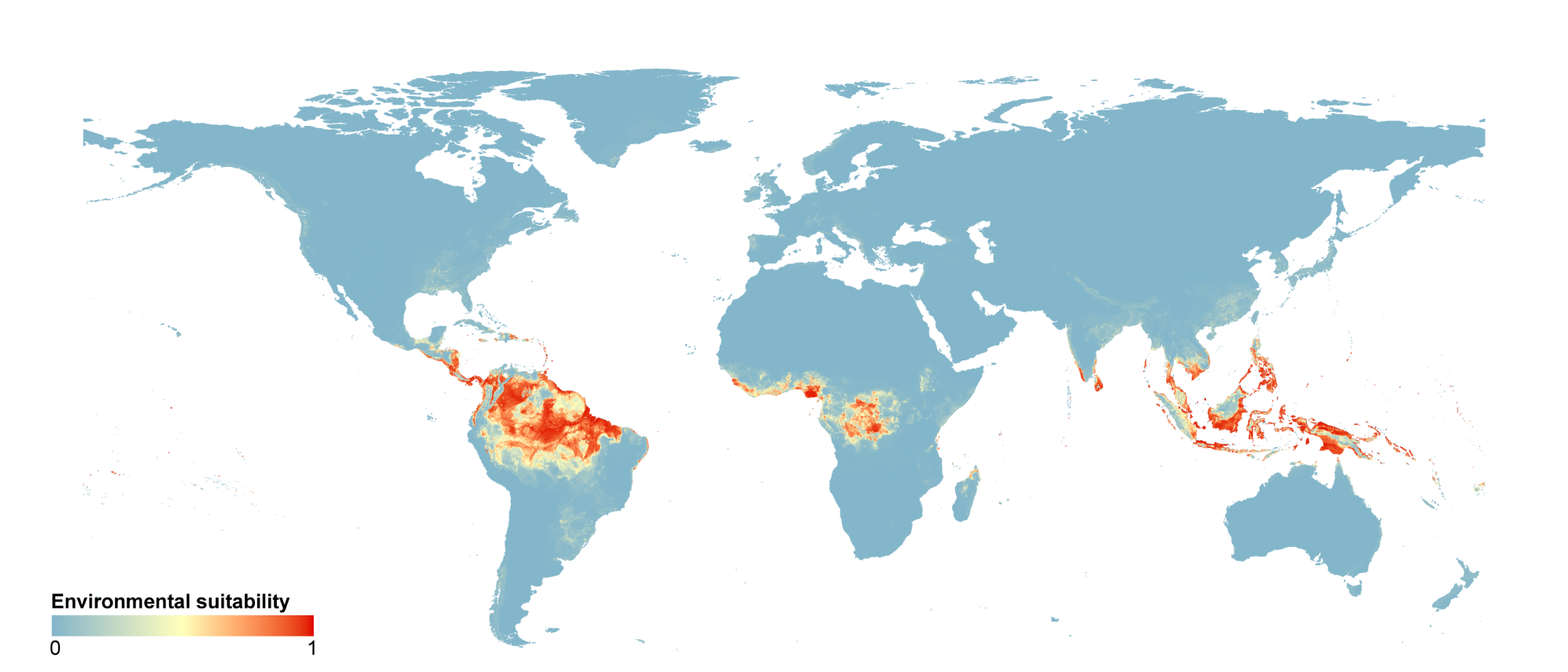


Appendix Figure S16: The chord graph of interregional risk of MPXV from endemic countries (A) and main current epidemic countries (B) caused by flight**.** (A): The corresponding links between the top 15 countries at potentially imported risk and endemic countries are shown, with the information about the proportion of risk source and probable MPXV clades. (B): The corresponding links between the top 15 countries at potentially imported risk and main current epidemic countries are shown. The country abbreviations were listed with full name in Table S24.


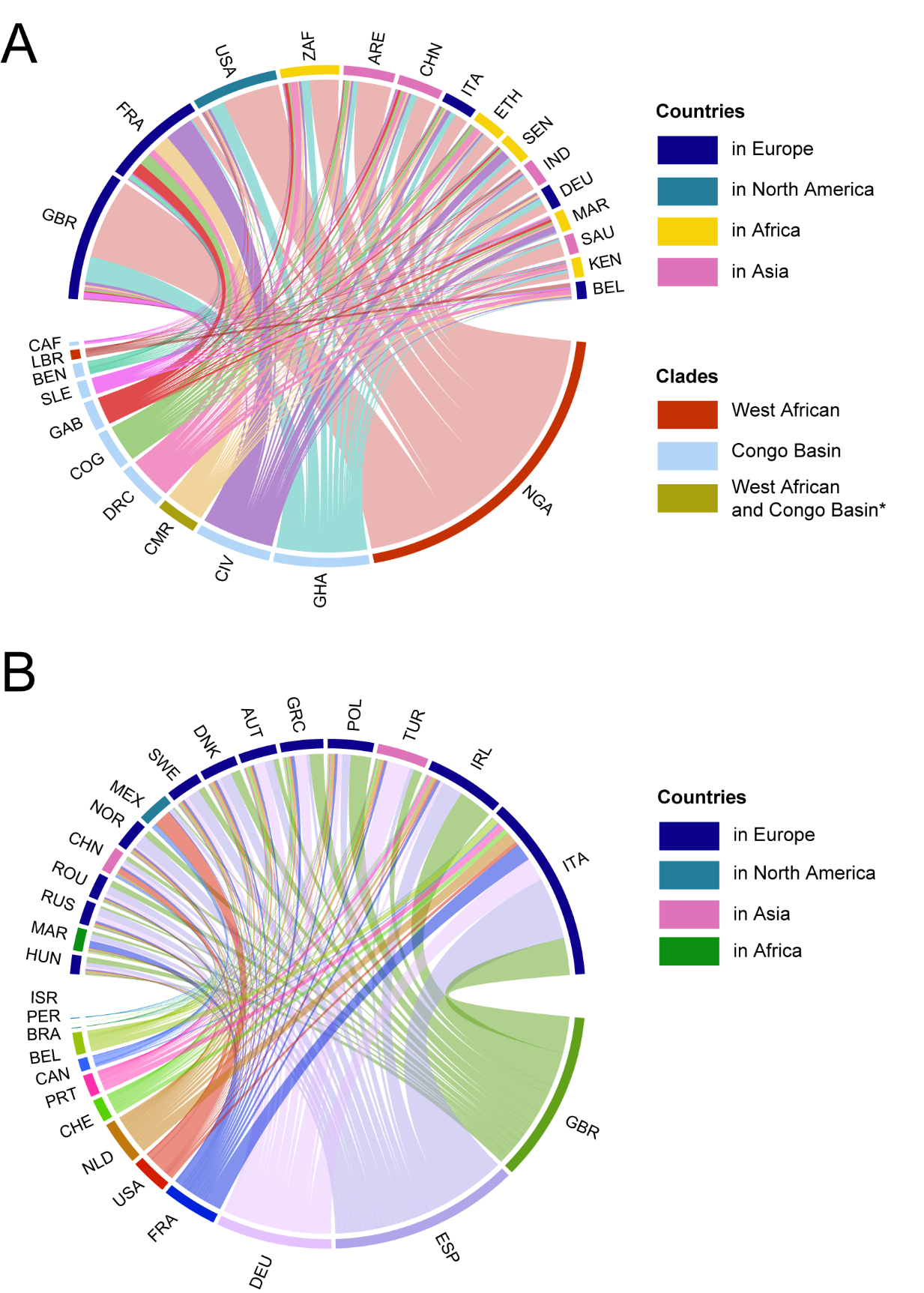


# Appendix Text S6: Reference

1. Eltvedt AK, Christiansen M, Poulsen A. A case report of Monkeypox in a 4-year-old boy from the DR Congo: challenges of diagnosis and management. *Case Rep Pediatr*. 2020; 2020: 8572596.

2. Nakoune E, Lampaert E, Ndjapou SG, Janssens C, Zuniga I, Van Herp M, et al. A nosocomial outbreak of human Monkeypox in the Central African Republic. *Open Forum Infect Dis*. 2017; 4(4): ofx168.

3. Durski KN, McCollum AM, Nakazawa Y, Petersen BW, Reynolds MG, Briand S, et al. Emergence of Monkeypox - West and Central Africa, 1970-2017. *MMWR Morb Mortal Wkly Rep*. 2018; 67(10): 306-10.

4. Nolen LD, Osadebe L, Katomba J, Likofata J, Mukadi D, Monroe B, et al. Extended human-to-human transmission during a Monkeypox outbreak in the Democratic Republic of the Congo. *Emerg Infect Dis*. 2016; 22(6): 1014-21.

5. Guagliardo SAJ, Doshi RH, Reynolds MG, Dzabatou-Babeaux A, Ndakala N, Moses C, et al. Do monkeypox exposures vary by ethnicity? comparison of Aka and Bantu suspected Monkeypox cases. *Am J Trop Med Hyg*. 2020; 102(1): 202-5.

6. Vaughan A, Aarons E, Astbury J, Balasegaram S, Beadsworth M, Beck CR, et al. Two cases of monkeypox imported to the United Kingdom, September 2018. *Euro surveillance*. 2018;23(38).

7. Kile JC, Fleischauer AT, Beard B, Kuehnert MJ, Kanwal RS, Pontones P, et al. Transmission of monkeypox among persons exposed to infected prairie dogs in Indiana in 2003. *Arch Pediatr Adolesc Med*. 2005; 159(11): 1022-5.

8. Fine PE, Jezek Z, Grab B, Dixon H. The transmission potential of monkeypox virus in human populations. *Int J Epidemiol*. 1988; 17(3): 643-50.

9. Doshi RH, Guagliardo SAJ, Dzabatou-Babeaux A, Likouayoulou C, Ndakala N, Moses C, et al. Strengthening of surveillance during Monkeypox outbreak, Republic of the Congo, 2017. *Emerg Infect Dis*. 2018;24(6):1158-60.

10. Erez N, Achdout H, Milrot E, Schwartz Y, Wiener-Well Y, Paran N, et al. Diagnosis of imported Monkeypox, Israel, 2018. *Emerg Infect Dis*. 2019; 25(5): 980-3.

11. Kalthan E, Dondo-Fongbia JP, Yambele S, Dieu-Creer LR, Zepio R, Pamatika CM. [Twelve cases of monkeypox virus outbreak in Bangassou District (Central African Republic) in December 2015]. *Bull Soc Pathol Exot* (1990). 2016;109(5): 358-63.

12. Anderson MG, Frenkel LD, Homann S, Guffey J. A case of severe monkeypox virus disease in an American child: emerging infections and changing professional values. *Pediatr Infect Dis J*. 2003; 22(12): 1093-6; discussion 6-8.

13. Jezek Z, Arita I, Mutombo M, Dunn C, Nakano JH, Szczeniowski M. Four generations of probable person-to-person transmission of human monkeypox. *Am J Epidemiol*. 1986; 123(6): 1004-12.

14. Reynolds MG, Emerson GL, Pukuta E, Karhemere S, Muyembe JJ, Bikindou A, et al. Detection of human monkeypox in the Republic of the Congo following intensive community education. *Am J Trop Med Hyg*. 2013; 88(5): 982-5.

15. Ogoina D, Izibewule JH, Ogunleye A, Ederiane E, Anebonam U, Neni A, et al. The 2017 human monkeypox outbreak in Nigeria-Report of outbreak experience and response in the Niger Delta University Teaching Hospital, Bayelsa State, Nigeria. *PloS One*. 2019; 14(4): e0214229.

16. Hughes CM, Liu L, Davidson WB, Radford KW, Wilkins K, Monroe B, et al. A tale of two viruses: coinfections of Monkeypox and Varicella Zoster Virus in the Democratic Republic of Congo. *Am J Trop Med Hyg*. 2020; 104(2): 604-11.

17. Hoff NA, Morier DS, Kisalu NK, Johnston SC, Doshi RH, Hensley LE, et al. Varicella coinfection in patients with active Monkeypox in the Democratic Republic of the Congo. *EcoHealth*. 2017; 14(3): 564-74.

18. Mbala PK, Huggins JW, Riu-Rovira T, Ahuka SM, Mulembakani P, Rimoin AW, et al. Maternal and fetal outcomes among pregnant women with human Monkeypox infection in the Democratic Republic of Congo. *J Infect Dis*. 2017;216(7):824-8.

19. Yinka-Ogunleye A, Aruna O, Ogoina D, Aworabhi N, Eteng W, Badaru S, et al. Reemergence of human monkeypox in Nigeria, 2017. *Emerg Infect Dis*. 2018; 24(6): 1149-51.

20. Meyer H, Perrichot M, Stemmler M, Emmerich P, Schmitz H, Varaine F, et al. Outbreaks of disease suspected of being due to human monkeypox virus infection in the Democratic Republic of Congo in 2001. *J Clin Microbiol*. 2002; 40(8): 2919-21.

21. Doshi RH, Alfonso VH, Morier D, Hoff NA, Sinai C, Mulembakani P, et al. Monkeypox rash severity and animal exposures in the Democratic Republic of the Congo. EcoHealth. 2020; 17(1): 64-73.

22. Rao AK, Schulte J, Chen TH, Hughes CM, Davidson W, Neff JM, et al. Monkeypox in a traveler returning from Nigeria - Dallas, Texas, July 2021. *MMWR Morb Mortal Wkly Rep*. 2022; 71(14): 509-16.

23. Yinka-Ogunleye A, Aruna O, Dalhat M, Ogoina D, McCollum A, Disu Y, et al. Outbreak of human monkeypox in Nigeria in 2017-18: a clinical and epidemiological report. *Lancet Infect dis*. 2019; 19(8): 872-9.

24. Ye F, Song J, Zhao L, Zhang Y, Xia L, Zhu L, et al. Molecular evidence of human Monkeypox virus infection, Sierra Leone. *Emerg Infect Dis*. 2019;25(6):1220-2.

25. Rimoin AW, Mulembakani PM, Johnston SC, Lloyd Smith JO, Kisalu NK, Kinkela TL, et al. Major increase in human monkeypox incidence 30 years after smallpox vaccination campaigns cease in the Democratic Republic of Congo. *Proc Natl Acad Sci U S A*. 2010; 107(37): 16262-7.

26. Likos AM, Sammons SA, Olson VA, Frace AM, Li Y, Olsen-Rasmussen M, et al. A tale of two clades: monkeypox viruses. *J Gen Virol*. 2005; 86(Pt 10): 2661-72.

27. Kalthan E, Tenguere J, Ndjapou SG, Koyazengbe TA, Mbomba J, Marada RM, et al. Investigation of an outbreak of monkeypox in an area occupied by armed groups, Central African Republic. *Med Mal Infect*. 2018; 48(4): 263-8.

28. Doshi RH, Guagliardo SAJ, Doty JB, Babeaux AD, Matheny A, Burgado J, et al. Epidemiologic and ecologic investigations of Monkeypox, Likouala department, Republic of the Congo, 2017. *Emerg Infect Dis*. 2019; 25(2): 281-9.

29. Eteng WE, Mandra A, Doty J, Yinka-Ogunleye A, Aruna S, Reynolds MG, et al. Notes from the field: responding to an outbreak of Monkeypox using the One Health approach - Nigeria, 2017-2018. *MMWR Morb Mortal Wkly Rep*. 2018; 67(37): 1040-1.

30. Ladnyj ID, Ziegler P, Kima E. A human infection caused by monkeypox virus in Basankusu Territory, Democratic Republic of the Congo. *Bull World Health Organ*. 1972; 46(5): 593-7.

31. Boumandouki P, Bileckot R, Ibara JR, Satounkazi C, Wassa Wassa D, Libama E, et al. [Simian smallpox (or monkey smallpox): study of 8 cases observed at Impfondo Hospital in Republic of Congo]. *Bull Soc Pathol Exot* (1990). 2007; 100(1): 17-21.

32. Costello V, Sowash M, Gaur A, Cardis M, Pasieka H, Wortmann G, et al. Imported Monkeypox from international traveler, Maryland, USA, 2021. *Emerg Infect Dis*. 2022; 28(5): 1002-5.

33. Adler H, Gould S, Hine P, Snell LB, Wong W, Houlihan CF, et al. Clinical features and management of human monkeypox: a retrospective observational study in the UK. *Lancet Infect dis*. 2022;22(8):1153-62.

34. Enserink M. Infectious diseases. U.S. monkeypox outbreak traced to Wisconsin pet dealer. *Science*. 2003; 300(5626): 1639.

35. Gispen R, Brand-Saathof BB, Hekker AC. Monkeypox-specific antibodies in human and simian sera from the Ivory Coast and Nigeria. *Bull World Health Organ*. 1976; 53(4): 355-60.

36. Croft DR, Sotir MJ, Williams CJ, Kazmierczak JJ, Wegner MV, Rausch D, et al. Occupational risks during a monkeypox outbreak, Wisconsin, 2003. *Emerg Infect Dis*. 2007; 13(8): 1150-7.

37. Yong SEF, Ng OT, Ho ZJM, Mak TM, Marimuthu K, Vasoo S, et al. Imported Monkeypox, Singapore. *Emerg Infect Dis*. 2020; 26(8): 1826-30.

38. Nakouné E, Kazanji M. Monkeypox detection in maculopapular lesions in two young Pygmies in the Central African Republic. *Int J Infect Dis*. 2012; 16: e266-e7.

39. Rimoin AW, Kisalu N, Kebela-Ilunga B, Mukaba T, Wright LL, Formenty P, et al. Endemic human monkeypox, Democratic Republic of Congo, 2001-2004. *Emerg Infect Dis*. 2007; 13(6): 934-7.

40. Sejvar JJ, Chowdary Y, Schomogyi M, Stevens J, Patel J, Karem K, et al. Human monkeypox infection: a family cluster in the midwestern United States. *J Infect Dis*. 2004; 190(10): 1833-40.

41. Marennikova SS, Seluhina EM, Mal'ceva NN, Cimiskjan KL, Macevic GR. Isolation and properties of the causal agent of a new variola-like disease (monkeypox) in man. *Bull World Health Organ*. 1972; 46(5): 599-611.

42. Whitehouse ER, Bonwitt J, Hughes CM, Lushima RS, Likafi T, Nguete B, et al. Clinical and epidemiological findings from enhanced Monkeypox surveillance in Tshuapa Province, Democratic Republic of the Congo During 2011-2015. *J Infect Dis*. 2021; 223(11): 1870-8.

43. Huhn GD, Bauer AM, Yorita K, Graham MB, Sejvar J, Likos A, et al. Clinical characteristics of human monkeypox, and risk factors for severe disease. *Clin Infect Dis*. 2005; 41(12): 1742-51.

44. Reed KD, Melski JW, Graham MB, Regnery RL, Sotir MJ, Wegner MV, et al. The detection of monkeypox in humans in the Western Hemisphere. *N Engl J Med*. 2004; 350(4): 342-50.

45. Vaughan A, Aarons E, Astbury J, Brooks T, Chand M, Flegg P, et al. Human-to-human transmission of Monkeypox virus, United Kingdom, October 2018. *Emerg Infect Dis*. 2020; 26(4): 782-5.

46. Besombes C, Gonofio E, Konamna X, Selekon B, Grant R, Gessain A, et al. Intrafamily transmission of Monkeypox virus, Central African Republic, 2018. *Emerg Infect Dis*. 2019; 25(8): 1602-4.

47. Monkeypox in the Democratic Republic of the Congo (former Zaire). *Releve epidemiologique hebdomadaire*. 1997; 72(34): 258.

48. Cunha BE. Monkeypox in the United States: an occupational health look at the first cases. *AAOHN J*. 2004;52(4):164-8.

49. Charatan F. US doctors investigate more than 50 possible cases of monkeypox. *BMJ* (Clinical research ed). 2003;326(7403):1350.

50. Macneil A, Reynolds MG, Braden Z, Carroll DS, Bostik V, Karem K, et al. Transmission of atypical varicella-zoster virus infections involving palm and sole manifestations in an area with monkeypox endemicity. *Clin infect dis*. 2009; 48(1): e6-8.

51. Ibegu M, Numbere T-W, Balogun M, Nguku P. Descriptive epidemiology of Monkeypox outbreak in Bayelsa State South-South Nigeria, November 2017. *Int J Infect Dis*. 2020; 101: 255.

52. Berthet N, Nakouné E, Whist E, Selekon B, Burguière AM, Manuguerra JC, et al. Maculopapular lesions in the Central African Republic. *Lancet*. 2011; 378(9799): 1354.

53. Human monkeypox in Kasai Oriental, Democratic Republic of the Congo (former Zaire). Preliminary report of October 1997 investigation. *Wkly Epidemiol Rec*. 1997; 72(49): 369-72.

54. Perez Duque M, Ribeiro S, Martins JV, Casaca P, Leite PP, Tavares M, et al. Ongoing monkeypox virus outbreak, Portugal, 29 April to 23 May 2022. *Euro Surveill*. 2022; 27(22): 2200424.

55. Osadebe L, Hughes CM, Shongo Lushima R, Kabamba J, Nguete B, Malekani J, et al. Enhancing case definitions for surveillance of human monkeypox in the Democratic Republic of Congo. *PLoS Negl Trop Dis*. 2017; 11(9): e0005857.

56. MacNeil A, Reynolds MG, Carroll DS, Karem K, Braden Z, Lash R, et al. Monkeypox or varicella? Lessons from a rash outbreak investigation in the Republic of the Congo. *Am J Trop Med Hyg*. 2009; 80(4): 503-7.

57. Müller G, Meyer A, Gras F, Emmerich P, Kolakowski T, Esposito JJ. Monkeypox virus in liver and spleen of child in Gabon. *Lancet*. 1988; 1(8588): 769-70.

58. Sadeuh-Mba SA, Yonga MG, Els M, Batejat C, Eyangoh S, Caro V, et al. Monkeypox virus phylogenetic similarities between a human case detected in Cameroon in 2018 and the 2017-2018 outbreak in Nigeria. Infection, genetics and evolution. *Infect Genet Evol*. 2019; 69: 8-11.

59. Ogoina D, Iroezindu M, James HI, Oladokun R, Yinka-Ogunleye A, Wakama P, et al. Clinical course and outcome of human Monkeypox in Nigeria. *Clin infect dis*. 2020; 71(8): e210-e4.

60. Learned LA, Reynolds MG, Wassa DW, Li Y, Olson VA, Karem K, et al. Extended interhuman transmission of monkeypox in a hospital community in the Republic of the Congo, 2003. *Am J Trop Med Hyg*. 2005; 73(2): 428-34.

61. Mukinda VB, Mwema G, Kilundu M, Heymann DL, Khan AS, Esposito JJ. Re-emergence of human monkeypox in Zaire in 1996. Monkeypox Epidemiologic Working Group. *Lance*t. 1997;349(9063):1449-50.

62. Mauldin MR, McCollum AM, Nakazawa YJ, Mandra A, Whitehouse ER, Davidson W, et al. Exportation of Monkeypox virus from the African Continent. *J Infect Dis*. 2022; 225(8): 1367-76.

63. Reynolds MG, Yorita KL, Kuehnert MJ, Davidson WB, Huhn GD, Holman RC, et al. Clinical manifestations of human monkeypox influenced by route of infection. *J Infect Dis*. 2006; 194(6): 773-80.

64. McCollum AM, Nakazawa Y, Ndongala GM, Pukuta E, Karhemere S, Lushima RS, et al. Human Monkeypox in the Kivus, a conflict region of the Democratic Republic of the Congo. *Am J Trop Med Hyg*. 2015; 93(4): 718-21.

65. Human monkeypox -- Kasai Oriental, Democratic Republic of Congo, February 1996-October 1997. *MMWR Morb Mortal Wkly Rep*. 1997; 46(49): 1168-71.

66. From the Centers for Disease Control and Prevention. Human monkeypox--Kasai Oriental, Democratic Republic of Congo, February 1996-October 1997. *Jama*. 1998; 279(3): 189-90.

67. Hobson G, Adamson J, Adler H, Firth R, Gould S, Houlihan C, et al. Family cluster of three cases of monkeypox imported from Nigeria to the United Kingdom, May 2021. *Euro surveillance*. 2021; 26(32): 2100745.

68. Update: multistate outbreak of monkeypox--Illinois, Indiana, Kansas, Missouri, Ohio, and Wisconsin, 2003. *MMWR Morb Mortal Wkly Rep*. 2003; 52(26): 616-8.

69. Human monkeypox--Kasai Oriental, Zaire, 1996-1997. *MMWR Morb Mortal Wkly Rep*. 1997; 46(14):3 04-7.

70. Hutin YJ, Williams RJ, Malfait P, Pebody R, Loparev VN, Ropp SL, et al. Outbreak of human monkeypox, Democratic Republic of Congo, 1996 to 1997. *Emerg Infect Dis*. 2001; 7(3): 434-8.

71. Heymann DL, Szczeniowski M, Esteves K. Re-emergence of monkeypox in Africa: a review of the past six years. *British Med Bull*. 1998; 54(3): 693-702.

72. Jezek Z, Szczeniowski M, Paluku KM, Mutombo M. Human monkeypox: clinical features of 282 patients. *J Infect Dis*. 1987; 156(2): 293-8.

73. Jezek Z, Marennikova SS, Mutumbo M, Nakano JH, Paluku KM, Szczeniowski M. Human monkeypox: a study of 2,510 contacts of 214 patients. *J Infect Dis*. 1986; 154(4): 551-5.

74. Jezek Z, Grab B, Szczeniowski M, Paluku KM, Mutombo M. Clinico-epidemiological features of monkeypox patients with an animal or human source of infection. *Bull World Health Organ*. 1988; 66(4): 459-64.

75. Meyer A, Esposito JJ, Gras F, Kolakowski T, Fatras M, Muller G. [First appearance of monkey pox in human beings in Gabon]. *Medecine tropicale : revue du Corps de sante colonial*. 1991; 51(1): 53-7.

76. Update: multistate outbreak of monkeypox--Illinois, Indiana, Kansas, Missouri, Ohio, and Wisconsin, 2003. *MMWR Morb Mortal Wkly Rep*. 2003; 52(24): 561-4.

77. Lewis MW, Graham MB, Hammarlund E, Hanifin J, Slifka MK. Monkeypox without exanthem. *N Engl J Med*. 2007; 356(20): 2112-4.

78. Edmiston CE, Jr., Graham MB, Wilson PJ, Grahn B. The monkeypox virus outbreak: reflections from the frontlines. *Am J Vet Res*. 2003; 31(6): 382-4.

79. Reynolds MG, Wauquier N, Li Y, Satheshkumar PS, Kanneh LD, Monroe B, et al. Human Monkeypox in Sierra Leone after 44-year absence of reported cases. *Emerg Infect Dis*. 2019; 25(5): 1023-5.

80. Faye O, Pratt CB, Faye M, Fall G, Chitty JA, Diagne MM, et al. Genomic characterisation of human monkeypox virus in Nigeria. *Lancet Infect dis*. 2018; 18(3): 246.

81. Petersen BW, Kabamba J, McCollum AM, Lushima RS, Wemakoy EO, Muyembe Tamfum JJ, et al. Vaccinating against monkeypox in the Democratic Republic of the Congo. *Antiviral Res*. 2019; 162: 171-7.

82. Lourie B, Bingham PG, Evans HH, Foster SO, Nakano JH, Herrmann KL. Human infection with monkeypox virus: laboratory investigation of six cases in West Africa. *Bull World Health Organ*. 1972; 46(5): 633-9.

83. Formenty P, Muntasir MO, Damon I, Chowdhary V, Opoka ML, Monimart C, et al. Human monkeypox outbreak caused by novel virus belonging to Congo Basin clade, Sudan, 2005. *Emerg Infect Dis*. 2010; 16(10): 1539-45.

84. Li D, Wilkins K, McCollum AM, Osadebe L, Kabamba J, Nguete B, et al. Evaluation of the genexpert for human Monkeypox diagnosis. *Am J Trop Med Hyg*. 2017; 96(2): 405-10.

85. Mutombo M, Arita I, Jezek Z. Human monkeypox transmitted by a chimpanzee in a tropical rain-forest area of Zaire. *Lancet*. 1983; 1(8327): 735-7.

86. Kaye DJCid. UK Healthcare Worker Contracts Rare Monkeypox Virus in Third Case. 2019(1):68.

87. Adnan N, Haq ZU, Malik A, Mehmood A, Ishaq U, Faraz M, et al. Human monkeypox virus: An updated review. *Medicine*. 2022; 101(35): e30406.

88. Arita I, Jezek Z, Khodakevich L, Ruti K. Human monkeypox: a newly emerged orthopoxvirus zoonosis in the tropical rain forests of Africa. *Am J Trop Med Hyg*. 1985;34(4): 781-9.

89. Breman JG, Kalisa R, Steniowski MV, Zanotto E, Gromyko AI, Arita I. Human monkeypox, 1970-79. *Bull World Health Organ*. 1980;58(2): 165-82.

90. Jezek Z, Grab B, Szczeniowski MV, Paluku KM, Mutombo M. Human monkeypox: secondary attack rates. *Bull World Health Organ*. 1988;66(4): 465-70.

91. Liu Y, Ye H, Zhang Y, Zhao K, Yang Z, Yuan Y, et al. Surface-tension-controlled crystallization for high-quality 2D perovskite single crystals for ultrahigh photodetection. *Matter.* 2019; 1(2): 465-80.

92. Akar S, Adesola YO, Akar S, Burga J, Oluwafemi B, Akinrogbe J, et al. Descriptive epidemiology of monkeypox in Nigeria, September 2017–June 2019. *Int J Infect Dis*. 2020;101: 219-20.

93. The L. Monkeypox contacts: a puzzling problem. *Lancet*. 2018; 392(10152): 986.

94. Nolen LD, Osadebe L, Katomba J, Likofata J, Mukadi D, Monroe B, et al. Introduction of Monkeypox into a community and household: risk factors and zoonotic reservoirs in the Democratic Republic of the Congo. *Am J Trop Med Hyg*. 2015; 93(2): 410-5.

95. Guarner J, Johnson BJ, Paddock CD, Shieh WJ, Goldsmith CS, Reynolds MG, et al. Monkeypox transmission and pathogenesis in prairie dogs. *Emerg Infect Dis*. 2004; 10(3): 426-31.

96. Human monkeypox in Kasai Oriental, Zaire (1996-1997). *Wkly Epidemiol Rec*. 1997; 72(15): 101-4.

97. Beer EM, Rao VB. A systematic review of the epidemiology of human monkeypox outbreaks and implications for outbreak strategy. *PLoS Negl Trop Dis*. 2019;13(10): e0007791.

98. The current status of human monkeypox: memorandum from a WHO meeting. *Bull World Health Organ*. 1984; 62(5): 703-13.

99. Khodakevich L, Jezek Z, Kinzanzka K. Isolation of monkeypox virus from wild squirrel infected in nature. *Lancet*. 1986; 1(8472): 98-9.

100. Radonić A, Metzger S, Dabrowski PW, Couacy-Hymann E, Schuenadel L, Kurth A, et al. Fatal monkeypox in wild-living sooty mangabey, Côte d'Ivoire, 2012. *Emerg Infect Dis*. 2014; 20(6): 1009-11.

101. Patrono LV, Pléh K, Samuni L, Ulrich M, Röthemeier C, Sachse A, et al. Monkeypox virus emergence in wild chimpanzees reveals distinct clinical outcomes and viral diversity. *Nat Microbiol*. 2020; 5(7): 955-65.

102. Rimoin AW, Alfonso VH, Hoff NA, Doshi RH, Mulembakani P, Kisalu NK, et al. Human exposure to wild animals in the Sankuru Province of the Democratic Republic of the Congo. *EcoHealth*. 2017; 14(3): 552-63.

103. Mandja BM, Brembilla A, Handschumacher P, Bompangue D, Gonzalez JP, Muyembe JJ, et al. Temporal and spatial dynamics of Monkeypox in Democratic Republic of Congo, 2000-2015. *EcoHealth*. 2019; 16(3): 476-87.

104. Fuller T, Thomassen HA, Mulembakani PM, Johnston SC, Lloyd-Smith JO, Kisalu NK, et al. Using remote sensing to map the risk of human monkeypox virus in the Congo Basin. *EcoHealth*. 2011; 8(1): 14-25.

105. Khodakevich L, Szczeniowski M, Manbu ma D, Jezek Z, Marennikova S, Nakano J, et al. The role of squirrels in sustaining monkeypox virus transmission. *Trop Geogr Med*. 1987; 39(2): 115-22.

106. McConnell S, Hickman RL, Wooding WL, Jr., Huxsoll DL. Monkeypox: experimental infection in chimpanzee (Pan satyrus) and immunization with vaccinia virus. *Am J Vet Res*. 1968; 29(8): 1675-80.

107. Khodakevich L, Jezek Z, Messinger D. Monkeypox virus: ecology and public health significance. *Bull World Health Organ*. 1988; 66(6): 747-52.

108. Orviz E, Negredo A, Ayerdi O, Vázquez A, Muñoz-Gomez A, Monzón S, et al. Monkeypox outbreak in Madrid (Spain): Clinical and virological aspects. *J Infect Dis*. 2022; 85(4): 412-7.

109. Hammerschlag Y, MacLeod G, Papadakis G, Adan Sanchez A, Druce J, Taiaroa G, et al. Monkeypox infection presenting as genital rash, Australia, May 2022. *Euro surveillance*. 2022;27(22): 2200411.

110. Vivancos R, Anderson C, Blomquist P, Balasegaram S, Bell A, Bishop L, et al. Community transmission of monkeypox in the United Kingdom, April to May 2022. Euro surveillance:. 2022;27(22):2200422.

111. WHO. Reported cases of human monkeypox in Africa, 1970–2018. https://apps.who.int/iris/bitstream/handle/10665/260497/WER9311.pdf;jsessionid=7AB72F28D04CFE6CE24996192FC478FF?sequence=1 (accessed May 31, 2022).

112. NCDC. An Update of Monkeypox Outbreak in Nigeria for Week 21. https://ncdc.gov.ng/diseases/sitreps/?cat=8&name=An%20Update%20of%20Monkeypox%20Outbreak%20in%20Nigeria (accessed May 31, 2022).

113. NCDC. An Update of Monkeypox Outbreak in Nigeria for Week 17. https://ncdc.gov.ng/diseases/sitreps/?cat=8&name=An%20Update%20of%20Monkeypox%20Outbreak%20in%20Nigeria (accessed May 31, 2022).

114. NCDC. An Update of Monkeypox Outbreak in Nigeria for Week 13. https://ncdc.gov.ng/diseases/sitreps/?cat=8&name=An%20Update%20of%20Monkeypox%20Outbreak%20in%20Nigeria (accessed May 31, 2022).

115. NCDC. An Update of Monkeypox Outbreak in Nigeria for Week 8. https://ncdc.gov.ng/diseases/sitreps/?cat=8&name=An%20Update%20of%20Monkeypox%20Outbreak%20in%20Nigeria (accessed May 31, 2022).

116. NCDC. An Update of Monkeypox Outbreak in Nigeria for Week 4. https://ncdc.gov.ng/diseases/sitreps/?cat=8&name=An%20Update%20of%20Monkeypox%20Outbreak%20in%20Nigeria (accessed May 31, 2022).

117. NCDC. An Update of Monkeypox Outbreak in Nigeria for Week 52. https://ncdc.gov.ng/diseases/sitreps/?cat=8&name=An%20Update%20of%20Monkeypox%20Outbreak%20in%20Nigeria (accessed May 31, 2022).

118. NCDC. An Update of Monkeypox Outbreak in Nigeria for Week 47. https://ncdc.gov.ng/diseases/sitreps/?cat=8&name=An%20Update%20of%20Monkeypox%20Outbreak%20in%20Nigeria (accessed May 31, 2022).

119. NCDC. An Update of Monkeypox Outbreak in Nigeria for Week 43. https://ncdc.gov.ng/diseases/sitreps/?cat=8&name=An%20Update%20of%20Monkeypox%20Outbreak%20in%20Nigeria (accessed May 31, 2022).

120. NCDC. An Update of Monkeypox Outbreak in Nigeria for Week 38. https://ncdc.gov.ng/diseases/sitreps/?cat=8&name=An%20Update%20of%20Monkeypox%20Outbreak%20in%20Nigeria (accessed May 31, 2022).

121. NCDC. An Update of Monkeypox Outbreak in Nigeria for Week 34. https://ncdc.gov.ng/diseases/sitreps/?cat=8&name=An%20Update%20of%20Monkeypox%20Outbreak%20in%20Nigeria (accessed May 31, 2022).

122. NCDC. An Update of Monkeypox Outbreak in Nigeria for Week 30. https://ncdc.gov.ng/diseases/sitreps/?cat=8&name=An%20Update%20of%20Monkeypox%20Outbreak%20in%20Nigeria (accessed May 31, 2022).

123. NCDC. An Update of Monkeypox Outbreak in Nigeria for Week 26. https://ncdc.gov.ng/diseases/sitreps/?cat=8&name=An%20Update%20of%20Monkeypox%20Outbreak%20in%20Nigeria (accessed May 31, 2022).

124. NCDC. An Update of Monkeypox Outbreak in Nigeria for Week 21. https://ncdc.gov.ng/diseases/sitreps/?cat=8&name=An%20Update%20of%20Monkeypox%20Outbreak%20in%20Nigeria (accessed May 31, 2022).

125. NCDC. An Update of Monkeypox Outbreak in Nigeria for Week 53. https://ncdc.gov.ng/diseases/sitreps/?cat=8&name=An%20Update%20of%20Monkeypox%20Outbreak%20in%20Nigeria (accessed May 31, 2022).

126. NCDC. An Update of Monkeypox Outbreak in Nigeria for Week 48. https://ncdc.gov.ng/diseases/sitreps/?cat=8&name=An%20Update%20of%20Monkeypox%20Outbreak%20in%20Nigeria (accessed May 31, 2022).

127. NCDC. An Update of Monkeypox Outbreak in Nigeria for Week 44. https://ncdc.gov.ng/diseases/sitreps/?cat=8&name=An%20Update%20of%20Monkeypox%20Outbreak%20in%20Nigeria (accessed May 31, 2022).

128. NCDC. An Update of Monkeypox Outbreak in Nigeria for Week 40. https://ncdc.gov.ng/diseases/sitreps/?cat=8&name=An%20Update%20of%20Monkeypox%20Outbreak%20in%20Nigeria (accessed May 31, 2022).

129. NCDC. An Update of Monkeypox Outbreak in Nigeria for Week 35. https://ncdc.gov.ng/diseases/sitreps/?cat=8&name=An%20Update%20of%20Monkeypox%20Outbreak%20in%20Nigeria (accessed May 31, 2022).

130. NCDC. An Update of Monkeypox Outbreak in Nigeria for Week 5. https://ncdc.gov.ng/diseases/sitreps/?cat=8&name=An%20Update%20of%20Monkeypox%20Outbreak%20in%20Nigeria (accessed May 31, 2022).

131. NCDC. An Update of Monkeypox Outbreak in Nigeria for Week 50. https://ncdc.gov.ng/diseases/sitreps/?cat=8&name=An%20Update%20of%20Monkeypox%20Outbreak%20in%20Nigeria (accessed May 31, 2022).

132. NCDC. An Update of Monkeypox Outbreak in Nigeria for Week 46. https://ncdc.gov.ng/diseases/sitreps/?cat=8&name=An%20Update%20of%20Monkeypox%20Outbreak%20in%20Nigeria (accessed May 31, 2022).

133. NCDC. An Update of Monkeypox Outbreak in Nigeria for Week 41. https://ncdc.gov.ng/diseases/sitreps/?cat=8&name=An%20Update%20of%20Monkeypox%20Outbreak%20in%20Nigeria (accessed May 31, 2022).

134. NCDC. An Update of Monkeypox Outbreak in Nigeria for Week 37. https://ncdc.gov.ng/diseases/sitreps/?cat=8&name=An%20Update%20of%20Monkeypox%20Outbreak%20in%20Nigeria (accessed May 31 2022).

135. NCDC. An Update of Monkeypox Outbreak in Nigeria for Week 9. https://ncdc.gov.ng/diseases/sitreps/?cat=8&name=An%20Update%20of%20Monkeypox%20Outbreak%20in%20Nigeria (accessed May 31, 2022).

136. NCDC. An Update of Monkeypox Outbreak in Nigeria for Week 4. https://ncdc.gov.ng/diseases/sitreps/?cat=8&name=An%20Update%20of%20Monkeypox%20Outbreak%20in%20Nigeria (accessed May 31, 2022).

137. NCDC. An Update of Monkeypox Outbreak in Nigeria for Week 52. https://ncdc.gov.ng/diseases/sitreps/?cat=8&name=An%20Update%20of%20Monkeypox%20Outbreak%20in%20Nigeria (accessed May 31, 2022).

138. NCDC. An Update of Monkeypox Outbreak in Nigeria for Week 50. https://ncdc.gov.ng/diseases/sitreps/?cat=8&name=An%20Update%20of%20Monkeypox%20Outbreak%20in%20Nigeria (accessed May 31, 2022).

139. NCDC. An Update of Monkeypox Outbreak in Nigeria for Week 48. https://ncdc.gov.ng/diseases/sitreps/?cat=8&name=An%20Update%20of%20Monkeypox%20Outbreak%20in%20Nigeria (accessed May 31, 2022).

140. NCDC. An Update of Monkeypox Outbreak in Nigeria for Week 48. https://ncdc.gov.ng/diseases/sitreps/?cat=8&name=An%20Update%20of%20Monkeypox%20Outbreak%20in%20Nigeria (accessed May 31, 2022).

141. NCDC. An Update of Monkeypox Outbreak in Nigeria for Week 45. https://ncdc.gov.ng/diseases/sitreps/?cat=8&name=An%20Update%20of%20Monkeypox%20Outbreak%20in%20Nigeria (accessed May 31, 2022).

142. NCDC. An Update of Monkeypox Outbreak in Nigeria for Week 43. https://ncdc.gov.ng/diseases/sitreps/?cat=8&name=An%20Update%20of%20Monkeypox%20Outbreak%20in%20Nigeria (accessed May 31, 2022).

143. NCDC. An Update of Monkeypox Outbreak in Nigeria for Week 43. https://ncdc.gov.ng/diseases/sitreps/?cat=8&name=An%20Update%20of%20Monkeypox%20Outbreak%20in%20Nigeria (accessed May 31, 2022).

144. NCDC. An Update of Monkeypox Outbreak in Nigeria for Week 42. https://ncdc.gov.ng/diseases/sitreps/?cat=8&name=An%20Update%20of%20Monkeypox%20Outbreak%20in%20Nigeria (accessed May 31, 2022).

145. WHO. Multi-country monkeypox outbreak: situation update. https://www.who.int/emergencies/disease-outbreak-news/item/2022-DON390 (accessed May 31, 2022).

146. WHO. Multi-country monkeypox outbreak in non-endemic countries: Update. https://www.who.int/emergencies/disease-outbreak-news/item/2022-DON388 (accessed May 31, 2022).

147. WHO. Multi-country monkeypox outbreak in non-endemic countries. https://www.who.int/emergencies/disease-outbreak-news/item/2022-DON385 (accessed May 31, 2022).

148. WHO. Monkeypox - United Kingdom of Great Britain and Northern Ireland. https://www.who.int/emergencies/disease-outbreak-news/item/2022-DON383 (accessed May 31, 2022).

149. WHO. Monkeypox - United Kingdom of Great Britain and Northern Ireland. https://www.who.int/emergencies/disease-outbreak-news/item/2022-DON381 (accessed May 31, 2022).

150. WHO. Monkeypox - United States of America. https://www.who.int/emergencies/disease-outbreak-news/item/2021-DON344 (accessed May 31, 2022).

151. WHO. Monkeypox - United States of America. https://www.who.int/emergencies/disease-outbreak-news/item/monkeypox---the-united-states-of-america (accessed May 31, 2022).

152. WHO. Monkeypox - United Kingdom of Great Britain and Northern Ireland. https://www.who.int/emergencies/disease-outbreak-news/item/monkeypox---united-kingdom-of-great-britain-and-northern-ireland (accessed May 31, 2022).

153. WHO. Monkeypox - Singapore. https://www.who.int/emergencies/disease-outbreak-news/item/16-may-2019-monkeypox-singapore-en (accessed May 31, 2022).

154. WHO. Monkeypox - United Kingdom of Great Britain and Northern Ireland. https://www.who.int/emergencies/disease-outbreak-news/item/monkeypox---united-kingdom-of-great-britain-and-northern-ireland-ex-nigeria (accessed May 31, 2022).

155. WHO. Monkeypox - Democratic Republic of the Congo. https://www.who.int/emergencies/disease-outbreak-news/item/1997_03_21c-en (accessed May 31, 2022).

156. WHO. Monkeypox - African Region (AFRO). https://www.who.int/emergencies/disease-outbreak-news/item/1997_04_14-en (accessed May 31, 2022).

157. WHO. Monkeypox - African Region (AFRO). https://www.who.int/emergencies/disease-outbreak-news/item/1997_04_24b-en (accessed May 31, 2022).

158. WHO. Monkeypox - Democratic Republic of the Congo. https://www.who.int/emergencies/disease-outbreak-news/item/1997_07_31b-en (accessed May 31, 2022).

159. WHO. Monkeypox - Democratic Republic of the Congo. https://www.who.int/emergencies/disease-outbreak-news/item/1997_11_14b-en (accessed May 31, 2022).

160. WHO. Monkeypox - Nigeria. https://www.who.int/emergencies/disease-outbreak-news/item/21-december-2017-monkeypox-nigeria-en (accessed May 31, 2022).

161. WHO. Monkeypox - Cameroon. https://www.who.int/emergencies/disease-outbreak-news/item/05-june-2018-monkeypox-cameroon-en (accessed May 31, 2022).

162. WHO. Monkeypox - Nigeria. https://www.who.int/emergencies/disease-outbreak-news/item/05-october-2018-monkeypox-nigeria-en (accessed May 31, 2022).

163. WHO. Monkeypox - Democratic Republic of the Congo. https://www.who.int/emergencies/disease-outbreak-news/item/monkeypox-democratic-republic-of-the-congo (accessed May 31, 2022).

164. WHO. Emergence of monkeypox in West Africa and Central Africa, 1970–2017. https://www.who.int/publications/i/item/10665-260497 (accessed May 31, 2022).

165. WHO. Monkeypox - Central African Republic. https://www.who.int/emergencies/disease-outbreak-news/item/13-october-2016-monkeypox-caf-en (accessed May 31, 2022).

166. WHO. Weekly Bulletin on Outbreak and other Emergencies: Week 11: 09 - 15 March 2020. https://apps.who.int/iris/handle/10665/331451 (accessed May 31, 2022).

167. WHO. Weekly Bulletin on Outbreak and other Emergencies: Week 31: 29 July—04 August 2019. https://apps.who.int/iris/handle/10665/326159?search-result=true&query=Weekly+Bulletin+on+Outbreak+and+other+Emergencies%3A+Week+31%3A+29+July-04+August+2019&scope=&rpp=10&sort_by=score&order=desc (accessed May 31, 2022).

168. CDC. U.S. Monkeypox 2022: Situation Summary. https://www.cdc.gov/poxvirus/monkeypox/response/2022/index.html (accessed May 31, 2022).

169. CDC. Monkeypox in the United States. https://www.cdc.gov/poxvirus/monkeypox/outbreak/us-outbreaks.html (accessed May 31, 2022).

170. CDC. Travel-Associated Monkeypox virus infection confirmed in Maryland resident. https://health.maryland.gov/newsroom/Pages/Travel-Associated-Monkeypox-virus-infection-confirmed-in-Maryland-resident.aspx (accessed May 31, 2022).

171.Nakazawa Y, Emerson GL, Carroll DS, Zhao H, Li Y, Reynolds MG, et al. Phylogenetic and ecologic perspectives of a monkeypox outbreak, southern Sudan, 2005. *Emerg Infect Dis*. 2013; 19(2): 237-45.

172. Levine RS, Peterson AT, Yorita KL, Carroll D, Damon IK, Reynolds MG. Ecological niche and geographic distribution of human monkeypox in Africa. *PloS One*. 2007; 2(1): e176.

173. Nakazawa Y, Mauldin MR, Emerson GL, Reynolds MG, Lash RR, Gao J, et al. A phylogeographic investigation of African monkeypox. *Viruses*. 2015; 7(4): 2168-84.

174. Arita I, Henderson DA. Monkeypox and whitepox viruses in West and Central Africa. *Bull World Health Organ*. 1976; 53(4): 347-53

175. Breman JG, Nakano JH, Coffi E, Godfrey H, Gautun JC. Human poxvirus disease after smallpox eradication. *Am J Trop Med Hyg*. 1977; 26(2): 273-81

176.Khodakevich L, Szczeniowski M, Manbu ma D, Jezek Z, Marennikova S, Nakano J, et al. The role of squirrels in sustaining monkeypox virus transmission. *Trop Geogr Med*. 1987; 39(2): 115-22.

177. Khodakevich L, Szczeniowski M, Nambu ma D, Jezek Z, Marennikova S, Nakano J, et al. Monkeypox virus in relation to the ecological features surrounding human settlements in Bumba zone, Zaire. *Trop Geogr Med*. 1987; 39(1): 56-63.

178. Khodakevich L, Widy-Wirski R, Arita I, Marennikova SS, Nakano J, Meunier D. [Monkey pox virus infection in humans in the Central African Republic]. *Bull Soc Pathol Exot et de ses filiales*. 1985; 78(3): 311-20.

179. Pittman P, Martin J, Placide M, Muyembe J-J, Wan Q, Reynolds M, et al. Clinical characterization of human monkeypox infections in the Democratic Republic of the Congo. *PLoS Negl Trop Dis*. 2023; 17(4): e0010384.

180. Moundjoa C, Amabo F, Nanseu R, Metomb S, Abah AA, McCollum A, et al. Investigation and prevention against Monkeypox in Mefou Primate Sanctuary, Cameroon, 2016. *Trop Med Int Health*. 2017; 22: 52-3.

181. WHO. Weekly Bulletin on Outbreaks and Other Emergencies. Week 16: 15—21 April 2017. http://apps.who.int/iris/bitstream/handle/10665/255050/OEW16-152142017.pdf;jsessionid=194584C7F298C4F3B1307F5C21581531?sequence=1 (accessed July 25 2022).
